# O-GlcNAcylation of YAP1 promotes lung transplant ischemia-reperfusion injury via binding to HIF1α transcription factor and activating autophagy and mitophagy

**DOI:** 10.1038/s41419-026-08548-w

**Published:** 2026-03-15

**Authors:** Shaohua Dai, Xuemei Wan, Lingchun Xia, Lei Xu, Chunfan Xie, Guohui Wang, Jian Tang

**Affiliations:** 1 Department of Thoracic Surgery, Nanfang Hospital, Southern Medical University, 510515 Guangzhou, China; 2 Department of Thoracic Surgery, The First Affiliated Hospital, Jiangxi Medical College, Nanchang University, 330006 Nanchang, China; 3 Department of Cardiovascular and Vascular Surgery, The First Affiliated Hospital of Nanchang University, 330006 Nanchang, China

**Keywords:** Post-translational modifications, Cardiovascular diseases

## Abstract

Lung transplant ischemia-reperfusion injury poses a significant challenge in transplantation medicine, often causing severe complications and poor patient outcomes. Our study focused on the role of O-GlcNAcylation of Yes-associated protein 1 (YAP1) in exacerbating this injury by regulating autophagy and mitochondrial autophagy pathways. We found that hypoxia-reoxygenation robustly activated the Hippo-YAP1 signaling pathway, leading to increased damage in lung epithelial cells. Concurrently, autophagy and mitochondrial autophagy levels were significantly upregulated, indicating cellular stress responses. During actual lung transplantation, ischemia-reperfusion resulted in a marked increase in autophagy and mitochondrial autophagy levels, accompanied by elevated tissue damage. Notably, YAP1 played a crucial role in orchestrating these processes, as its knockdown reduced autophagy and mitochondrial autophagy levels under both hypoxia-reoxygenation and ischemia-reperfusion conditions. We further elucidated that OGT-mediated O-GlcNAc modification of YAP1 enhanced its interaction with HIF1α, activating downstream hypoxia-responsive molecules. Knockdown of the key enzyme OGT significantly mitigated autophagy, mitophagy, and associated damage in lung epithelial cells and transplant tissues subjected to hypoxia-reoxygenation and ischemia-reperfusion. These findings reveal the intricate interplay between O-GlcNAcylation of YAP1, HIF1α binding, autophagy activation, and mitochondrial autophagy in driving lung transplant ischemia-reperfusion injury, suggesting potential therapeutic targets for ameliorating its detrimental effects.

## Introduction

Lung transplantation is a life-saving therapeutic option for patients with end-stage lung diseases [1–5]. Despite significant advances in surgical techniques and post-transplant care, ischemia-reperfusion injury (IRI) remains a significant obstacle that affects graft function and patient outcomes [6–9]. IRI occurs when the transplanted lung is deprived of blood flow during procurement and storage, followed by reintroduction of blood flow during implantation [10–12]. This process leads to a cascade of cellular and molecular events, including oxidative stress, inflammation, and cell death, which contribute to graft dysfunction and potential rejection [13].

One of the critical signaling pathways implicated in the pathogenesis of IRI is the Hippo-YAP1 (Yes-associated protein 1) signaling pathway [14]. The Hippo pathway is a highly conserved signaling cascade that regulates organ size, tissue homeostasis, and regeneration [15]. The core components of the Hippo pathway include MST1/2 (mammalian Ste20-like protein kinases 1/2), SAV1 (Salvador homolog 1), LATS1/2 (large tumor suppressor kinases 1/2), and MOB1 (MOB kinase activator 1) [16, 17]. In response to various stimuli, such as cell-cell contact, mechanical stress, and energy status, the Hippo pathway regulates the phosphorylation and nuclear exclusion of YAP1, a transcriptional co-activator [18]. When the Hippo pathway is inactive, YAP1 translocate to the nucleus where it interacts with transcription factors, such as TEADs (TEA domain family members), to promote the expression of genes involved in cell proliferation, survival, and metabolism [19].

Emerging evidence suggests that YAP1 plays a pivotal role in the pathogenesis of various disease states, including cancer, fibrosis, and tissue injury [14, 15, 20–27]. In the context of lung IRI, YAP1 has been shown to be activated and contribute to the development of lung injury. However, the precise mechanisms by which YAP1 promotes lung IRI remain poorly understood [28]. Autophagy and mitochondrial autophagy (mitophagy) are two critical cellular processes that play important roles in maintaining cellular homeostasis and adapting to stress conditions [24]. Autophagy involves the degradation of cytoplasmic components, including organelles and protein aggregates, through the formation of double-membraned autophagosomes that fuse with lysosomes for degradation [29, 30]. Mitophagy, a specialized form of autophagy, targets damaged or dysfunctional mitochondria for degradation [31–34]. Both autophagy and mitophagy are essential for cellular survival and adaptation to stress, such as hypoxia and nutrient deprivation [35–37]. Recent studies have implicated autophagy and mitophagy in the pathogenesis of lung IRI [38, 39]. For example, autophagy has been shown to be activated in response to ischemia-reperfusion and to play a protective role by removing damaged organelles and proteins [40]. Conversely, excessive or dysregulated autophagy can contribute to cell death and tissue damage [41, 42]. Similarly, mitophagy has been shown to be important for maintaining mitochondrial quality control and preventing the accumulation of damaged mitochondria, which can release pro-apoptotic factors and exacerbate tissue injury.

The transcription factor HIF1α (hypoxia-inducible factor 1α) is a master regulator of the cellular response to hypoxia [43]. Under normoxic conditions, HIF1α is hydroxylated by prolyl hydroxylase domain-containing proteins (PHDs) and targeted for proteasomal degradation. However, under hypoxic conditions, PHD activity is inhibited, leading to the stabilization and nuclear translocation of HIF1α [44]. In the nucleus, HIF1α forms a heterodimer with HIF1β and binds to hypoxia-responsive elements (HREs) in the promoter regions of target genes to induce their expression [45]. HIF1α-targeted genes encode proteins involved in angiogenesis, glucose metabolism, erythropoiesis, and other adaptive responses to hypoxia. Interestingly, there is growing evidence for crosstalk between the Hippo-YAP1 and HIF1α signaling pathways. For example, YAP1 has been shown to interact with HIF1α and enhance its transcriptional activity, leading to the induction of HIF1α-target genes. Conversely, HIF1α has been shown to regulate the expression of Hippo pathway components, such as MST1/2 and LATS1/2, suggesting a reciprocal relationship between these two signaling pathways.

Post-translational modifications (PTMs) play critical roles in regulating protein function and signaling pathways [46]. One such PTM is O-GlcNAcylation, the addition of a single O-linked β-N-acetylglucosamine (O-GlcNAc) moiety to serine or threonine residues of proteins. O-GlcNAcylation is catalyzed by O-GlcNAc transferase (OGT) and removed by O-GlcNAcase (OGA) [47]. O-GlcNAcylation has been implicated in the regulation of numerous cellular processes, including transcription, translation, and signaling. Recent studies have shown that O-GlcNAcylation of YAP1 can regulate its subcellular localization, transcriptional activity, and interaction with other proteins [48].

In this study, we aimed to investigate the role of O-GlcNAcylation of YAP1 in promoting lung transplant IRI. Specifically, we hypothesized that hypoxia-reoxygenation (H/R), a model of IRI, activates the Hippo-YAP1 signaling pathway and induces autophagy and mitophagy in lung epithelial cells. We further hypothesized that O-GlcNAcylation of YAP1 enhances its binding to HIF1α and activation of downstream hypoxia-responsive molecules, leading to increased autophagy and mitophagy and exacerbating lung injury. To test these hypotheses, we conducted a series of experiments using lung epithelial cell lines and a mouse model of lung transplant IRI.

## Materials and methods

### Ethics statement

Approval for the study protocols and animal experimentation was granted by the Animal Ethics Committee of the First Affiliated Hospital of Nanchang University. Significant efforts were made to reduce the utilization and distress of the animals involved in the research.

### PMVEC cell culture and H/R injury model

The pulmonary microvascular endothelial cells (PMVECs) were acquired from Cell Biologics Inc., located in Chicago, IL, USA. In vitro, the cell models for H/R injury were established using a technique outlined in prior publications. In short, mouse PMVECs were suspended in M-1168 (Cell Biologics, Chicago, IL) and incubated at 37 °C in a 5% CO_2_–95% air environment. Based on the rate of cell growth, the density of cells was modified and then they were placed in 6-well plates. When the cells reached 80%–90% confluence, they were transfected with the Lipofectamine 2000 reagent (Invitrogen, Carlsbad, CA). Following H/R treatment, the cells were then transfected with an adenovirus knockdown YAP1 or OGT (Genelily Biotehc, Shanghai, China). Following a 6-h transfection period, the medium was substituted, and subsequently, the cells were cultivated for an additional 36 h. Afterwards, the cells were incubated in a low-oxygen environment with 95% N_2_, 4% CO_2_, and 1% O_2_ for 2, 4, 6 and 12 h. Subsequently, they were transferred to a regular cell incubator and cultured for an additional 4 h to establish a H/R cell model. Following this, cellular samples were gathered from every group for further experimentation.

### Real-time qPCR

The TRIzol reagent was used to extract the total RNA content from both lung tissues and cells. Using a Nano-Drop 2000 spectrophotometer (Thermo Fisher Scientific, Rockford, IL), the RNA concentration was measured. To reverse transcribe the RNA into cDNA, we utilized the ReverTra Ace qPCR RT Master Mix with gDNA remover kit from Toyobo Co., Ltd. in Osaka, Japan. Every inquiry consisted of a minimum of three wells, with each well-being replicated three times. Sangon Biotech Co., Ltd. (Shanghai, China) synthesized the primers for *YAP1, CTGF, CYR61, AREG, BIRC5, FUND1, PINK1, TBK1, HMGB1, DAPK*, and *OGT*. Glyceraldehyde-3-phosphate dehydrogenase (GAPDH) were used as internal references. Afterward, RT-qPCR was conducted following the guidelines of the 2 × Real-Star Green Fast Mixture Kit (Genstar, China). The fold changes were determined using the method of relative quantification, specifically the 2^–ΔΔCt^ approach.

### Western blot analysis

Cells and lung tissues from each group were collected and lysed on ice for 5 min using radio-immunoprecipitation assay lysis buffer (provided by Shanghai Beyotime Biotechnology Co. Ltd., Shanghai, China). The supernatant was obtained after being subjected to centrifugation at a speed of 12,000 revolutions per minute and a temperature of 4 degrees Celsius. Next, the bicinchoninic acid (Pierce, Thermo Fisher Scientific, Waltham, MA) method was employed to ascertain the protein concentration. The protein was isolated by employing a 4% separation gel and 10% spacer gel and subsequently transferred onto a membrane made of polyvinylidene fluoride. Afterward, the membrane underwent treatment with 5% skim milk powder at room temperature for 1 h. It was then incubated overnight at 4 °C with primary antibodies against YAP1 (Cat No. 13584-1-AP, 1:1500), p-YAP1 (Cat No. 80694-2-RR, 1:2000), HIF-1α (Cat No. 66730-1-Ig, 1:1000), CYR61 (No. 26689-1-AP, 1:1500), AREG (Cat No. 16036-1-AP, 1:2300), BIRC5 (Cat No. 10508-1-AP, 1:1200), FUNDC1 (Cat No. 28519-1-AP, 1:1500), PINK1 (Cat No. 23274-1-AP, 1:2000), TBK1 (Cat No. 28397-1-AP, 1:1500), HMGB1 (Cat No. 10829-1-AP, 1:1000), DAPK (Cat No. 25136-1-AP, 1:1000), LC3-I/II (Cat No. 14600-1-AP, 1:3000), OGT (Cat No. 11576-2-AP, 1:2000) and O-GlcNAc (Cat No. 65292-1-Ig, 1:3000) (all obtained from Proteintech, Wuhan, China). After the incubation period, the membrane underwent retesting using a secondary antibody IgG (Santa Cruz Biotechnology, Inc, Santa Cruz, CA) labeled with horseradish peroxidase. This step was carried out at room temperature for 1 h following a wash with Tris-buffered saline Tween-20, also at room temperature. Subsequently, the membrane was observed utilizing an enhanced chemiluminescence reaction provided by Thermo Fisher Scientific located in Rockford, IL. The protein bands’ volume was measured using a Bio-Rad ChemiDoc EQ densitometer and the Bio-Rad Quantity One v4.6.2 software. The protein expression was indicated by the ratio of the target band’s gray value to that of glyceraldehyde-3-phosphate dehydrogenase (Abcam Inc., Cambridge, MA).

### TUNEL (Terminal deoxynucleotidyl transferase dUTP nick end labeling)

The TUNEL method is a biochemical assay used to detect DNA fragmentation, which is a hallmark of apoptotic cell death. It specifically identifies cells undergoing apoptosis by labeling the free 3’-hydroxyl termini of DNA strand breaks. Ung epithelial cells were subjected to hypoxia-reoxygenation conditions for varying durations (0, 2, 4, 6, and 12 h). After the respective treatment times, cells were fixed with 4% paraformaldehyde and permeabilized to allow access to the cellular DNA. The TUNEL reaction mixture, containing terminal deoxynucleotidyl transferase (TdT) and labeled fluorescein-dUTP, was added to the cells. TdT catalyzes the incorporation of labeled nucleotides at the 3’-OH ends of DNA fragments, thus labeling apoptotic cells. The labeled cells were then visualized using fluorescence microscopy or flow cytometry. The intensity of fluorescence or the percentage of labeled cells provided a quantitative measure of apoptosis.

### ELISA (enzyme-linked immunosorbent assay)

ELISA is a biochemical assay used to detect and quantify specific proteins in a sample. It employs antibodies to target and measure the levels of specific analytes, such as inflammatory cytokines. Cell supernatants from lung epithelial cells subjected to hypoxia-reoxygenation for varying durations (0, 2, 4, 6, and 12 h) were collected. Microtiter plates were coated with a capture antibody specific for the inflammatory cytokines of interest (IL-10, MCP1, and ICAM1). The collected supernatants were added to the plates, allowing the cytokines to bind to the capture antibody. A detection antibody specific for the cytokines, often conjugated to horseradish peroxidase, was added. This antibody binds to the cytokine, forming a sandwich complex. A substrate for the enzyme was added, resulting in the production of a colored product. The intensity of the color was measured spectrophotometrically, which is directly proportional to the amount of cytokine present in the sample.

### Mitophagy assay by immunofluorescence staining

PMVECs were treated with Mito-Tracker Red CMXRos from Shanghai Beyotime Biotechnology Co. Ltd., Shanghai, China for 30 min. After that, they were fixed using 4% paraformaldehyde and permeabilized with 0.3% Triton X-100. Bovine serum albumin (BSA) was applied to block the cells. Next, the cells were incubated overnight at 4 °C with rabbit anti-LC3B antibody (ab51520, 1:2000, Abcam). Finally, the cells were incubated for another hour at ambient temperature with Alexa Fluor 488-labeled donkey anti-rabbit IgG secondary antibody (A21206, 1:500, Thermo Fisher Scientific, Waltham, MA, USA). Subsequently, we employed the confocal laser microscope (LSM800, Zeiss, Germany) for cell observation. A total of 200 or more cells were computed from each section to establish the ratio of Mitotracker-Red and LC3 spot cells (mitophagy).

### Transmission electron microscopy (TEM)

Transmission electron microscopy (TEM) was used to visualize the ultrastructure of cells and organelles at high resolution. Lung epithelial cells subjected to hypoxia-reoxygenation for varying durations (0, 2, 4, 6, and 12 h) were fixed using a chemical fixative such as glutaraldehyde, which preserves the cellular ultrastructure. The fixed cells were then post-fixed with osmium tetroxide, which enhances the contrast and stability of cellular components. The cells were dehydrated through a series of ethanol washes to gradually remove water, preparing them for embedding. The dehydrated cells were embedded in a resin such as epoxy, which solidifies to form a hard block. Thin sections (50 nm) were cut from the resin block using an ultramicrotome. The sections were stained with heavy metals like uranium acetate and lead citrate, which enhance the contrast of cellular components under the electron beam. The stained sections were mounted on grids and inserted into the TEM. High-resolution images of the cellular ultrastructure were captured, allowing for detailed examination of organelles, including mitochondria and autophagosomes.

### Chromatin immunoprecipitation (ChIP)

Alveolar epithelial cells subjected to hypoxia-reoxygenation for 12 h were fixed with formaldehyde to crosslink proteins to DNA. The cells were then lysed to release the chromatin, which was fragmented into smaller pieces using physical or enzymatic methods. The fragmented chromatin was incubated with an antibody specific to the HIF1A transcription factor (Cat No. 20960-1-AP, 1:200, Proteintech, Wuhan, China). The antibody-chromatin complexes were then precipitated using protein A/G agarose beads. The precipitated chromatin was washed to remove unbound proteins and other contaminants. Specific DNA sequences were amplified and quantified using qPCR techniques to determine the binding level of HIF1A to the promoters of autophagy and mitophagy genes (*HMGB1*, *DAPK, LC3-II, FUNDC1, PINK1, TBK1*).

### Co-Immunoprecipitation (CoIP)

Alveolar epithelial cells subjected to hypoxia-reoxygenation for varying durations (0, 2, 4, 6, 12 h) or ischemia-reperfusion (2, 6, 12 h) were lysed to release cellular proteins. The cell lysates were incubated with an antibody specific to the YAP1 protein (Cat No. 13584-1-AP, 1:400, Proteintech, Wuhan, China). The antibody-protein complexes were then precipitated using protein A/G agarose beads. The precipitated proteins were washed to remove unbound proteins and other contaminants. The bound proteins were then eluted and analyzed by Western blot using antibodies specific to O-GlcNAc and HIF1A to detect the O-GlcNAc modification level of YAP1 and its binding to HIF1A, respectively.

To investigate the O-GlcNAcylation and protein interactions of YAP1, Flag-YAP1 protein immunoprecipitation was performed using M2 Magnetic Beads. Alveolar epithelial cells, subjected to a 12-h hypoxia-reoxygenation cycle, were transfected to express wild-type or mutant constructs of OGT and Flag-YAP1. Following treatment, cells were lysed and the clarified supernatants were incubated with anti-Flag M2 Magnetic Beads to specifically isolate the Flag-tagged YAP1 protein and its associated complexes. After extensive washing to remove non-specifically bound proteins, the immunoprecipitated proteins were eluted. The resulting eluates were then analyzed by western blotting to assess the O-GlcNAc modification level of YAP1 using a specific antibody and to probe for co-precipitated HIF1A transcription factor, thereby evaluating the interaction between YAP1 and HIF1A under the specified experimental conditions.

### Dual-luciferase reporter assay

The luciferase report vectors (Genelily Biotechnology Co., Ltd., Shanghai, China) were used to introduce the predicted fragments of Numb with miR-485, including the 3′-untranslated region (UTR), binding site, and mutation fragments, as report plasmids Numb-wild-type (WT) and Numb-mutant. Next, Numb mRNA luciferase report plasmids were co-transfected with NC and miR-485 mimic to investigate the potential binding between Numb and miR-485.Following a 48-h period of transfection, the cells were gathered and subjected to lysis. Following steps were conducted utilizing a luciferase assay kit (K801-200, Biovision, Bay Area, San Francisco, CA) with the dual-luciferase reporter gene analysis system (Promega Corporation, Madison, WI).

### Rats model establishment of lung transplantation I/R injury

Adult male or female rats (commonly Sprague-Dawley rats) weighing approximately 250–300 g are used. Rats are housed under standard laboratory conditions with controlled temperature, humidity, and a 12-h light/dark cycle. They have access to standard rat chow and water ad libitum. The donor rat is anesthetized using an appropriate anesthetic agent, such as pentobarbital sodium (50 mg/kg, IP). A tracheostomy is performed to facilitate ventilation. Heparin (500 U/kg, IV) is administered to prevent blood clotting during the procedure. A median sternotomy is performed to expose the heart and lungs. The aorta is clamped just distal to the brachiocephalic trunk, and the pulmonary artery is clamped. The heart is then excised, leaving the lungs attached to the trachea. The lungs are flushed with cold Euro-Collins solution via the pulmonary artery to remove residual blood and to induce cold ischemia. The lungs are then stored in cold preservation solution at 4 °C for a specified period (1 h) to mimic the ischemic phase. The recipient rat is anesthetized similarly to the donor. A tracheostomy is performed, and the rat is ventilated with room air or oxygen-enriched air. A median sternotomy is performed to expose the heart and lungs. The recipient’s lungs are removed, ensuring to preserve the trachea, pulmonary vessels, and bronchial tree. The donor lungs are positioned in the recipient’s thoracic cavity. The pulmonary artery and bronchus are anastomosed using microsurgical techniques the left atrium is anastomosed to allow venous drainage. Once the anastomoses are complete, the clamps are removed, allowing reperfusion of the transplanted lungs. Respiratory rate, tidal volume, and oxygen saturation are monitored to assess lung function. Lung tissue samples are collected at specified time points for histological examination to assess tissue damage, inflammation, and other pathological changes. Blood and bronchoalveolar lavage fluid (BALF) samples are collected to measure biomarkers of lung injury, such as cytokines, chemokines, and enzymes. Lung function tests, such as pulmonary mechanics and gas exchange measurements, may be performed to quantify the degree of I/R injury.

### OGT and YAP1 knockdown in vitro and in vivo

siRNA sequences targeting OGT (siOGT-1 and siOGT-2) and shRNA sequences targeting OGT (shOGT-1 and shOGT-2) and YAP1 (shYAP1-1 and shYAP1-2) were designed as follows: siOGT-1: 5’-ACC TTG GCA ATT AAA CAG AAT-3’; siOGT-2: 5’-GGA AGC AAT TGA GCA TTA TCG-3’; shOGT-1: 5’- ACC TCA CCT TGG CAA TTA AAC AGA ATT CAA GAG ATT CTG TTT AAT TGC CAA GGT TT-3’; shOGT-2: 5’-ACC TCG GAA GCA ATT GAG CAT TAT CGT CAA GAG CGA TAA TGC TCA ATT GCT TCC TT-3’; shYAP1-1: 5’- ACC TCA CAC TGG AAG GAG ATG GAA TGT CAA GAG CAT TCC ATC TCC TTC CAG TGT TT-3’; shYAP1-2: 5’-ACC TCG CAT CTT CGA CAG TCT TCT TTT CAA GAG AAA GAA GAC TGT CGA AGA TGC TT-3’. For siRNA transfection, pulmonary microvascular endothelial cells (PMVECs) were cultured in antibiotic-free DMEM/F12 medium (Gibco) at 60–80% confluency. Transfection was performed using Lipofectamine RNAiMAX (Thermo Fisher) according to the manufacturer’s protocol. Briefly, 5 nM of siOGT-1, siOGT-2, or non-targeting control siRNA (siNT) was mixed with 0.2 µL of RNAiMAX reagent per well (96-well plate) in Opti-MEM (Gibco), incubated for 5 min at room temperature, and added to PMVECs. Cells were incubated for 48 h at 37 °C before subsequent experiments. For in vivo studies, adenoviruses expressing shOGT-1 or shOGT-2 were generated using the pAdEasy system (Agilent). shRNA cassettes were cloned into the pAdTrack-CMV shuttle vector under the U6 promoter. Recombinant adenoviruses were packaged in HEK293A cells and purified via CsCl gradient centrifugation. Titers were determined by plaque assay ( ≥ 1×10^10 PFU/mL). For delivery, rats received intratracheal administration of 100 µL adenovirus (shOGT or non-targeting control) 48 h prior to ischemia-reperfusion.

### Detection of lung wet weight-to-dry weight ratio

At first, one out of every three tissues from the left lung were selected, and its weight was measured using an OHAUS-Precision electronic balance (wet weight) before being recorded. Afterward, the tissues were placed inside an oven set at a temperature of 60 °C for a duration of 72 h until reaching a stable weight, after which the dry weight was measured. Finally, the wet weight-to-dry weight (W/D) ratio was computed to indicate the swelling of the lung tissues.

### Hematoxylin-eosin staining

Lung tissues were fixed, dehydrated, paraffin-embedded, and sliced into 5-μm-thick sections. Next, the sections were placed on a prepared slide, which was then put in a 60 °C incubator overnight. The sections were subsequently dewaxed, immersed in xylene I for 20 min, xylene II for 20 min, rehydrated with gradient alcohol (100%, 95%, 80%, and 70%, 5 min each), rinsed with 0.01 M PBS 3 times (5 min each), and stained with hematoxylin for 2-3 min (nuclei), followed by 3 rinses with PBS (5 min each). Thereafter, the sections were immersed in 1% hydrochloric acid-ethanol for 2–3 s, rinsed with PBS 3 times (5 min each), stained with eosin for 1 min, and then rinsed again with PBS 3 times (5 min each). Afterward, the sections were dehydrated with alcohol (70%, 80%, 95%, and 100%, 5 min each), cleared with xylene for 10 min, dried, and sealed using neutral gum. Two uninformed investigators performed the histological analysis under a microscope according to the standard of Lung Injury Score, and the final score was determined by the average value of the 2 investigators. The Lung Injury Score consists of grades (0–4), with 0 being normal and 4 indicating severe injury, including alveolar hyperemia, hemorrhage, interstitial edema, neutrophil count, and alveolar wall thickness.

### Statistical analysis

The SPSS 21.0 statistical software (IBM Corp. Armonk, NY) was utilized for processing all the data. The measurement data were represented as the average plus or minus the standard deviation. When the data followed a normal distribution and had homogeneity of variance, the comparisons between two unpaired groups were analyzed using an unpaired *t*-test. The analysis involved comparing multiple groups using one-way and two-way ANOVA along with Tukey’s post hoc test. The correlation coefficient of Pearson was utilized to examine the connection among the indicators. A significance level of *P* < 0.05 was deemed statistically significant.

## Results

### Hypoxia-reoxygenation significantly activates Hippo-YAP1 signaling pathway activity and mitophagy in lung epithelial cells

**Fig. 1:**
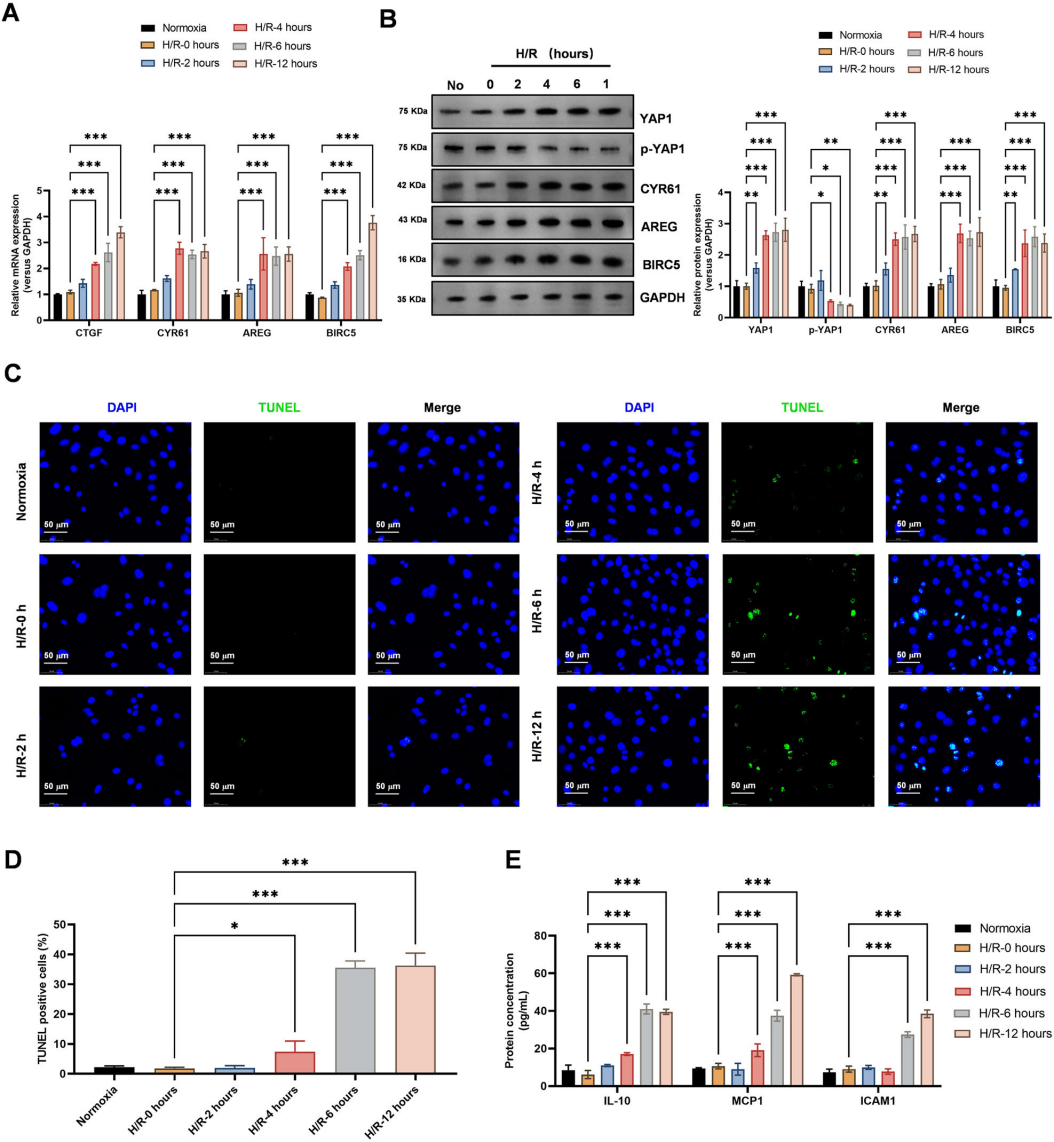
Hypoxia-reoxygenation significantly activates Hippo-YAP1 signaling pathway activity and cellular damage in lung epithelial cells. **A** Realtime PCR revealed a gradual increase in the expression levels of Hippo-YAP signaling pathway molecules (CTGF, CYR61, AREG, and BIRC5) in cells subjected to hypoxia-reoxygenation (0, 2, 4, 6, 12 h). Data represent mean ± SEM (*N* = 3), ***P < 0.001, compared with indicated group by two-way ANOVA along with Tukey’s post hoc test. **B** Western Blot analysis showed a gradual increase in the protein expression levels of Hippo-YAP signaling pathway molecules (YAP1, phosphorylated YAP1, CYR61, AREG, and BIRC5) in cells subjected to hypoxia-reoxygenation (0, 2, 4, 6, 12 h). Data represent mean ± SEM (*N* = 3), *P < 0.05, **P < 0.01, ***P < 0.001, compared with indicated group by two-way ANOVA along with Tukey’s post hoc test. **C, D** TUNEL assays demonstrated a gradual increase in apoptosis levels in lung epithelial cells subjected to hypoxia-reoxygenation (0, 2, 4, 6, 12 h). Data represent mean ± SEM (*N* = 3), *P < 0.05, ***P < 0.001, compared with indicated group by one-way ANOVA along with Tukey’s post hoc test. **E** ELISA experiments showed a gradual increase in the secretion of inflammatory cytokines (IL-10, MCP1, and ICAM1) in cells subjected to hypoxia-reoxygenation (0, 2, 4, 6, 12 h). Data represent mean ± SEM (*N* = 3), ***P < 0.001, compared with indicated group by two-way ANOVA along with Tukey’s post hoc test.

Firstly, we investigated the effects of hypoxia-reoxygenation (H/R) on Hippo-YAP1 signaling pathway activity and cellular damage in lung epithelial cells. Our results demonstrated that H/R treatment significantly activated the Hippo-YAP1 signaling pathway, as evidenced by a gradual increase in the expression levels of Hippo-YAP signaling pathway molecules such as CTGF, CYR61, AREG, and BIRC5 at both the mRNA and protein levels (Fig. 1A, B). Additionally, we observed a gradual increase in apoptosis levels in lung epithelial cells subjected to H/R, as demonstrated by TUNEL assays (Fig. 1C, D). Furthermore, ELISA experiments showed a gradual increase in the secretion of inflammatory cytokines such as IL-10, MCP1, and ICAM1 in cells subjected to H/R (Fig. 1E).

**Fig. 2:**
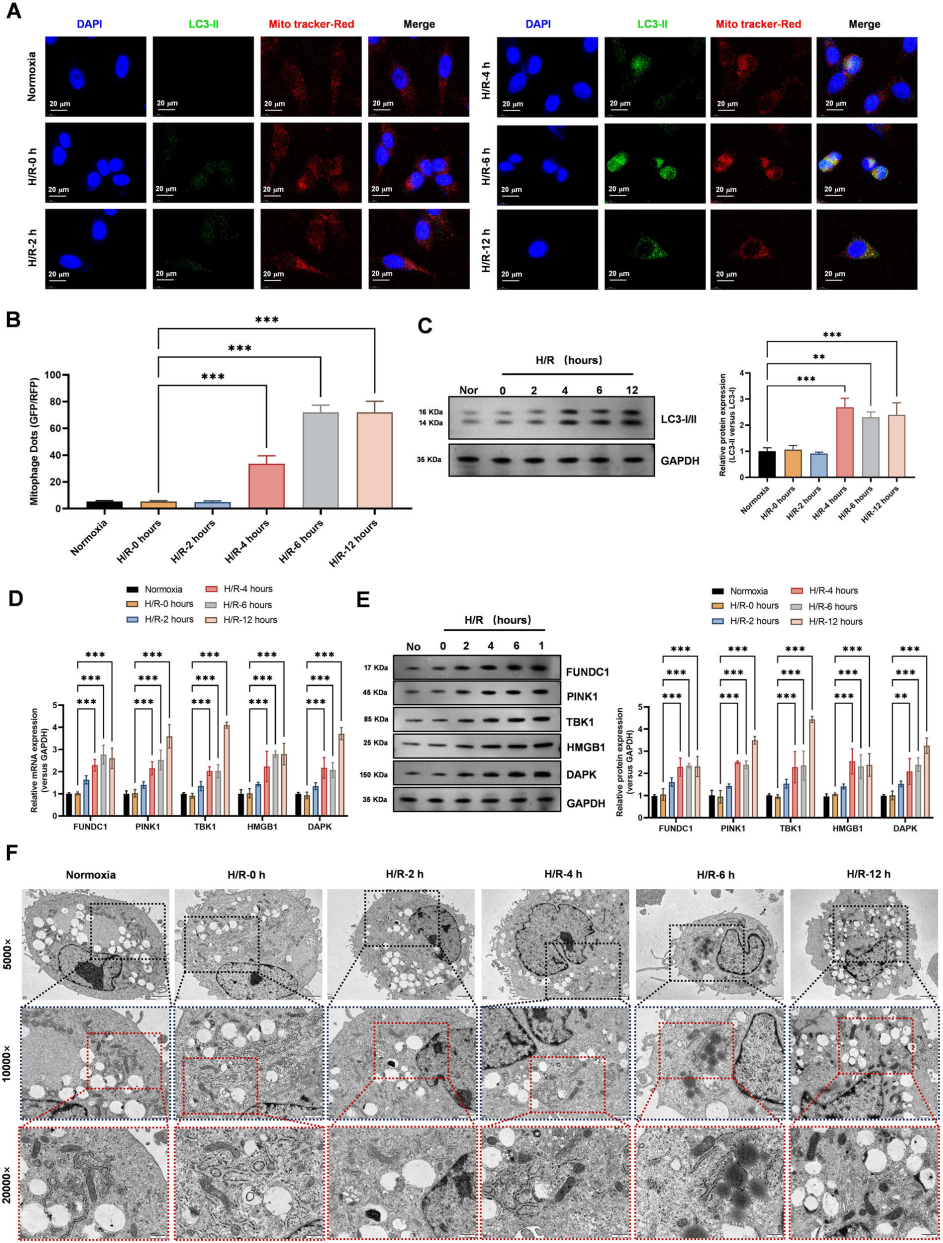
Hypoxia-reoxygenation significantly activates autophagy and mitochondrial autophagy levels in lung epithelial cells. **A, B** Immunofluorescence confocal laser scanning microscopy detected a gradual increase in mitochondrial autophagy levels in cells subjected to hypoxia-reoxygenation (0, 2, 4, 6, 12 h). Data represent mean ± SEM (*N* = 3), ***P < 0.001, compared with indicated group by one-way ANOVA along with Tukey’s post hoc test. **C** Western Blot analysis showed a gradual increase in the expression level of the autophagy protein LC3-II in cells subjected to hypoxia-reoxygenation (0, 2, 4, 6, 12 h). Data represent mean ± SEM (*N* = 3), **P < 0.01, ***P < 0.001, compared with indicated group by one-way ANOVA along with Tukey’s post hoc test. **D** Realtime PCR revealed a gradual increase in the expression levels of autophagy genes (*HMGB1*, *DAPK*) and mitochondrial autophagy genes (*FUNDC1*, *PINK1*, *TBK1*) in cells subjected to hypoxia-reoxygenation (0, 2, 4, 6, 12 h). Data represent mean ± SEM (*N* = 3), ***P < 0.001, compared with indicated group by two-way ANOVA along with Tukey’s post hoc test. **E** Western Blot analysis showed a gradual increase in the protein expression levels of autophagy genes (*HMGB1*, *DAPK*) and mitochondrial autophagy genes (*FUNDC1*, *PINK1*, *TBK1*) in cells subjected to hypoxia-reoxygenation (0, 2, 4, 6, 12 h). Data represent mean ± SEM (*N* = 3), **P < 0.01, ***P < 0.001, compared with indicated group by two-way ANOVA along with Tukey’s post hoc test. **F** Transmission electron microscopy observations revealed a gradual increase in the morphological phenotypes of mitochondrial autophagy in cells subjected to hypoxia-reoxygenation (0, 2, 4, 6, 12 h).

Next, we examined the effects of H/R on autophagy and mitochondrial autophagy levels in lung epithelial cells. Our results showed that H/R treatment significantly activated autophagy and mitochondrial autophagy, as evidenced by a gradual increase in mitochondrial autophagy levels detected by immunofluorescence confocal laser scanning microscopy (Fig. 2A, B). Additionally, we observed a gradual increase in the expression level of the autophagy protein LC3-II in cells subjected to H/R (Fig. 2C). Furthermore, Realtime PCR and Western Blot analysis revealed a gradual increase in the expression levels of autophagy genes (*HMGB1*, *DAPK*) and mitochondrial autophagy genes (*FUNDC1*, *PINK1*, *TBK1*) in cells subjected to H/R (Fig. 2D, E). Finally, transmission electron microscopy observations confirmed a gradual increase in the morphological phenotypes of mitochondrial autophagy in cells subjected to H/R (Fig. 2F).

### Lung transplant I/R injury activates autophagy and mitophagy

**Fig. 3:**
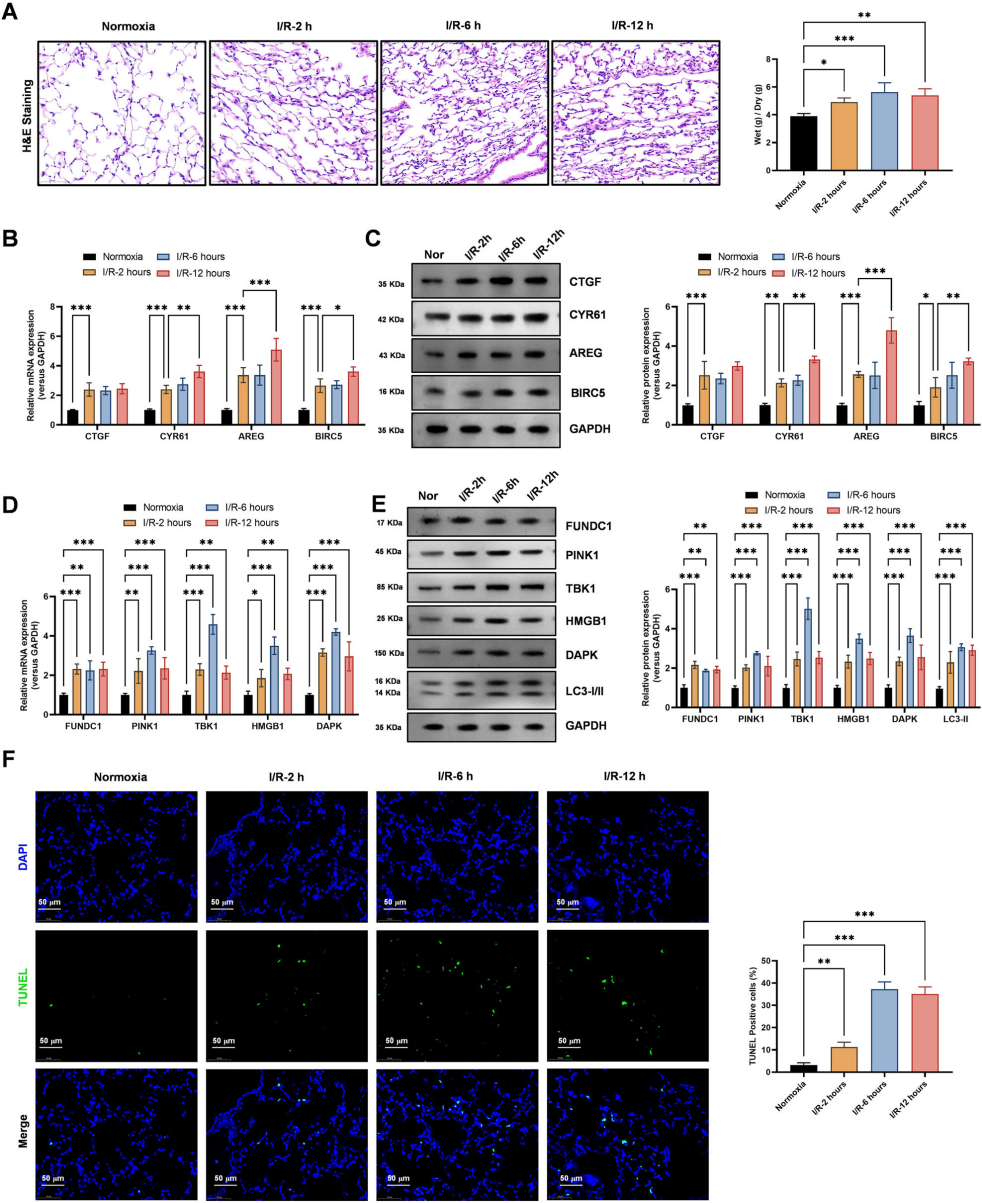
Lung transplant ischemia-reperfusion significantly activates autophagy and mitochondrial autophagy levels and damage levels in lung tissue. **A** HE staining experiments assessed gradually increasing pathological damage and lung edema in lung tissue at different time points of ischemia-reperfusion (2, 6, 12 h). Data represent mean ± SEM (*N* = 3), *P < 0.05, **P < 0.01, ***P < 0.001, compared with indicated group by one-way ANOVA along with Tukey’s post hoc test. **B** Realtime PCR revealed a gradual increase in the expression levels of Hippo-YAP signaling pathway molecules (CTGF, CYR61, AREG, and BIRC5) in cells subjected to ischemia-reperfusion (2, 6, 12 h). Data represent mean ± SEM (*N* = 3), *P < 0.05, **P < 0.01, ***P < 0.001, compared with indicated group by two-way ANOVA along with Tukey’s post hoc test. **C** Western Blot analysis showed a gradual increase in the protein expression levels of Hippo-YAP signaling pathway molecules (CTGF, CYR61, AREG, and BIRC5) in cells subjected to ischemia-reperfusion (2, 6, 12 h). Data represent mean ± SEM (*N* = 3), *P < 0.05, **P < 0.01, ***P < 0.001, compared with indicated group by two-way ANOVA along with Tukey’s post hoc test. **D** Realtime PCR revealed a gradual increase in the expression levels of autophagy genes (*HMGB1*, *DAPK*, and *LC3-II*) and mitochondrial autophagy genes (*FUNDC1*, *PINK1*, *TBK1*) in cells subjected to ischemia-reperfusion (2, 6, 12 h). Data represent mean ± SEM (*N* = 3), *P < 0.05, **P < 0.01, ***P < 0.001, compared with indicated group by two-way ANOVA along with Tukey’s post hoc test. **E** Western Blot analysis showed a gradual increase in the protein expression levels of autophagy genes (*HMGB1*, *DAPK*, and *LC3-II*) and mitochondrial autophagy genes (*FUNDC1*, *PINK1*, *TBK1*) in cells subjected to ischemia-reperfusion (2, 6, 12 h). Data represent mean ± SEM (*N* = 3), **P < 0.01, ***P < 0.001, compared with indicated group by two-way ANOVA along with Tukey’s post hoc test. **F** TUNEL assays demonstrated a gradual increase in apoptosis levels in lung epithelial cells subjected to ischemia-reperfusion (2, 6, 12 h). Data represent mean ± SEM (*N* = 3), **P < 0.01, ***P < 0.001, compared with indicated group by two-way ANOVA along with Tukey’s post hoc test.

HE staining experiments revealed a gradual increase in pathological damage and lung edema in lung tissue subjected to I/R injury at different time points (2, 6, 12 h, Fig. 3A). These findings suggest that I/R injury results in significant tissue damage and inflammation, which are hallmarks of this pathophysiological process. Realtime PCR (Fig. 3B) and Western blot (Fig. 3C) analysis showed a gradual increase in the expression levels of Hippo-YAP signaling pathway molecules (CTGF, CYR61, AREG, and BIRC5) in cells subjected to I/R injury (2, 6, 12 h). These results indicate that the Hippo-YAP signaling pathway is activated in response to I/R injury, potentially contributing to the tissue repair and regenerative responses observed in this setting. Realtime PCR (Fig. 3D) and Western blot (Fig. 3E) analysis also revealed a gradual increase in the expression levels of autophagy genes (*HMGB1*, *DAPK*, and *LC3-II*) and mitophagy genes (*FUNDC1*, *PINK1*, *TBK1*) in cells subjected to I/R injury (2, 6, 12 h). These findings suggest that autophagy and mitophagy are activated in response to I/R injury, potentially serving as protective mechanisms to clear damaged organelles and proteins and maintain cellular homeostasis. TUNEL assays (Fig. 3F) demonstrated a gradual increase in apoptosis levels in lung epithelial cells subjected to I/R injury (2, 6, 12 h). These results indicate that I/R injury results in significant cell death, which may contribute to the tissue damage and impaired graft function observed in this setting.

### YAP1 knockdown reduces autophagy and mitophagy in lung epithelial cells subjected to H/R

**Fig. 4:**
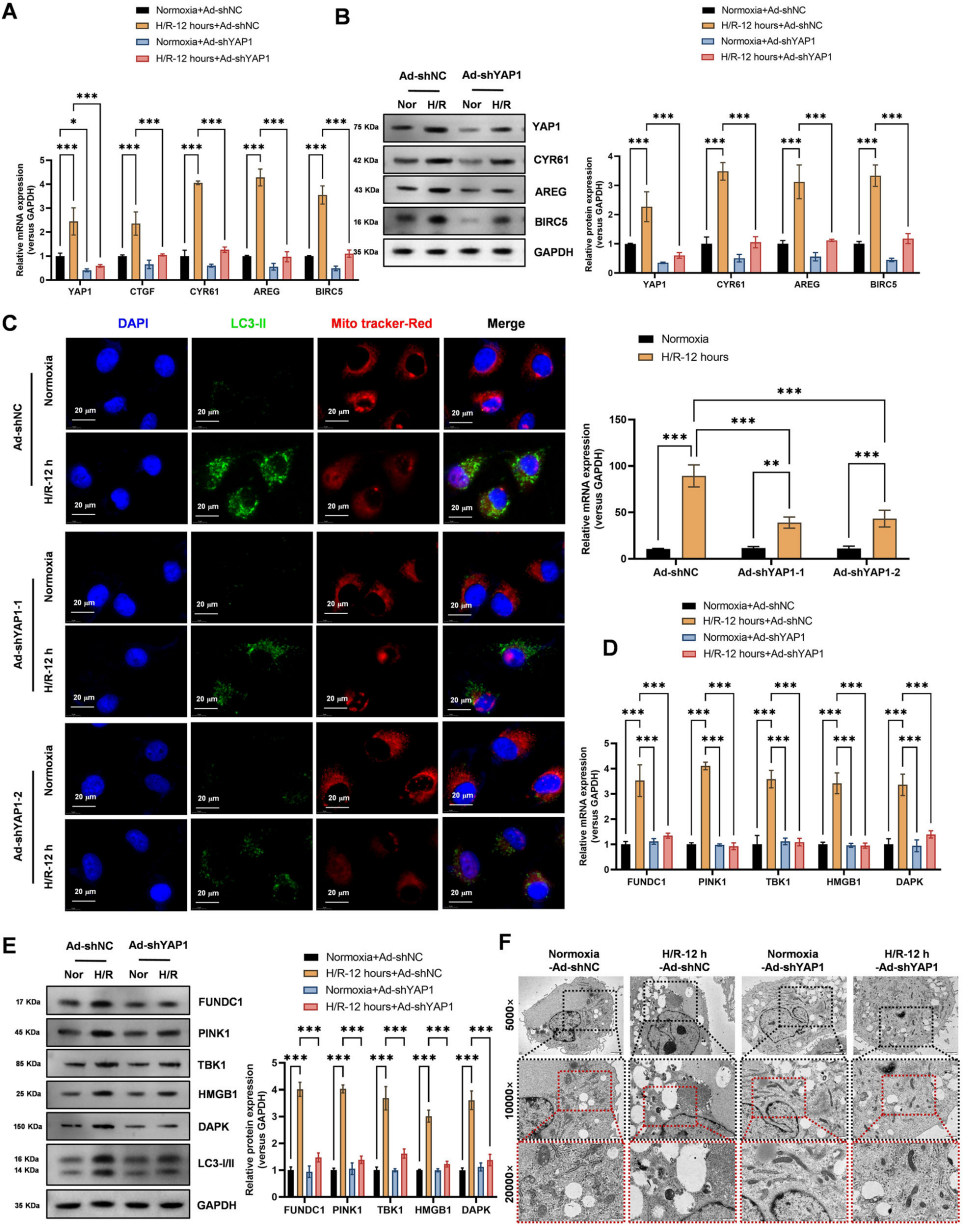
YAP1 knockdown significantly reduces autophagy and mitochondrial autophagy levels activated by hypoxia-reoxygenation in lung epithelial cells. **A** Realtime PCR revealed that YAP1 knockdown significantly inhibited the increased expression levels of Hippo-YAP signaling pathway molecules (CTGF, CYR61, AREG, and BIRC5) in cells subjected to hypoxia-reoxygenation (12 h). Data represent mean ± SEM (*N* = 3), *P < 0.05, ***P < 0.001, compared with indicated group by two-way ANOVA along with Tukey’s post hoc test. **B** Western Blot analysis showed that YAP1 knockdown significantly inhibited the increased protein expression levels of Hippo-YAP signaling pathway molecules (YAP1, CYR61, AREG, and BIRC5) in cells subjected to hypoxia-reoxygenation (12 h). Data represent mean ± SEM (*N* = 3), ***P < 0.001, compared with indicated group by two-way ANOVA along with Tukey’s post hoc test. **C** Immunofluorescence confocal laser scanning microscopy detected that YAP1 knockdown with two independent shRNAs targeting YAP1 (shYAP1-1 and shYAP1-2) significantly inhibited the increased mitochondrial autophagy levels in cells subjected to hypoxia-reoxygenation (12 h). Data represent mean ± SEM (*N* = 3), **P < 0.01, ***P < 0.001, compared with indicated group by two-way ANOVA along with Tukey’s post hoc test. **D** Realtime PCR revealed that YAP1 knockdown significantly inhibited the increased expression levels of autophagy genes (HMGB1, *DAPK*) and mitochondrial autophagy genes (*FUNDC1*, *PINK1*, *TBK1*) in cells subjected to hypoxia-reoxygenation (12 h). Data represent mean ± SEM (*N* = 3), ***P < 0.001, compared with indicated group by two-way ANOVA along with Tukey’s post hoc test. **E** Western Blot analysis showed that YAP1 knockdown significantly inhibited the increased protein expression levels of autophagy genes (*HMGB1*, *DAPK*, and *LC3-II*) and mitochondrial autophagy genes (*FUNDC1*, *PINK1*, *TBK1*) in cells subjected to hypoxia-reoxygenation (12 h). **F** Transmission electron microscopy observations revealed that YAP1 knockdown significantly inhibited the morphological phenotypes of mitochondrial autophagy induced by hypoxia-reoxygenation (12 h) in cells. Data represent mean ± SEM (*N* = 3), ***P < 0.001, compared with indicated group by two-way ANOVA along with Tukey’s post hoc test.

Realtime PCR (Fig. 4A) and Western blot (Fig. 4B) analysis showed that YAP1 knockdown significantly inhibited the increased expression levels of Hippo-YAP signaling pathway molecules (CTGF, CYR61, AREG, and BIRC5) in cells subjected to H/R (12 h). These results suggest that YAP1 plays a critical role in the activation of Hippo-YAP signaling in response to H/R injury. Immunofluorescence confocal laser scanning microscopy (Fig. 4C) detected that YAP1 knockdown with two independent shRNAs targeting YAP1 (shYAP1-1 and shYAP1-2) significantly inhibited the increased mitophagy levels in cells subjected to H/R (12 h). These findings indicate that YAP1 is required for the activation of mitophagy in response to H/R injury. Realtime PCR (Fig. 4D) and Western blot (Fig. 4E) analysis revealed that YAP1 knockdown significantly inhibited the increased expression levels of autophagy genes (*HMGB1*, *DAPK*) and mitophagy genes (*FUNDC1*, *PINK1*, *TBK1*) in cells subjected to H/R (12 h). These results suggest that YAP1 is a key regulator of autophagy and mitophagy in lung epithelial cells subjected to H/R injury. Transmission electron microscopy (Fig. 4F) observations revealed that YAP1 knockdown significantly inhibited the morphological phenotypes of mitophagy induced by H/R (12 h) in cells. These findings provide further evidence that YAP1 is required for the activation of mitophagy in response to H/R injury.

### YAP1 knockdown reduces autophagy, mitophagy, and tissue damage in lung transplant I/R injury model

**Fig. 5:**
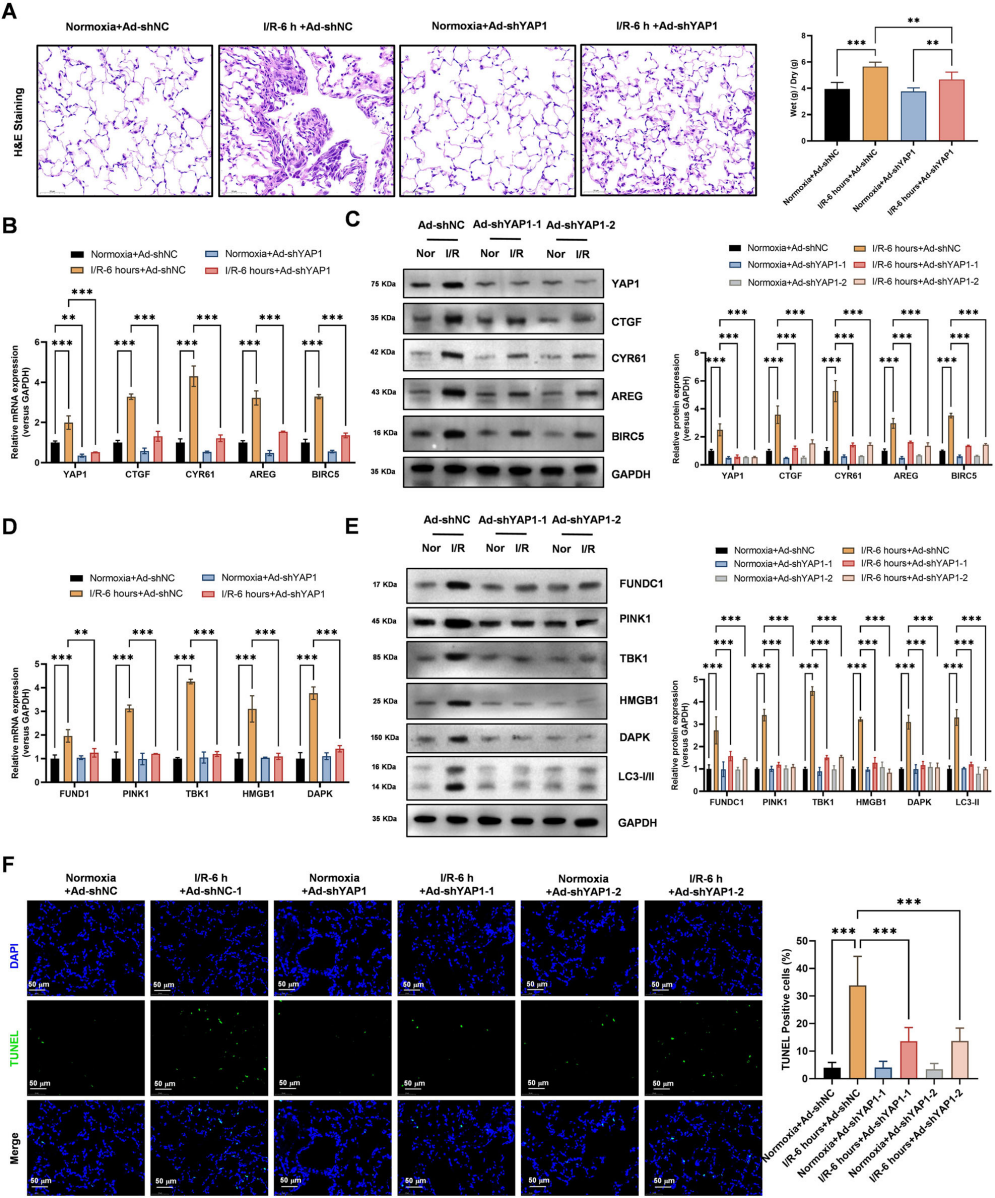
YAP1 knockdown significantly reduces autophagy and mitochondrial autophagy levels and damage levels activated by lung transplant ischemia-reperfusion. **A** HE staining experiments assessed that YAP1 knockdown significantly inhibited the increased pathological damage and lung edema in lung tissue subjected to ischemia-reperfusion (6 h). Data represent mean ± SEM (*N* = 3), **P < 0.01, ***P < 0.001, compared with indicated group by one-way ANOVA along with Tukey’s post hoc test. **B** Realtime PCR revealed that YAP1 knockdown significantly inhibited the increased expression levels of Hippo-YAP signaling pathway molecules (CTGF, CYR61, AREG, and BIRC5) in cells subjected to ischemia-reperfusion (6 h). Data represent mean ± SEM (*N* = 3), **P < 0.01, ***P < 0.001, compared with indicated group by two-way ANOVA along with Tukey’s post hoc test. **C** Western Blot analysis showed that YAP1 knockdown with two independent shRNAs targeting YAP1 (shYAP1-1 and shYAP1-2) significantly inhibited the increased protein expression levels of Hippo-YAP signaling pathway molecules (CTGF, CYR61, AREG, and BIRC5) in cells subjected to ischemia-reperfusion (6 h). Data represent mean ± SEM (*N* = 3), ***P < 0.001, compared with indicated group by two-way ANOVA along with Tukey’s post hoc test. **D** Realtime PCR revealed that YAP1 knockdown significantly inhibited the increased expression levels of autophagy genes (*HMGB1*, *DAPK*, and *LC3-II*) and mitochondrial autophagy genes (*FUNDC1*, *PINK1*, *TBK1*) in cells subjected to ischemia-reperfusion (6 h). Data represent mean ± SEM (*N* = 3), **P < 0.01, ***P < 0.001, compared with indicated group by two-way ANOVA along with Tukey’s post hoc test. **E** Western Blot analysis showed that YAP1 knockdown with two independent shRNAs targeting YAP1 (shYAP1-1 and shYAP1-2) significantly inhibited the increased protein expression levels of autophagy genes (*HMGB1*, *DAPK*, and *LC3-II*) and mitochondrial autophagy genes (*FUNDC1*, *PINK1*, *TBK1*) in cells subjected to ischemia-reperfusion (6 h). Data represent mean ± SEM (*N* = 3), **P < 0.01, ***P < 0.001, compared with indicated group by two-way ANOVA along with Tukey’s post hoc test. **F** TUNEL assays demonstrated that YAP1 knockdown with two independent shRNAs targeting YAP1 (shYAP1-1 and shYAP1-2) significantly inhibited the increased apoptosis levels in lung epithelial cells subjected to ischemia-reperfusion (6 h). Data represent mean ± SEM (*N* = 3), **P < 0.01, ***P < 0.001, compared with indicated group by one-way ANOVA along with Tukey’s post hoc test.

HE staining experiments (Fig. 5A) assessed that YAP1 knockdown significantly inhibited the increased pathological damage and lung edema in lung tissue subjected to I/R (6 h). These results suggest that YAP1 knockdown may have protective effects against I/R-induced tissue damage. Realtime PCR (Fig. 5B) and Western blot (Fig. 5D) analysis showed that YAP1 knockdown significantly inhibited the increased expression levels of Hippo-YAP signaling pathway molecules (CTGF, CYR61, AREG, and BIRC5) in cells subjected to I/R (6 h). These findings indicate that YAP1 is a critical regulator of Hippo-YAP signaling in response to I/R injury. Realtime PCR (Fig. 5D) and Western blot (Fig. 5E) analysis revealed that YAP1 knockdown significantly inhibited the increased expression levels of autophagy genes (*HMGB1*, *DAPK*, and *LC3-II*) and mitophagy genes (*FUNDC1*, *PINK1*, *TBK1*) in cells subjected to I/R (6 h). These results suggest that YAP1 is a key regulator of autophagy and mitophagy in lung tissue subjected to I/R injury. TUNEL assays (Fig. 5F) demonstrated that YAP1 knockdown significantly inhibited the increased apoptosis levels in lung epithelial cells subjected to I/R (6 h). These findings indicate that YAP1 knockdown may have protective effects against I/R-induced cell death, potentially contributing to the reduction in tissue damage observed in this setting.

### OGT-mediated O-GlcNAc modification of YAP1 protein enhances its binding to hif1α and activates the expression of downstream hypoxia-responsive molecules

**Fig. 6:**
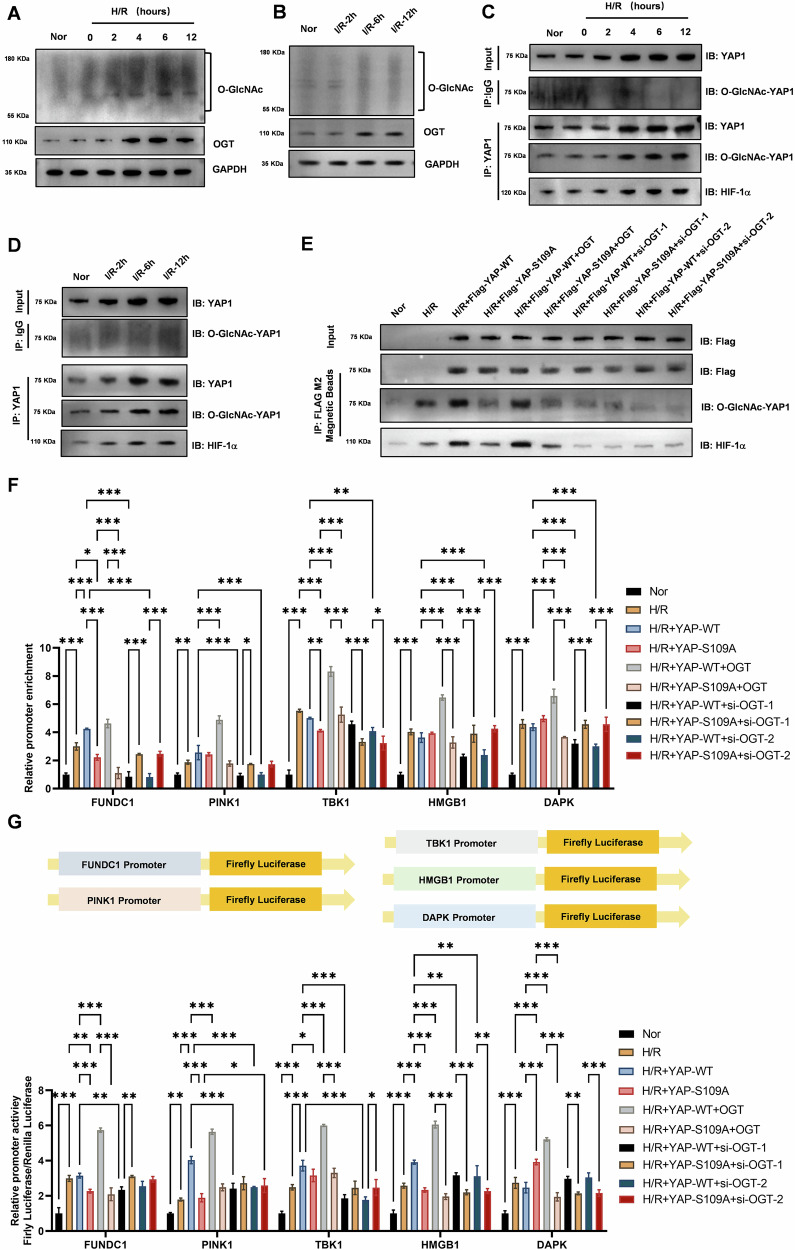
OGT-Mediated O-GlcNAc Modification of YAP1 Protein Enhances Its Binding to HIF1α and Activates the Expression of Downstream Hypoxia-Responsive Molecules. **A** Western blot analysis revealed that hypoxia-reoxygenation (0, 2, 4, 6, 12 h) treatment gradually increased the O-GlcNAc modification level of total cellular proteins and the expression of the key modifying enzyme OGT. **B** Western blot analysis showed that ischemia-reperfusion (2, 6, 12 h) gradually increased the O-GlcNAc modification level of total lung tissue proteins and the expression of OGT. **C** YAP1 protein immunoprecipitation experiments demonstrated that hypoxia-reoxygenation (0, 2, 4, 6, 12 h) treatment gradually increased the O-GlcNAc modification level of cellular YAP1 protein. **D** YAP1 protein immunoprecipitation experiments revealed that ischemia-reperfusion (2, 6, 12 h) gradually increased the O-GlcNAc modification level of YAP1 protein in lung tissue. **E** Flag-YAP1 protein immunoprecipitation with M2 Magnetic Beads experiments revealed that overexpression of wild-type OGT significantly increased the O-GlcNAc modification level of wild-type Flag-YAP1 protein and its binding to the HIF1A transcription factor in alveolar epithelial cells under hypoxia-reoxygenation (12 h). Meanwhile, overexpression of OGT with a serine mutation at the O-GlcNAc modification site had no such effect. Additionally, OGT knockdown significantly inhibited the O-GlcNAc modification level of wild-type Flag-YAP1 protein and its binding to the HIF1A transcription factor in alveolar epithelial cells under hypoxia-reoxygenation (12 h). **F** Chromatin immunoprecipitation (ChIP) experiments for HIF1α found that overexpression of wild-type OGT significantly increased the binding level of the HIF1A transcription factor to the promoters of autophagy genes (*HMGB1*, *DAPK*, and *LC3-II*) and mitophagy genes (*FUNDC1*, *PINK1*, *TBK1*) in alveolar epithelial cells under hypoxia-reoxygenation (12 h). In contrast, overexpression of OGT with a serine mutation at the O-GlcNAc modification site had no such effect. Moreover, OGT knockdown significantly inhibited the binding level of the HIF1A transcription factor to the promoters of these autophagy and mitophagy genes in alveolar epithelial cells under hypoxia-reoxygenation (12 h). Data represent mean ± SEM (*N* = 3), *P < 0.05, **P < 0.01, ***P < 0.001, compared with indicated group by two-way ANOVA along with Tukey’s post hoc test. **G** Dual luciferase reporter experiments demonstrated that overexpression of wild-type OGT significantly increased the promoter activity of autophagy genes (*HMGB1*, *DAPK*, and *LC3-II*) and mitophagy genes (*FUNDC1*, *PINK1*, *TBK1*) in alveolar epithelial cells under hypoxia-reoxygenation (12 h). Conversely, overexpression of OGT with a serine mutation at the O-GlcNAc modification site had no such effect. Additionally, OGT knockdown significantly inhibited the promoter activity of these autophagy and mitophagy genes in alveolar epithelial cells under hypoxia-reoxygenation (12 h). Data represent mean ± SEM (*N* = 3), *P < 0.05, **P < 0.01, ***P < 0.001, compared with indicated group by two-way ANOVA along with Tukey’s post hoc test.

To investigate the dynamics of O-GlcNAcylation during hypoxia-reoxygenation (H/R) and ischemia-reperfusion (IR), we performed western blot analysis. Our results showed that both H/R and IR treatments gradually increased the O-GlcNAc modification level of total cellular and lung tissue proteins, respectively (Figs. 6A and 6B). This increase was accompanied by a gradual upregulation of the key modifying enzyme O-GlcNAc transferase (OGT), suggesting that O-GlcNAcylation is actively regulated during these stress conditions. To specifically examine the O-GlcNAcylation of YAP1, we conducted immunoprecipitation experiments. Our results demonstrated that both H/R and IR treatments gradually increased the O-GlcNAc modification level of cellular and lung tissue YAP1 protein, respectively (Fig. 6C, D). These findings indicate that YAP1 undergoes O-GlcNAcylation in response to stress conditions, potentially altering its function and interactions. To further investigate the role of O-GlcNAcylation in YAP1 function, we manipulated the expression of OGT. Flag-YAP1 protein immunoprecipitation with M2 Magnetic Beads experiments showed that overexpression of wild-type OGT significantly increased the O-GlcNAc modification level of wild-type Flag-YAP1 protein and its binding to the HIF1α transcription factor in alveolar epithelial cells under H/R conditions (Fig. 6E). In contrast, overexpression of OGT with a serine mutation at the O-GlcNAc modification site had no such effect. Additionally, OGT knockdown significantly inhibited the O-GlcNAc modification level of wild-type Flag-YAP1 protein and its binding to HIF1α under H/R conditions. These results suggest that O-GlcNAcylation of YAP1 is required for its interaction with HIF1α. To determine the functional consequences of the YAP1-HIF1α interaction, we performed ChIP experiments. Our results showed that overexpression of wild-type OGT significantly increased the binding level of the HIF1α transcription factor to the promoters of autophagy genes (*HMGB1*, *DAPK*, and *LC3-II*) and mitophagy genes (*FUNDC1*, *PINK1*, *TBK1*) in alveolar epithelial cells under H/R conditions (Fig. 6F). In contrast, overexpression of OGT with a serine mutation at the O-GlcNAc modification site had no such effect. Moreover, OGT knockdown significantly inhibited the binding level of HIF1α to the promoters of these autophagy and mitophagy genes. These findings suggest that O-GlcNAcylation of YAP1 enhances its ability to recruit HIF1α to the promoters of autophagy and mitophagy genes, thereby activating their expression. To directly assess the promoter activity of autophagy and mitophagy genes, we performed dual luciferase reporter experiments. Our results demonstrated that overexpression of wild-type OGT significantly increased the promoter activity of autophagy genes (*HMGB1*, *DAPK*, and *LC3-II*) and mitophagy genes (*FUNDC1*, *PINK1*, *TBK1*) in alveolar epithelial cells under H/R conditions (Fig. 6G). Conversely, overexpression of OGT with a serine mutation at the O-GlcNAc modification site had no such effect. Additionally, OGT knockdown significantly inhibited the promoter activity of these autophagy and mitophagy genes. These results further confirm that O-GlcNAcylation of YAP1 is required for the activation of autophagy and mitophagy gene expression.

### Knockdown of the O-GlcNAc modification key enzyme OGT significantly reduces the levels of autophagy and mitophagy activated by hypoxia-reoxygenation in lung epithelial cells

**Fig. 7:**
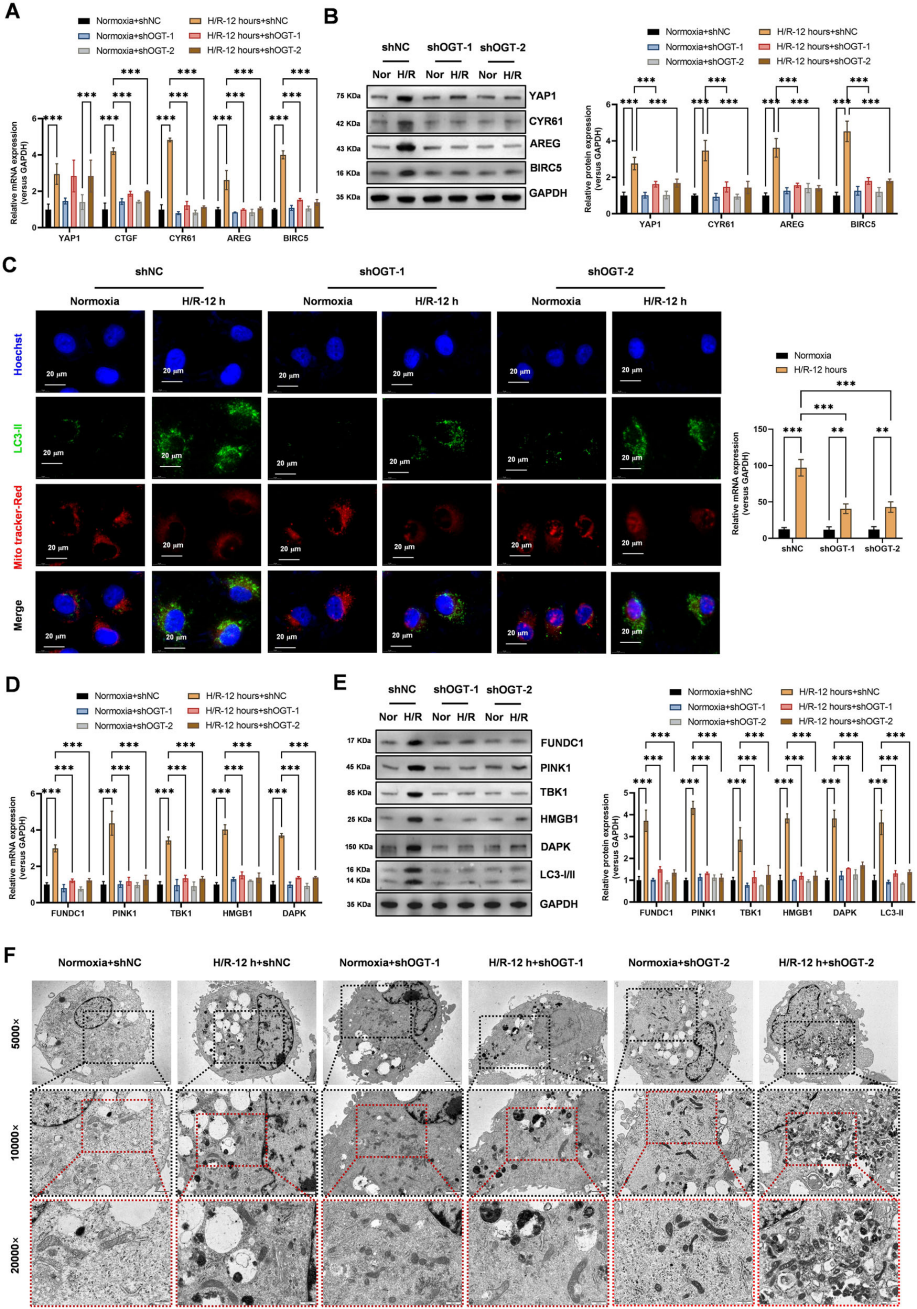
Knockdown of the O-GlcNAc Modification Key Enzyme OGT Significantly Reduces the Levels of Autophagy and Mitophagy Activated by Hypoxia-Reoxygenation in Lung Epithelial Cells. **A** Real-time qPCR analysis revealed that OGT knockdown significantly inhibited the increased expression levels of Hippo-YAP signaling pathway molecules (CTGF, CYR61, AREG, and BIRC5) in cells treated with hypoxia-reoxygenation (12 h). Data represent mean ± SEM (*N* = 3), *P < 0.05, **P < 0.01, ***P < 0.001, compared with indicated group by two-way ANOVA along with Tukey’s post hoc test. **B** Western blot analysis showed that OGT knockdown significantly inhibited the increased protein expression levels of Hippo-YAP signaling pathway molecules (YAP1, CYR61, AREG, and BIRC5) in cells treated with hypoxia-reoxygenation (12 h). Data represent mean ± SEM (*N* = 3), *P < 0.05, **P < 0.01, ***P < 0.001, compared with indicated group by two-way ANOVA along with Tukey’s post hoc test. **C** Immunofluorescence confocal laser scanning microscopy experiments demonstrated that OGT knockdown significantly inhibited the increased level of mitophagy in cells treated with hypoxia-reoxygenation (12 h). Data represent mean ± SEM (*N* = 3), *P < 0.05, **P < 0.01, ***P < 0.001, compared with indicated group by two-way ANOVA along with Tukey’s post hoc test. **D** Real-time qPCR analysis found that OGT knockdown significantly inhibited the increased expression levels of autophagy genes (*HMGB1*, *DAPK*) and mitophagy genes (*FUNDC1*, *PINK1*, *TBK1*) in cells treated with hypoxia-reoxygenation (12 h). Data represent mean ± SEM (*N* = 3), *P < 0.05, **P < 0.01, ***P < 0.001, compared with indicated group by two-way ANOVA along with Tukey’s post hoc test. **E** Western blot analysis revealed that OGT knockdown significantly inhibited the increased protein expression levels of autophagy genes (*HMGB1*, *DAPK*, and *LC3-II*) and mitophagy genes (*FUNDC1*, *PINK1*, *TBK1*) in cells treated with hypoxia-reoxygenation (12 h). Data represent mean ± SEM (*N* = 3), *P < 0.05, **P < 0.01, ***P < 0.001, compared with indicated group by two-way ANOVA along with Tukey’s post hoc test. **F** Transmission electron microscopy observations showed that OGT knockdown significantly inhibited the morphological phenotypes of mitophagy induced by hypoxia-reoxygenation (12 h) treatment in cells.

To assess the effect of OGT knockdown on the Hippo-YAP signaling pathway, we performed real-time qPCR and western blot analysis. Our results showed that OGT knockdown significantly inhibited the increased expression levels of Hippo-YAP signaling pathway molecules (CTGF, CYR61, AREG, and BIRC5) in cells treated with H/R (Fig. 7A, B). These findings suggest that OGT is required for the activation of the Hippo-YAP signaling pathway during H/R. To visualize the effect of OGT knockdown on mitophagy, we performed immunofluorescence confocal laser scanning microscopy experiments. Our results demonstrated that OGT knockdown significantly inhibited the increased level of mitophagy in cells treated with H/R (Fig. 7C). This finding further supports the role of OGT in regulating mitophagy during H/R. To directly assess the effect of OGT knockdown on autophagy and mitophagy gene expression, we performed real-time qPCR and western blot analysis. Our results showed that OGT knockdown significantly inhibited the increased expression levels of autophagy genes (*HMGB1*, *DAPK*) and mitophagy genes (*FUNDC1*, *PINK1*, *TBK1*) in cells treated with H/R (Fig. 7D, E). These findings suggest that OGT is required for the activation of autophagy and mitophagy gene expression during H/R. To further visualize the effect of OGT knockdown on mitophagy, we performed transmission electron microscopy observations. Our results demonstrated that OGT knockdown significantly inhibited the morphological phenotypes of mitophagy induced by H/R treatment in cells (Fig. 7F). This finding provides additional evidence for the role of OGT in regulating mitophagy during H/R.

### Knockdown of the O-GlcNAc modification key enzyme OGT significantly reduces the levels of autophagy, mitophagy, and damage activated by ischemia-reperfusion in lung tissue during lung

**Fig. 8:**
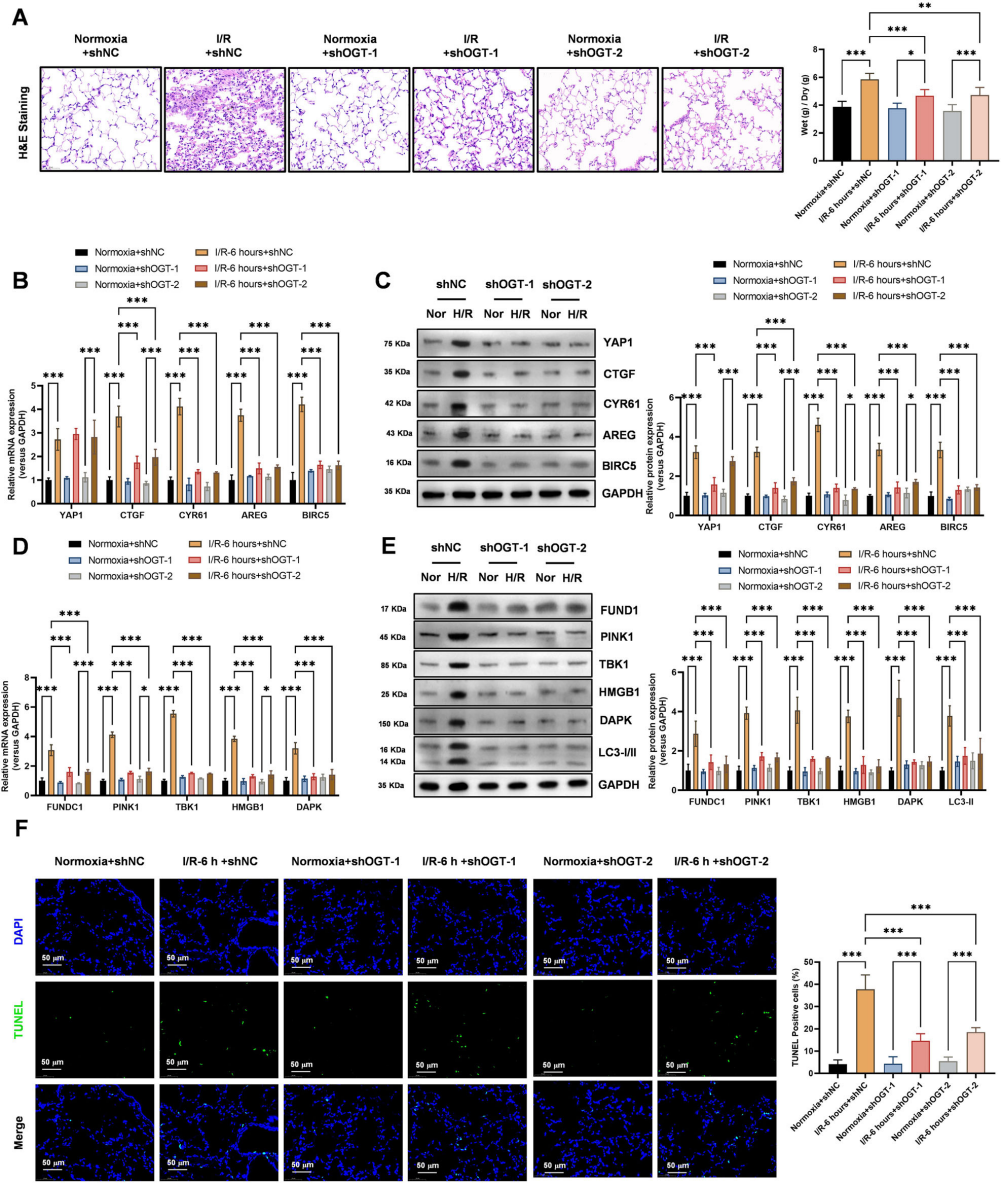
Knockdown of the O-GlcNAc Modification Key Enzyme OGT Significantly Reduces the Levels of Autophagy, Mitophagy, and Damage Activated by Ischemia-Reperfusion in Lung Tissue During Lung Transplantation. **A** HE staining experiments assessed that OGT knockdown significantly inhibited the increased pathological damage and lung edema in lung tissue subjected to ischemia-reperfusion (6 h) during lung transplantation. Data represent mean ± SEM (*N* = 3), *P < 0.05, **P < 0.01, ***P < 0.001, compared with indicated group by one-way ANOVA along with Tukey’s post hoc test. **B** Real-time qPCR analysis found that OGT knockdown significantly inhibited the increased expression levels of Hippo-YAP signaling pathway molecules (CTGF, CYR61, AREG, and BIRC5) in cells subjected to ischemia-reperfusion (6 h) during lung transplantation. Data represent mean ± SEM (*N* = 3), ***P < 0.001, compared with indicated group by two-way ANOVA along with Tukey’s post hoc test. **C** Western blot analysis showed that OGT knockdown significantly inhibited the increased protein expression levels of Hippo-YAP signaling pathway molecules (CTGF, CYR61, AREG, and BIRC5) in cells subjected to ischemia-reperfusion (6 h) during lung transplantation. Data represent mean ± SEM (*N* = 3), *P < 0.05, ***P < 0.001, compared with indicated group by two-way ANOVA along with Tukey’s post hoc test. **D** Real-time qPCR analysis revealed that OGT knockdown significantly inhibited the increased expression levels of autophagy genes (*HMGB1*, *DAPK*, and *LC3-II*) and mitophagy genes (*FUNDC1*, *PINK1*, *TBK1*) in cells subjected to ischemia-reperfusion (6 h) during lung transplantation. Data represent mean ± SEM (*N* = 3), *P < 0.05, ***P < 0.001, compared with indicated group by two-way ANOVA along with Tukey’s post hoc test. **E** Western blot analysis demonstrated that OGT knockdown significantly inhibited the increased protein expression levels of autophagy genes (*HMGB1*, *DAPK*, and *LC3-II*) and mitophagy genes (*FUNDC1*, *PINK1*, *TBK1*) in cells subjected to ischemia-reperfusion (6 h) during lung transplantation. Data represent mean ± SEM (*N* = 3), ***P < 0.001, compared with indicated group by two-way ANOVA along with Tukey’s post hoc test. **F** TUNEL experiments found that OGT knockdown significantly inhibited the increased level of apoptosis in lung epithelial cells subjected to ischemia-reperfusion (6 h) during lung transplantation. Data represent mean ± SEM (*N* = 3), ***P < 0.001, compared with indicated group by one-way ANOVA along with Tukey’s post hoc test.

To assess the role of O-GlcNAcylation in IR injury, we knocked down the O-GlcNAc modification key enzyme OGT with two adenovirus mediated expression of shRNAs targeting OGT in a lung transplantation model. Histological examination using HE staining revealed that OGT knockdown significantly inhibited the increased pathological damage and lung edema in lung tissue subjected to ischemia-reperfusion (6 h) during lung transplantation (Fig. 8A). This finding suggests that O-GlcNAcylation plays a critical role in the pathogenesis of IR injury in lung transplantation. The Hippo-YAP signaling pathway is known to play a pivotal role in cell proliferation, differentiation, and survival. To investigate the effect of OGT knockdown on this pathway, we performed real-time qPCR and Western blot analyses. Our results showed that OGT knockdown significantly inhibited the increased expression levels of Hippo-YAP signaling pathway molecules, including CTGF, CYR61, AREG, and BIRC5, in cells subjected to ischemia-reperfusion (6 h) during lung transplantation (Fig. 8B, C). These findings indicate that OGT-mediated O-GlcNAcylation regulates the Hippo-YAP signaling pathway in the context of IR injury. Autophagy and mitophagy are cellular degradation processes that play important roles in maintaining cellular homeostasis and survival. To explore the effect of OGT knockdown on these processes, we analyzed the expression levels of autophagy and mitophagy genes using real-time qPCR and Western blot analyses. Our results revealed that OGT knockdown significantly inhibited the increased expression levels of autophagy genes (*HMGB1*, *DAPK*, and *LC3-II*) and mitophagy genes (*FUNDC1*, *PINK1*, *TBK1*) in cells subjected to ischemia-reperfusion (6 h) during lung transplantation (Fig. 8D, E). These findings suggest that OGT-mediated O-GlcNAcylation promotes autophagy and mitophagy in the context of IR injury. Apoptosis is a programmed cell death process that plays a crucial role in tissue damage and repair. To investigate the effect of OGT knockdown on apoptosis, we performed TUNEL experiments. Our results showed that OGT knockdown significantly inhibited the increased level of apoptosis in lung epithelial cells subjected to ischemia-reperfusion (6 h) during lung transplantation (Fig. 8F). This finding suggests that OGT-mediated O-GlcNAcylation promotes apoptosis in the context of IR injury.

## Discussion

Lung transplantation is a lifesaving procedure for patients with end-stage lung diseases [1]. However, ischemia-reperfusion injury (IRI) remains a significant challenge, leading to primary graft dysfunction (PGD) and adversely affecting patient outcomes. In this study, we investigated the role of O-GlcNAcylation of Yes-associated protein 1 (YAP1) in promoting lung transplant IRI. Our findings reveal that O-GlcNAcylation of YAP1 enhances its binding to the hypoxia-inducible factor 1α (HIF1α) transcription factor, leading to the activation of autophagy and mitochondrial autophagy, which exacerbate lung tissue damage.

YAP1, a core transcriptional co-activator in the Hippo signaling pathway, plays a crucial role in regulating cell proliferation, migration, and survival [14]. In the context of lung transplant IRI, YAP1 has been shown to be activated, contributing to tissue damage and inflammation [23]. Our study extends these findings by demonstrating that O-GlcNAcylation of YAP1 is a critical post-translational modification that amplifies its pro-inflammatory and pro-damaging effects. O-GlcNAcylation, a dynamic and reversible post-translational modification involving the addition of a single N-acetylglucosamine (GlcNAc) moiety to serine or threonine residues of proteins, plays a pivotal role in regulating various cellular processes, including gene expression, signal transduction, and metabolic pathways [49–51]. Our results indicate that O-GlcNAcylation of YAP1 significantly enhances its activity, leading to increased binding to HIF1α and subsequent activation of downstream hypoxia-responsive molecules. HIF1α is a master transcription factor that orchestrates the cellular response to hypoxia. Under hypoxic conditions, HIF1α is stabilized and translocates to the nucleus, where it binds to hypoxia-responsive elements (HREs) in the promoters of target genes, promoting their transcription [52]. Our study shows that O-GlcNAcylation of YAP1 enhances its interaction with HIF1α, leading to the increased expression of autophagy-related genes and mitochondrial autophagy genes. Autophagy and mitochondrial autophagy are essential cellular processes involved in the degradation and recycling of damaged organelles and proteins. While autophagy is generally considered a protective mechanism against stress, excessive autophagy can lead to cell death and tissue damage. In the context of lung transplant IRI, our findings suggest that O-GlcNAcylation of YAP1 promotes excessive autophagy and mitochondrial autophagy, contributing to lung tissue damage.

The mechanism by which O-GlcNAcylation of YAP1 activates autophagy and mitochondrial autophagy involves multiple signaling pathways. First, the enhanced binding of YAP1 to HIF1α leads to the increased expression of autophagy-related genes, such as *HMGB1*, *DAPK*, and *LC3-II*. These genes encode key proteins involved in the formation and maturation of autophagosomes. Secondly, the activation of mitochondrial autophagy (mitophagy) is mediated by the increased expression of mitophagy-related genes, such as *FUNDC1*, *PINK1* and *TBK1*. PINK1 is a mitochondrial kinase that accumulates on depolarized mitochondria and recruits PARK2, an E3 ubiquitin ligase, to initiate mitophagy [24]. Our results show that O-GlcNAcylation of YAP1 significantly enhances the expression of these mitophagy-related genes, leading to increased levels of mitophagy in lung epithelial cells and tissues. The functional consequences of O-GlcNAcylation of YAP1 in lung transplant IRI are multifaceted. First, the enhanced interaction between YAP1 and HIF1α leads to the increased expression of pro-inflammatory cytokines and chemokines, such as TNF-α, IL-6, and CCL2. These inflammatory mediators exacerbate lung tissue damage by attracting immune cells and promoting their activation. Secondly, the activation of autophagy and mitochondrial autophagy leads to the degradation of essential cellular components, including mitochondria, endoplasmic reticulum, and ribosomes. This process results in impaired cellular function and increased susceptibility to cell death. In the context of lung transplant IRI, excessive autophagy and mitophagy exacerbate lung tissue damage, contributing to the development of PGD.

Our findings have important therapeutic implications for the management of lung transplant IRI. First, targeting O-GlcNAcylation of YAP1 may represent a novel strategy to mitigate lung tissue damage. Inhibitors of O-GlcNAc transferase (OGT), the enzyme responsible for catalyzing O-GlcNAcylation, have been shown to reduce tissue damage in various models of ischemia-reperfusion injury [47]. Future studies should investigate the efficacy of OGT inhibitors in lung transplant models [48]. Secondly, modulating autophagy and mitochondrial autophagy may also be a promising therapeutic approach. Autophagy inhibitors, such as chloroquine and hydroxychloroquine, have been shown to reduce tissue damage in animal models of lung transplant IRI [50]. However, these drugs have potential side effects, and their use in clinical settings requires careful consideration.

While our study provides important insights into the role of O-GlcNAcylation of YAP1 in lung transplant IRI, it also has some limitations. First, our experiments were conducted in vitro and in animal models, and the results may not fully translate to human patients. Future studies should investigate the role of O-GlcNAcylation of YAP1 in human lung transplant recipients. Secondly, our study focused on the effects of O-GlcNAcylation of YAP1 on autophagy and mitochondrial autophagy. However, other cellular processes, such as apoptosis, necrosis, and inflammation, may also be affected by this post-translational modification. Future studies should investigate the broader effects of O-GlcNAcylation of YAP1 in lung transplant IRI. Finally, our study did not investigate the potential mechanisms underlying the increased O-GlcNAcylation of YAP1 in lung transplant IRI. Future studies should investigate the upstream regulators of OGT activity and their role in modulating YAP1 O-GlcNAcylation in this context.

In conclusion, our study demonstrates that O-GlcNAcylation of YAP1 promotes lung transplant IRI via binding to HIF1α transcription factor and activating autophagy and mitochondrial autophagy. These findings provide new insights into the molecular mechanisms underlying lung transplant IRI and suggest potential therapeutic targets for mitigating this complication. Future studies should investigate the efficacy of targeting O-GlcNAcylation of YAP1 and modulating autophagy and mitochondrial autophagy in lung transplant recipients.

## Supplementary information

Original WB uncropped images

## Data Availability

All data obtained in this study are available from the corresponding author upon request.
