# Supplementary material for: O-GlcNAcylation of YAP1 promotes lung transplant ischemia-reperfusion injury via binding to HIF1α transcription factor and activating autophagy and mitophagy
Source: Cell Death Dis. 2026 Mar 15;17(1):311. doi: 10.1038/s41419-026-08548-w (PMC13039928; doi:10.1038/s41419-026-08548-w)
Supplement: Supplementary file 1 — Original WB uncropped images [file 41419_2026_8548_MOESM1_ESM.pdf]

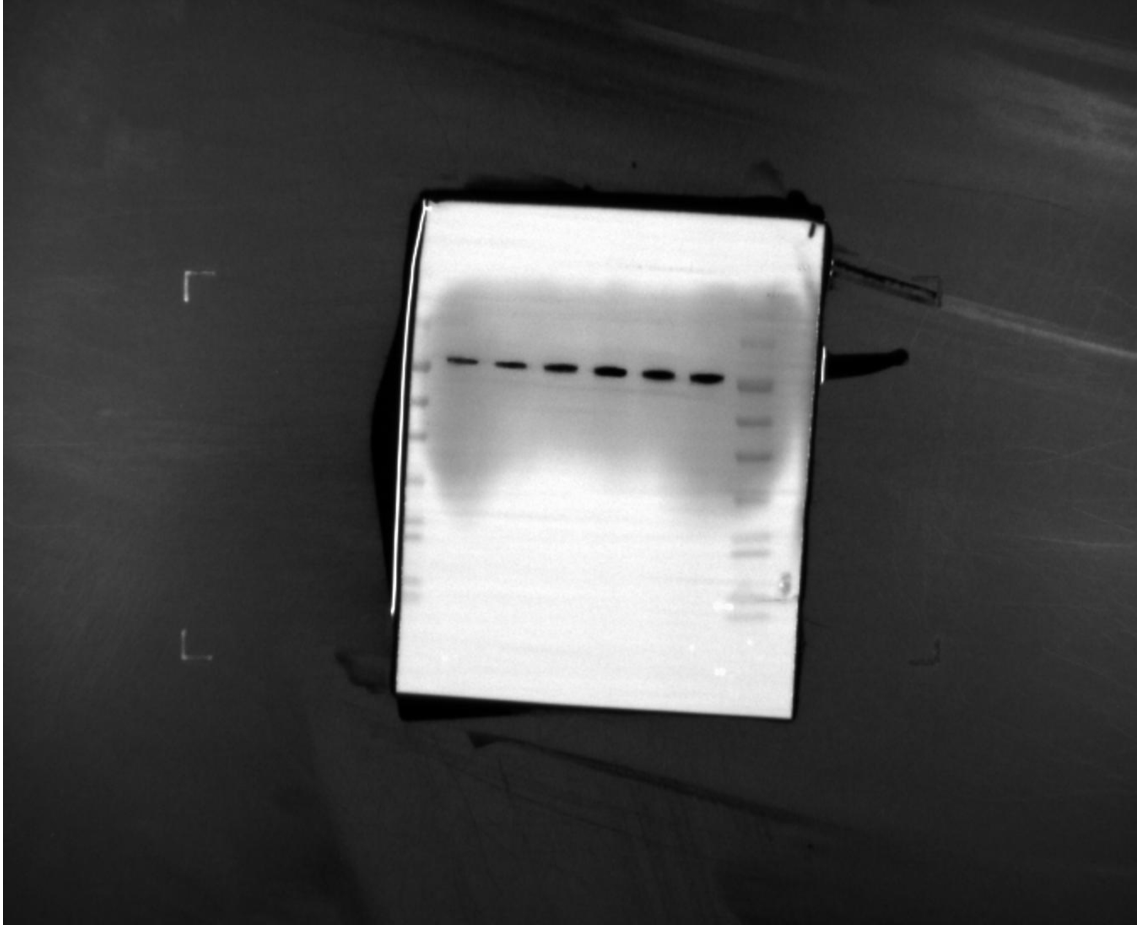
Figure 1B-YAP1

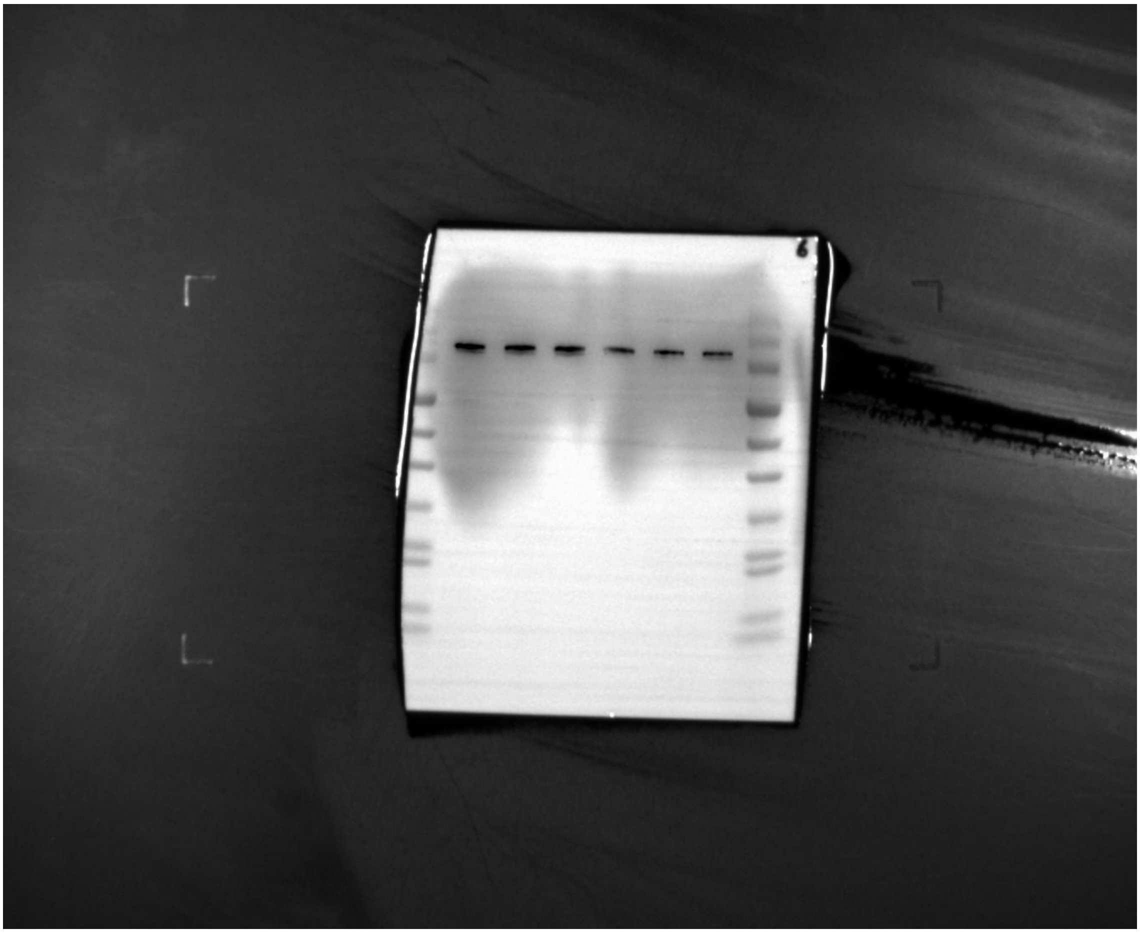
Figure 1B-pYAP1

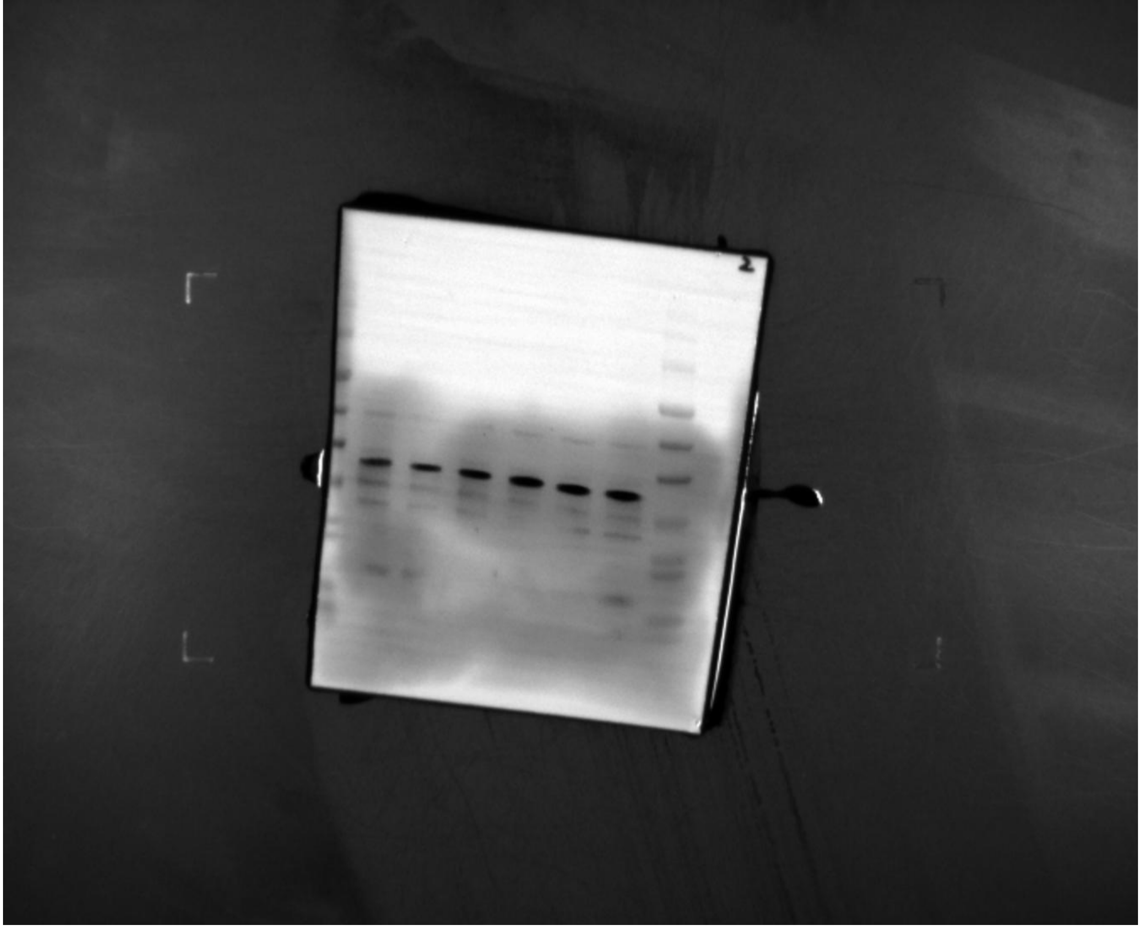

Figure 1B-CYR61

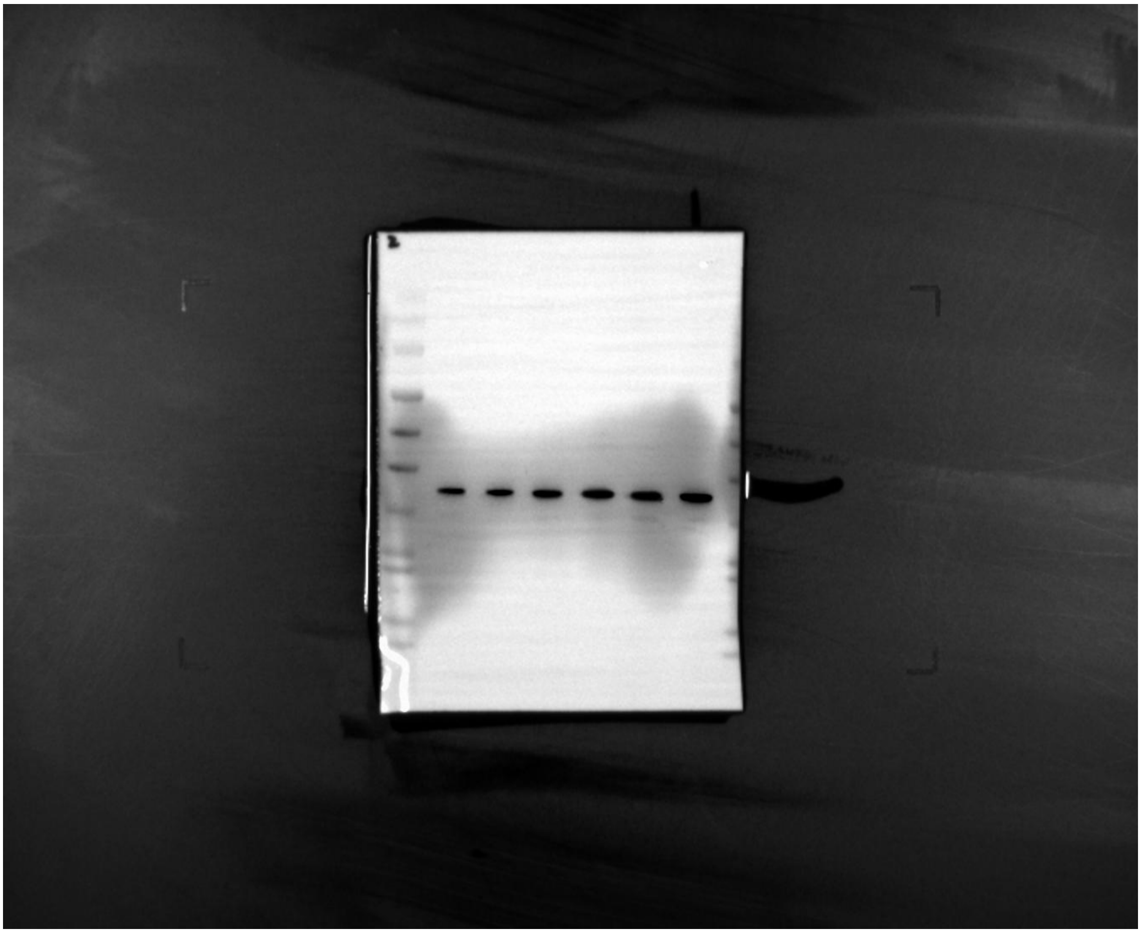

Figure 1B-AREG

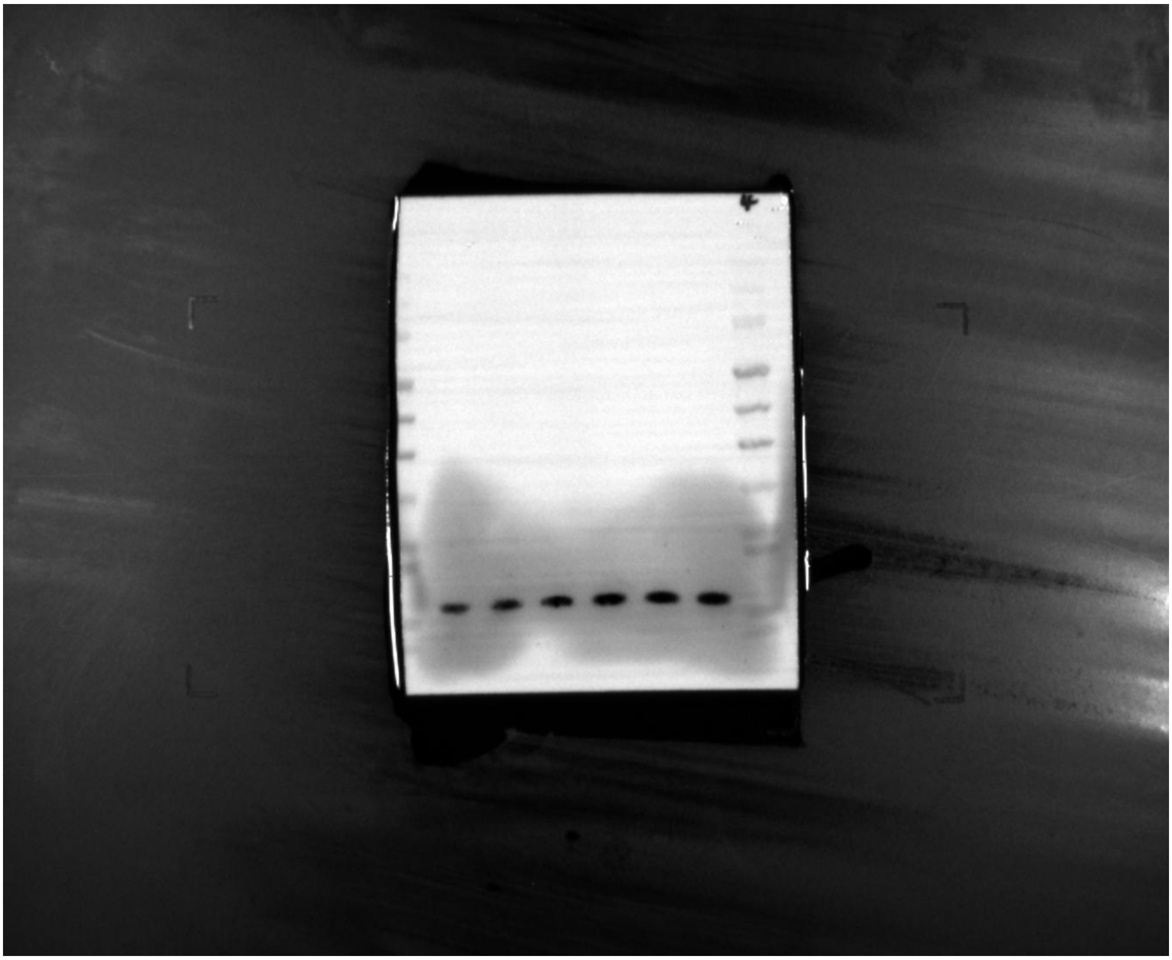

Figure 1B-BIRC5

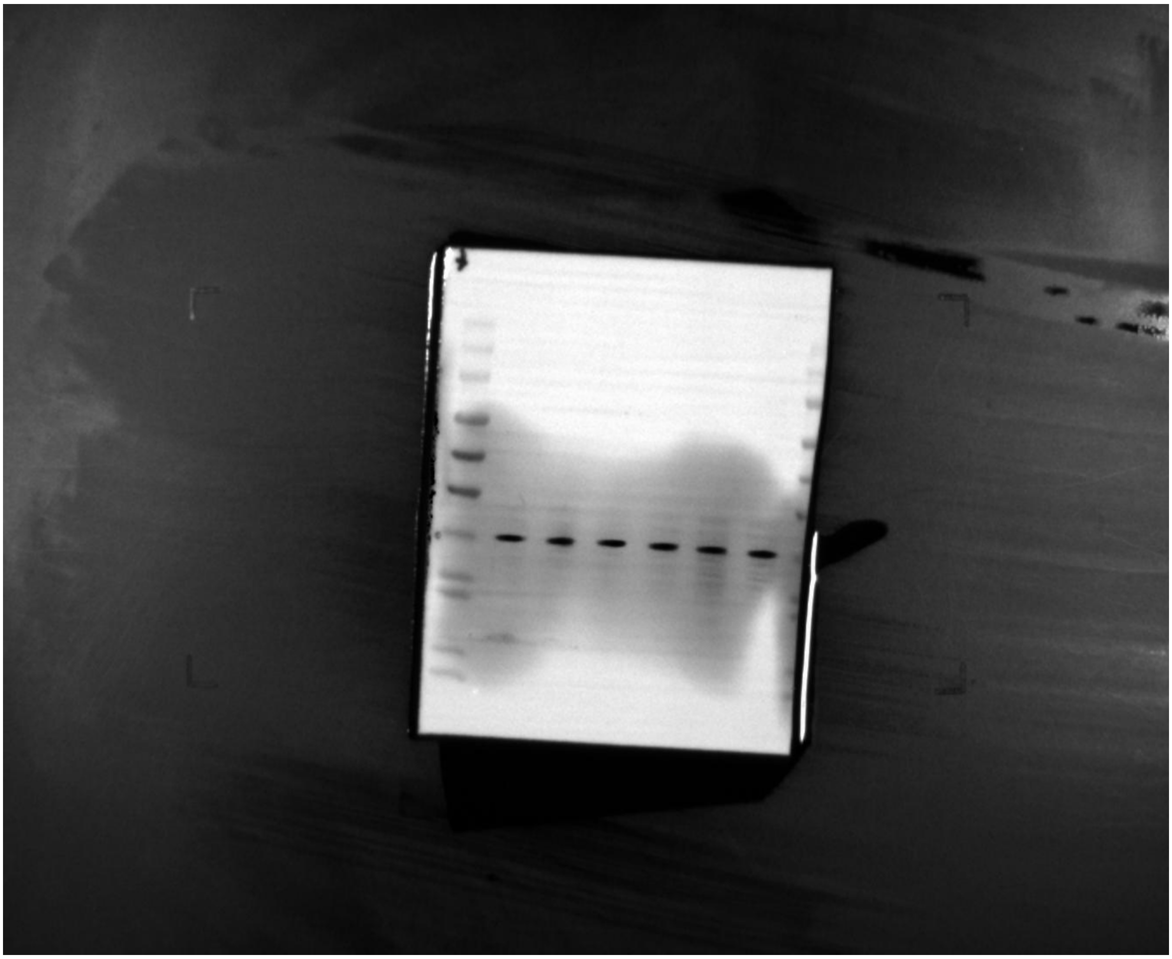

Figure 1B-GAPDH

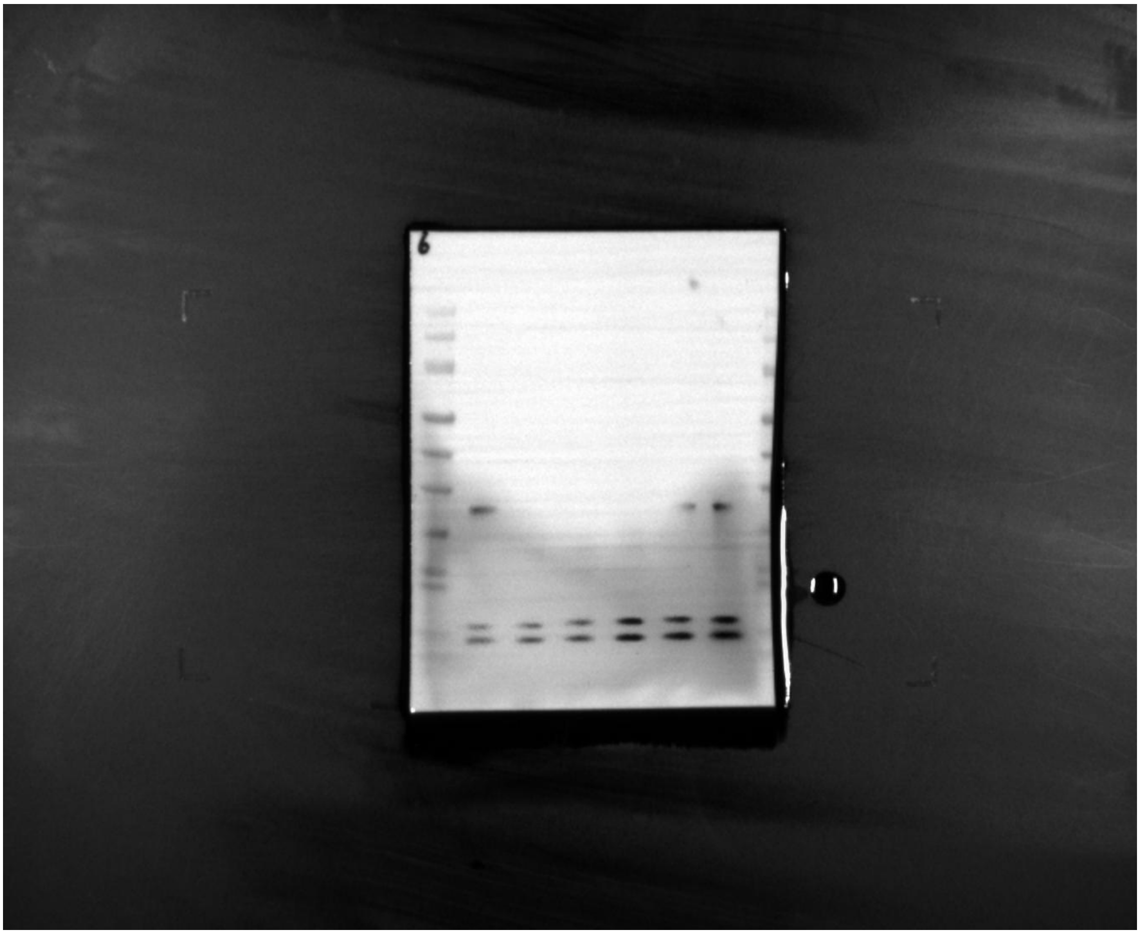
Figure 2C-LC3-I/II

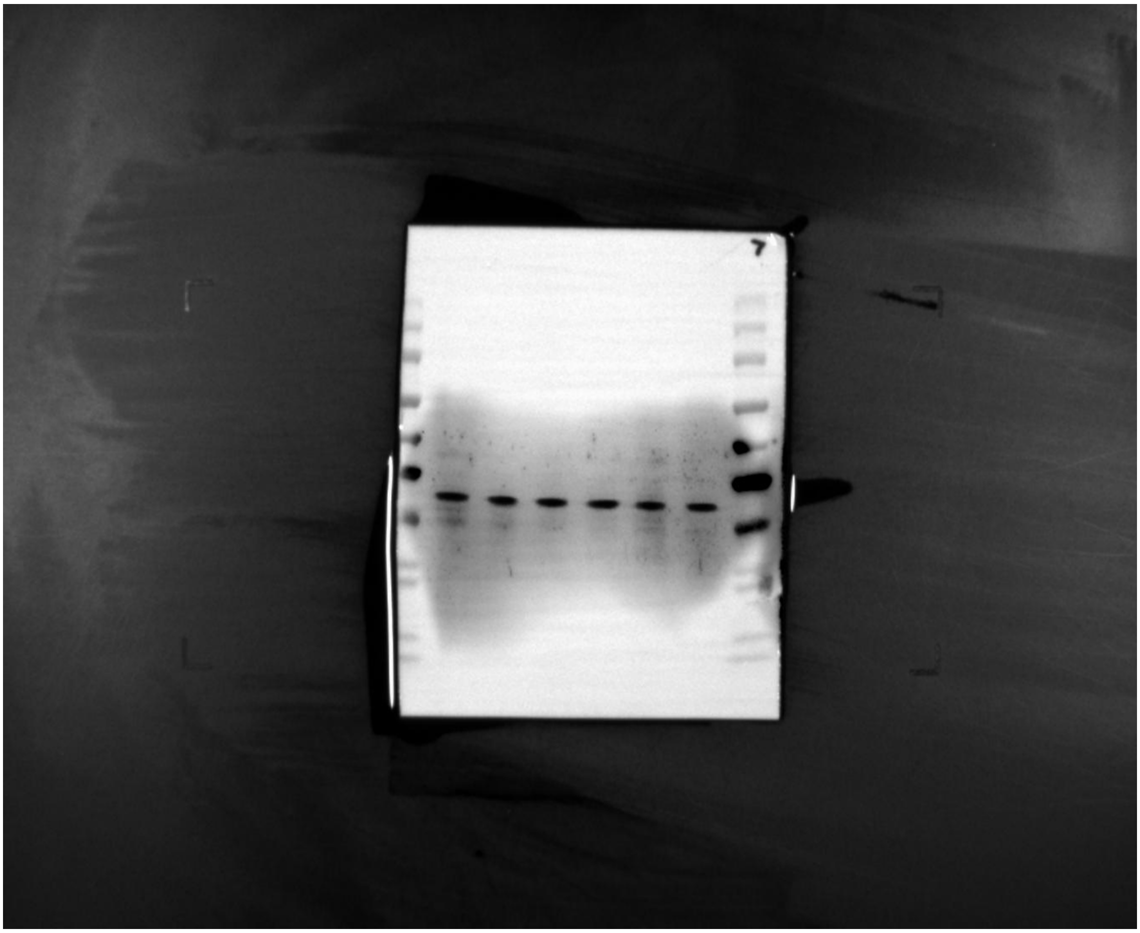
Figure 2C-GAPDH

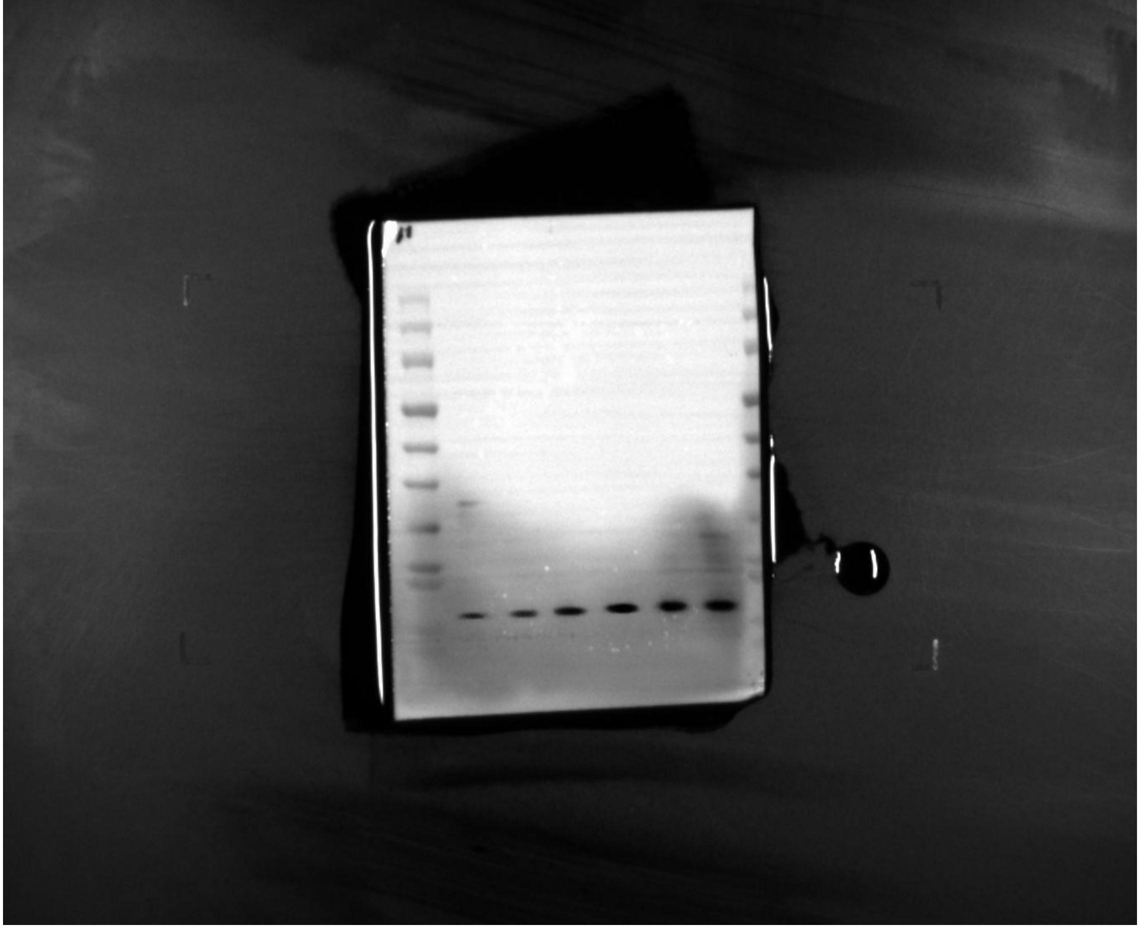

Figure 2E-FUND1

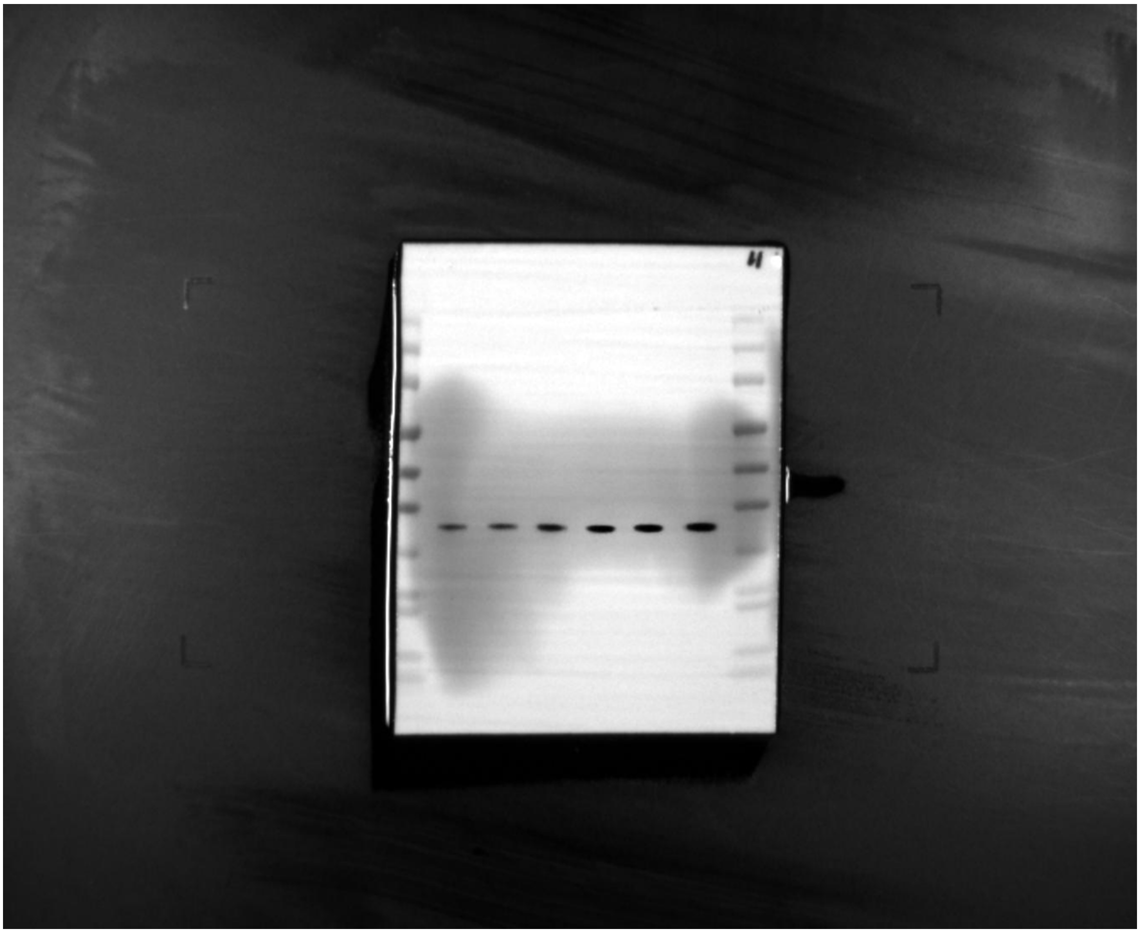

Figure 2E-PINK1

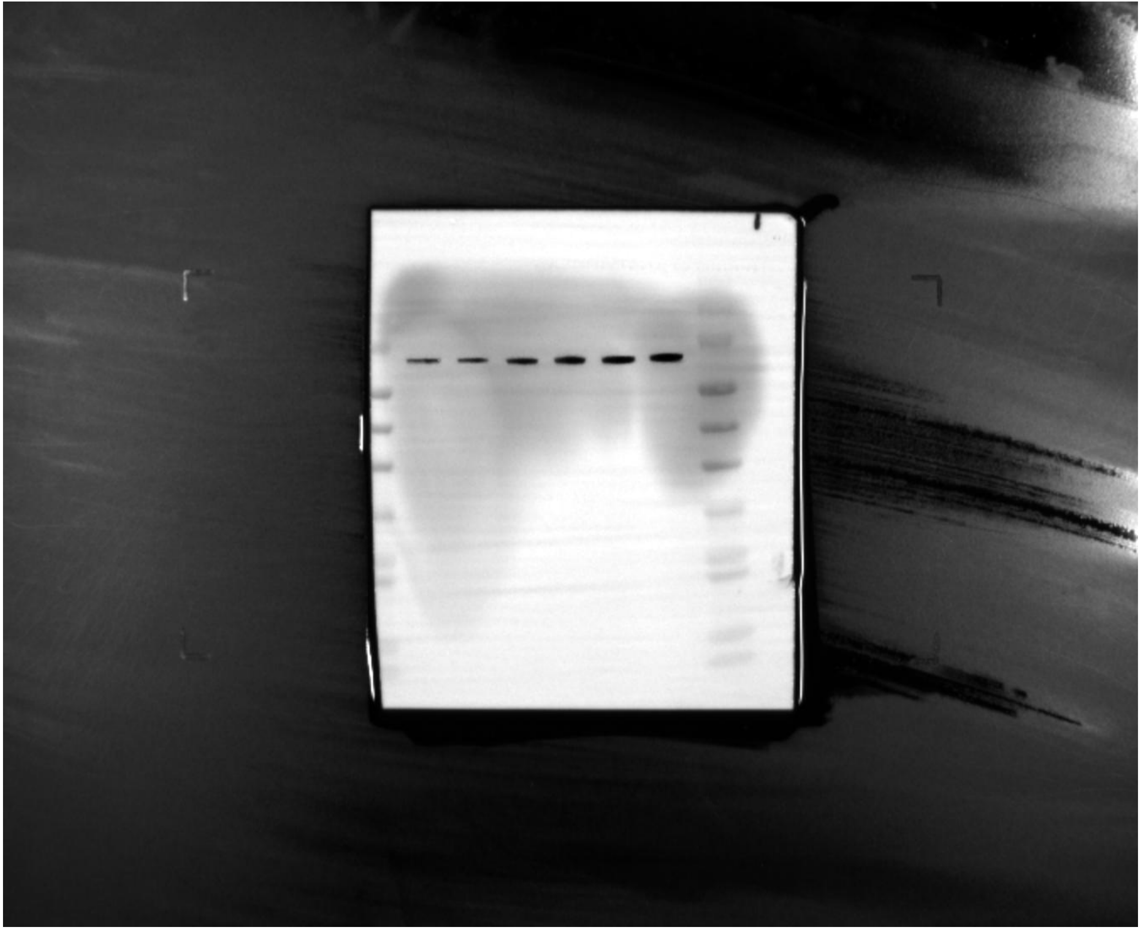

Figure 2E-TBK1

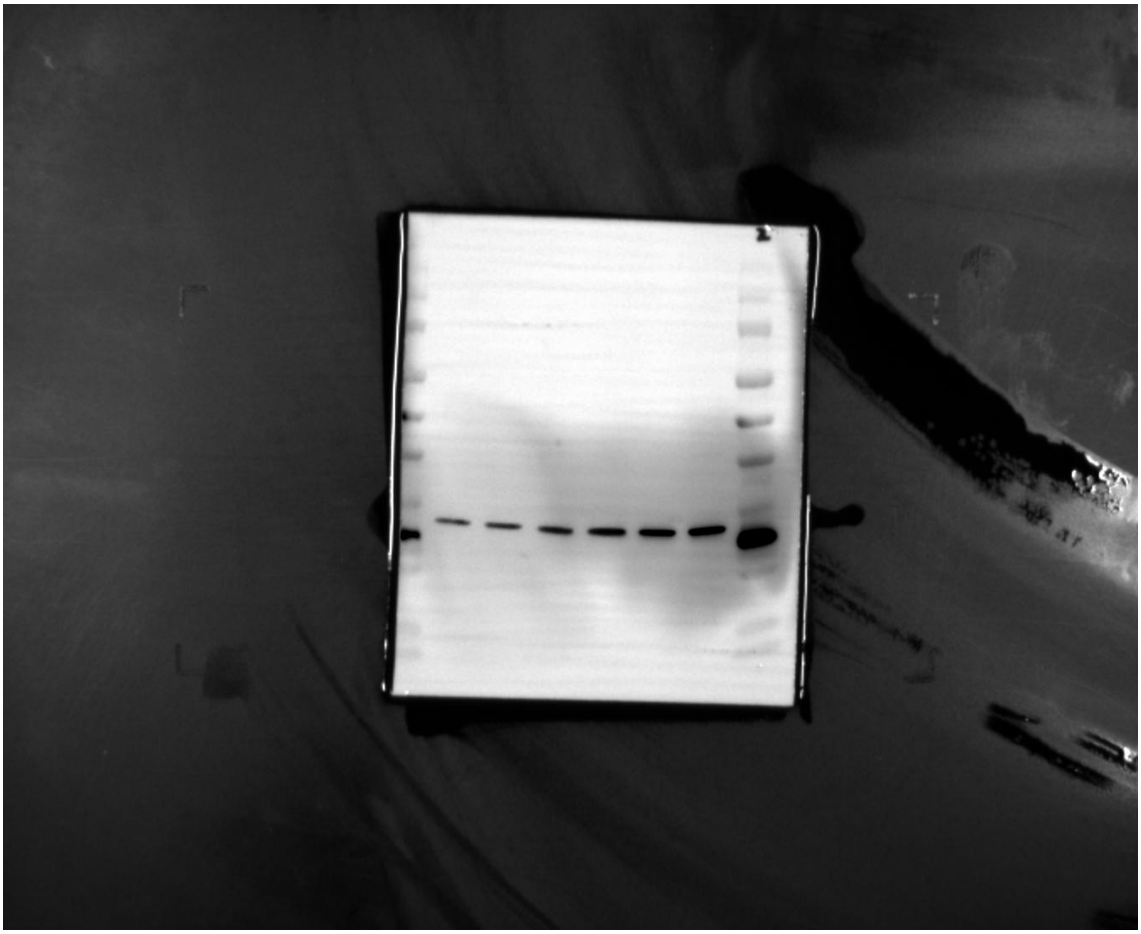

Figure 2E-HMGB1

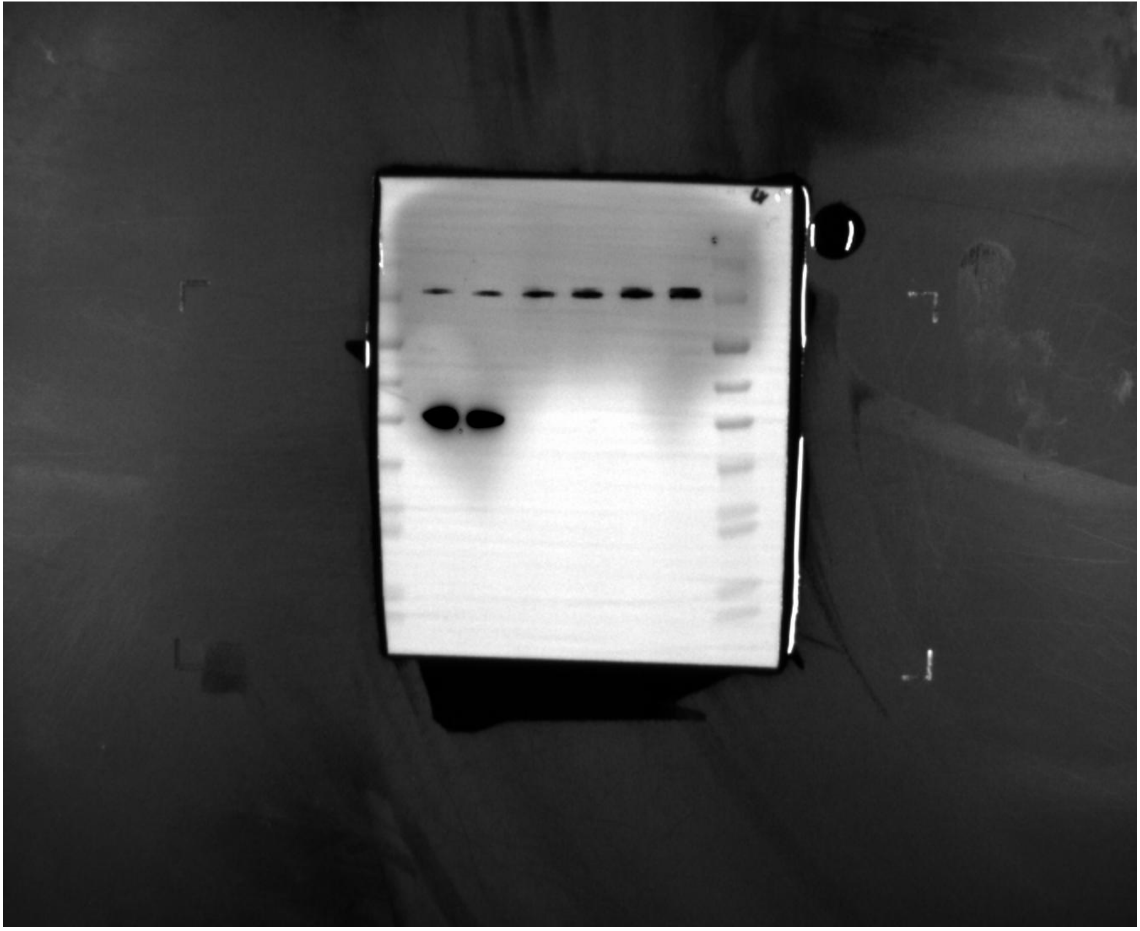

Figure 2E-DAPK

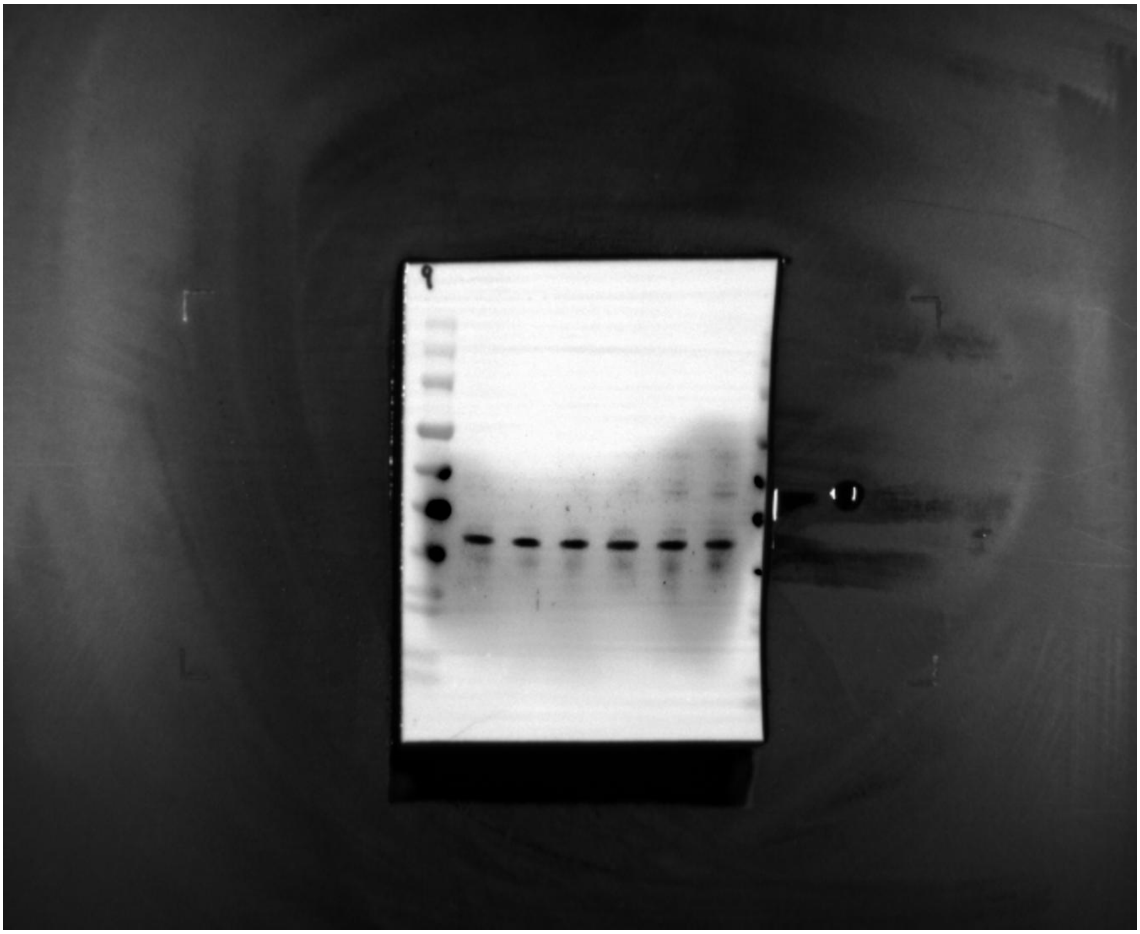

Figure 2E-GAPDH

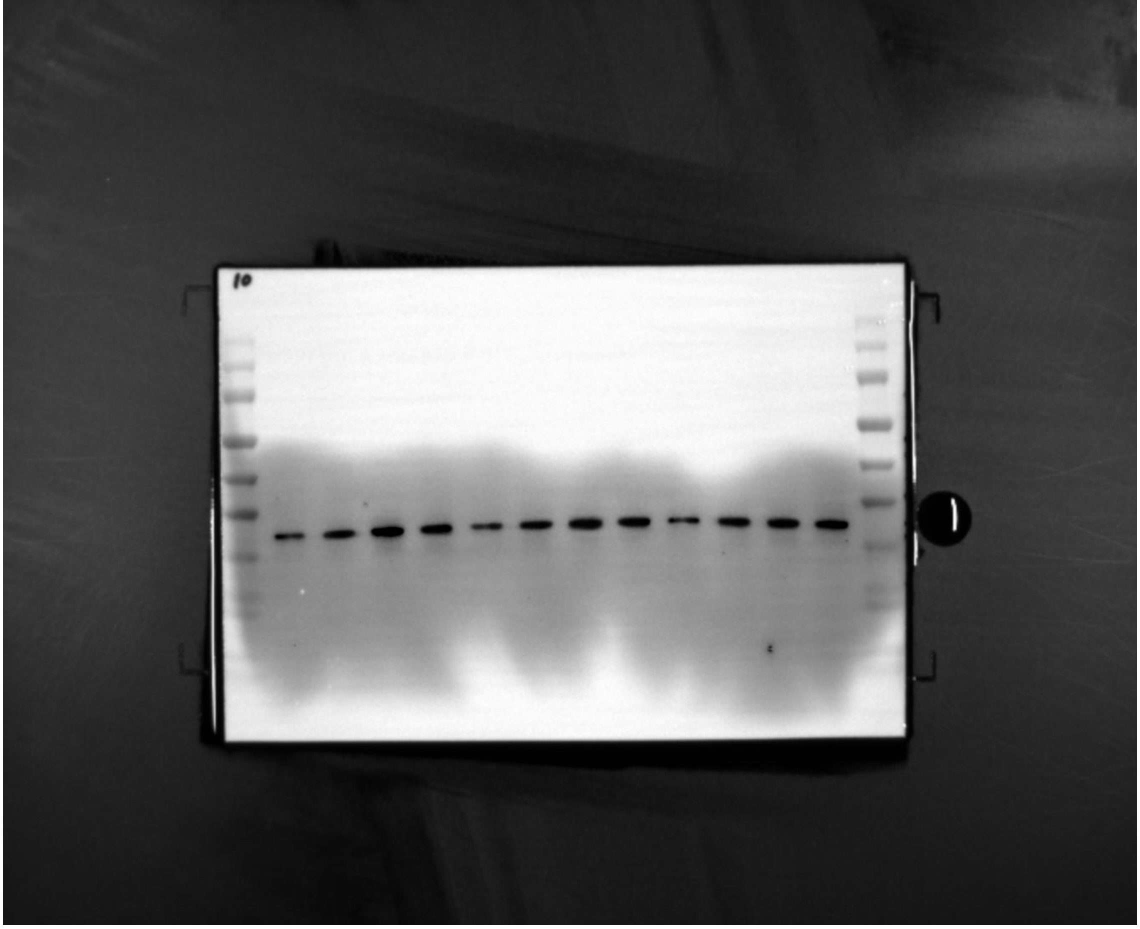

Figure 3C-CTGF

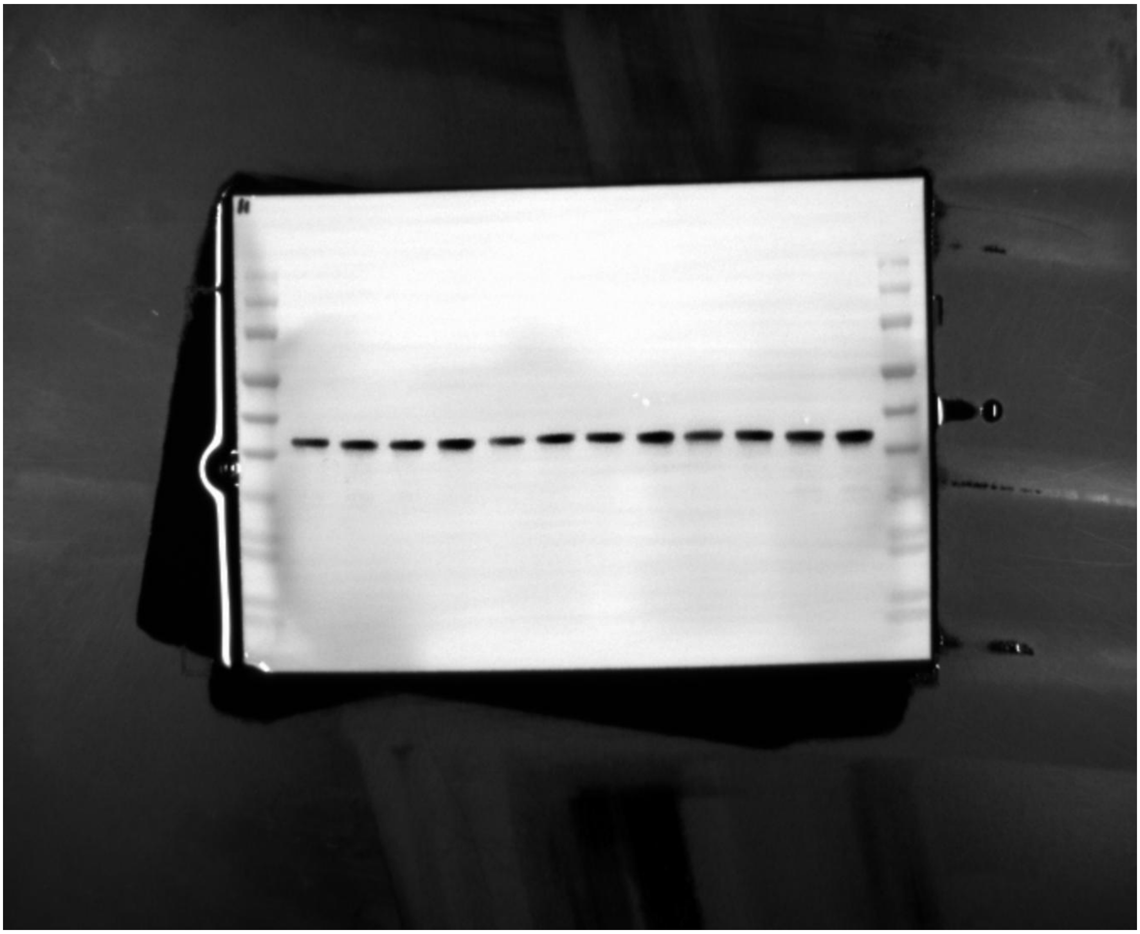

Figure 3C-CYR61

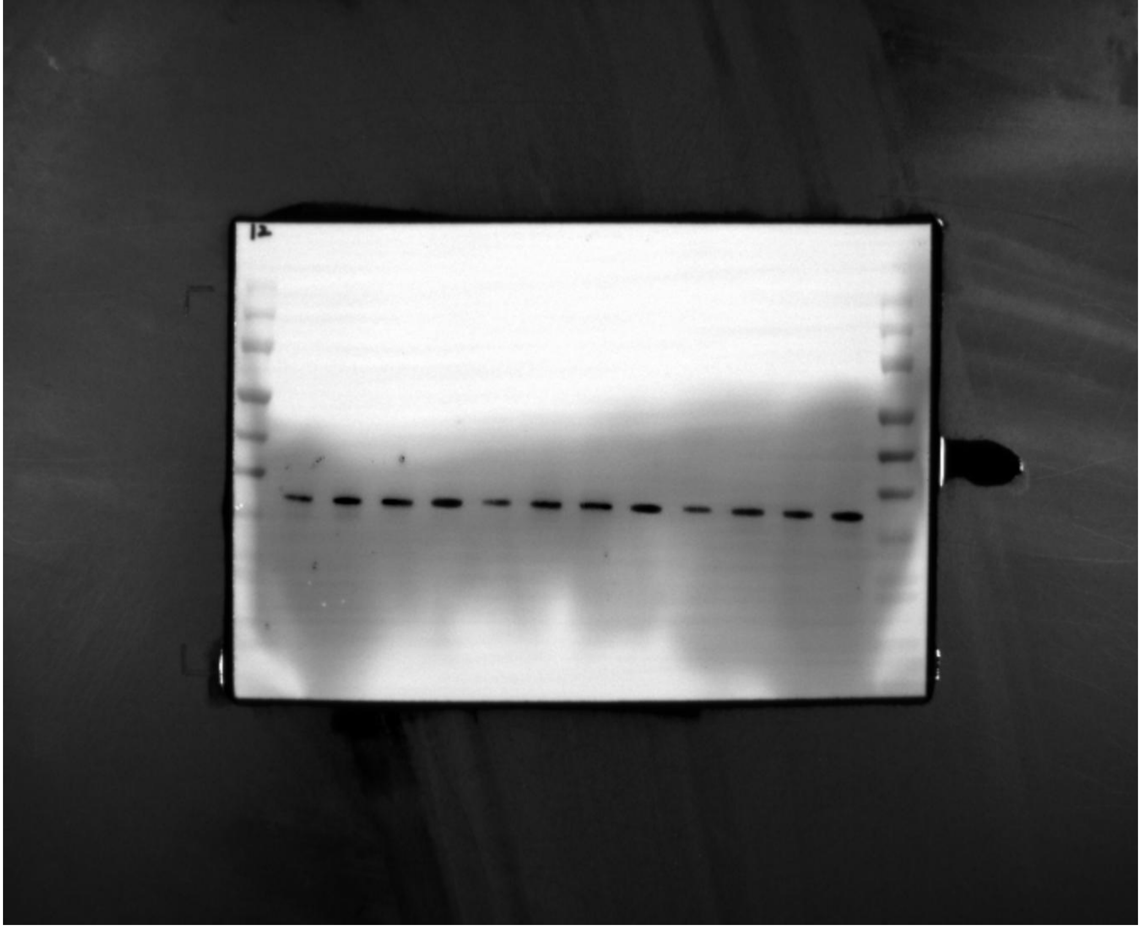

Figure 3C-AREG

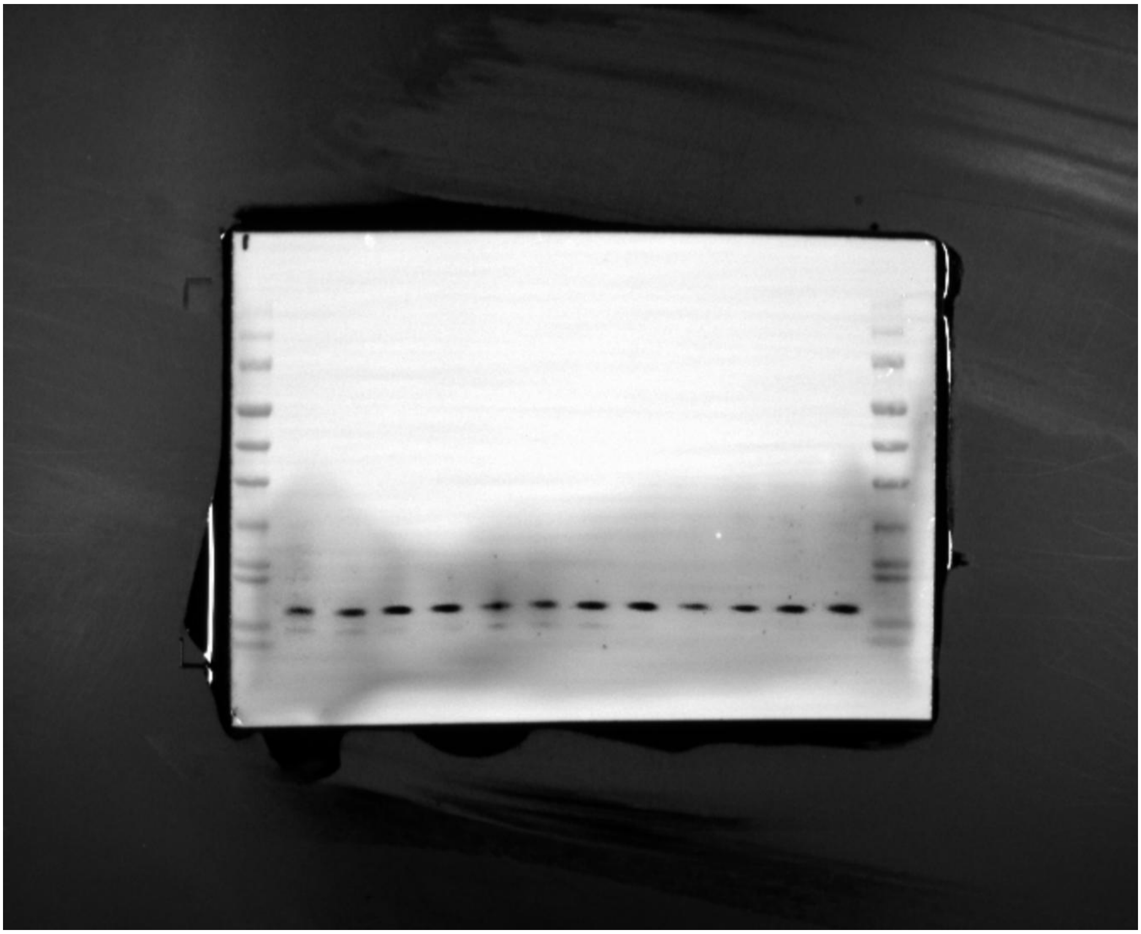

Figure 3C-BIRC5

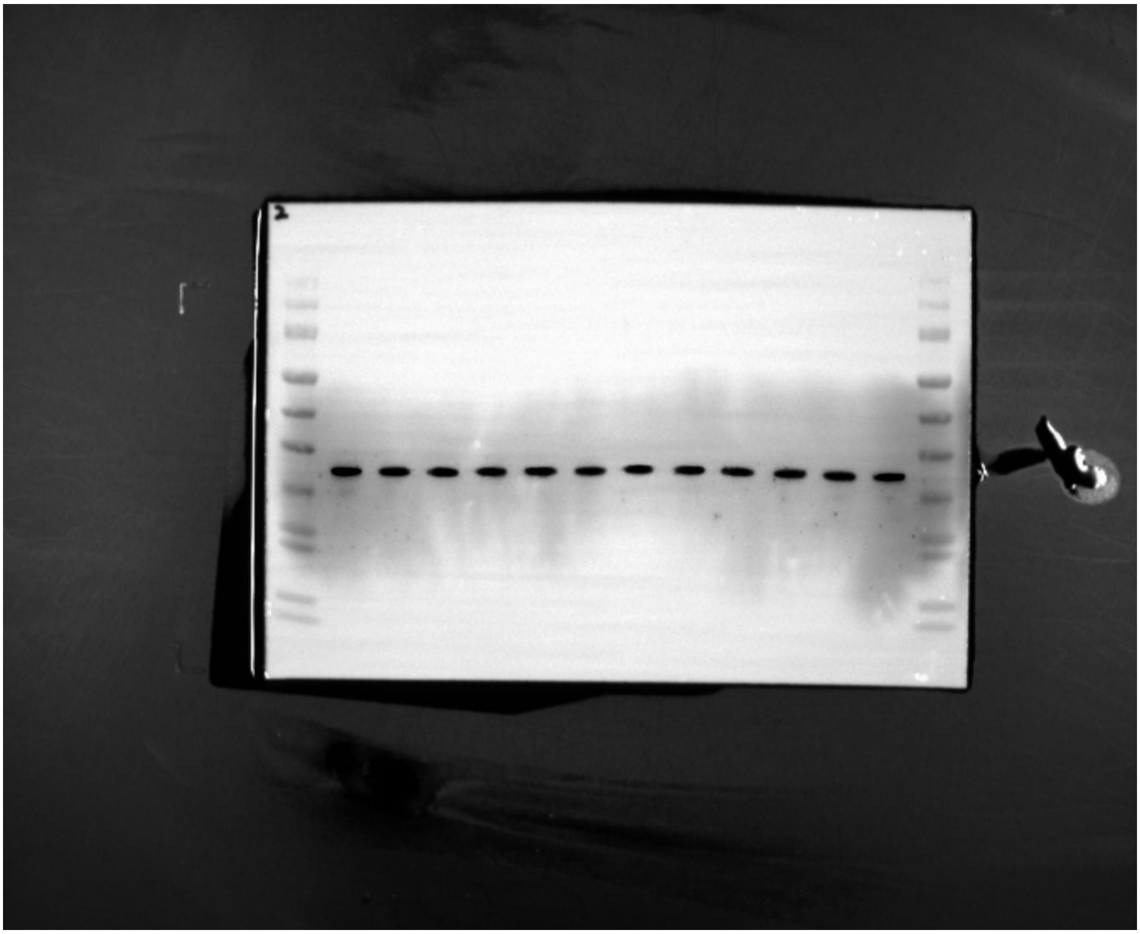

Figure 3C-GAPDH

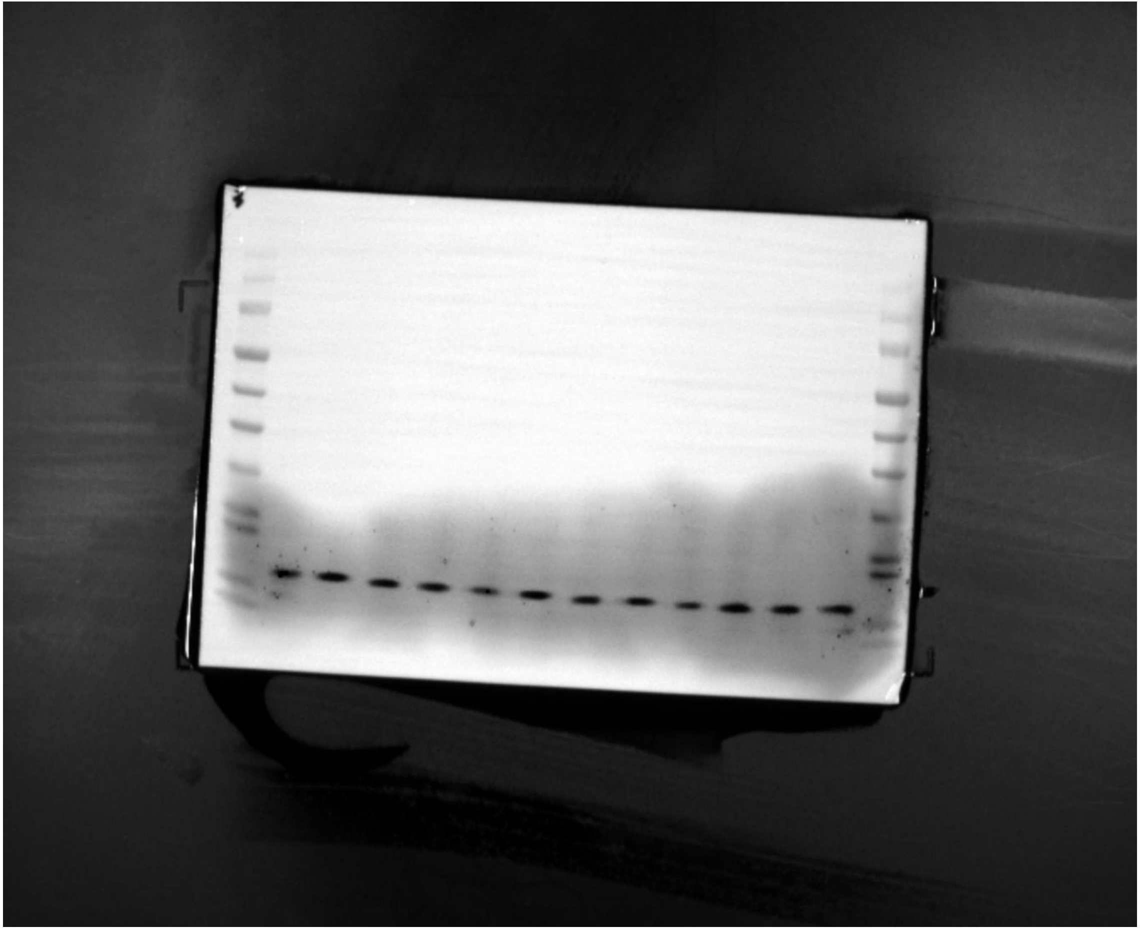

Figure 3E-FUND1

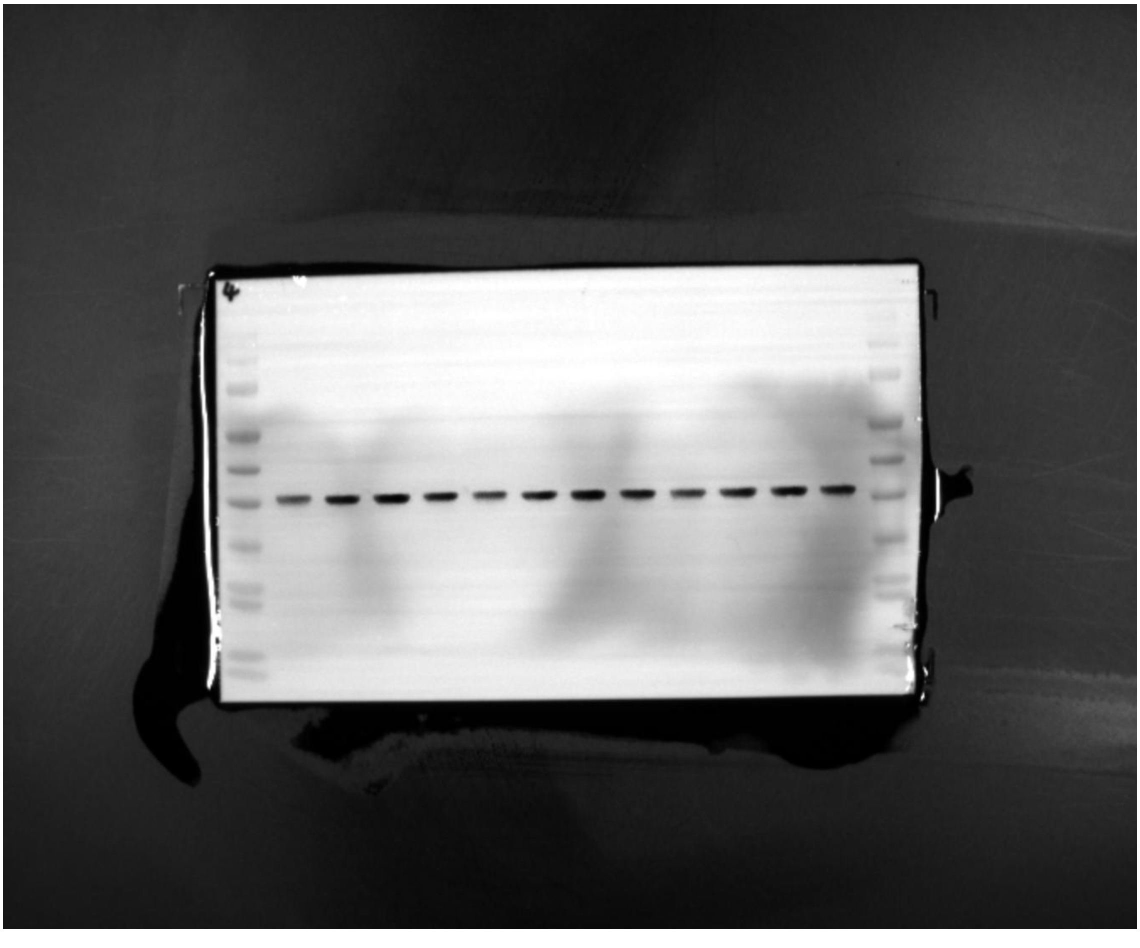

Figure 3E-PINK1

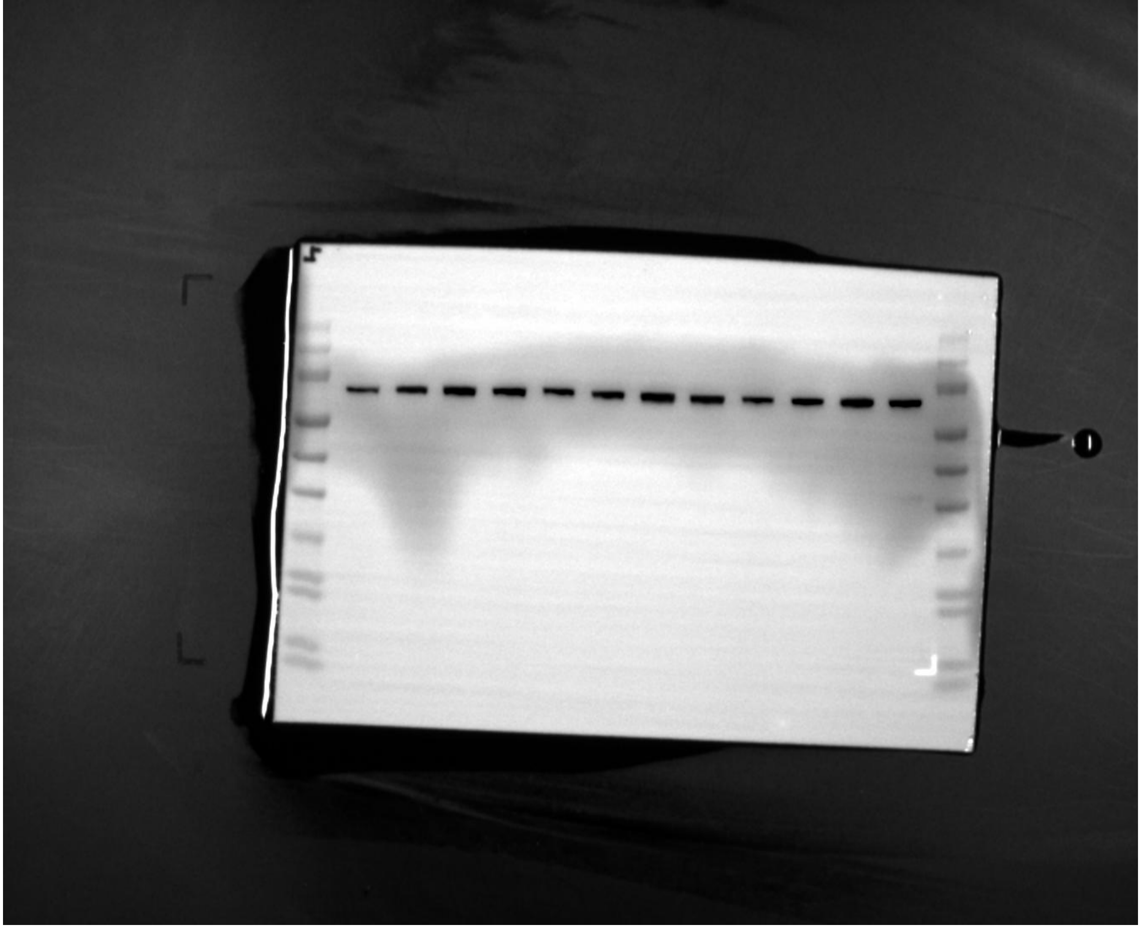

Figure 3E-TBK1

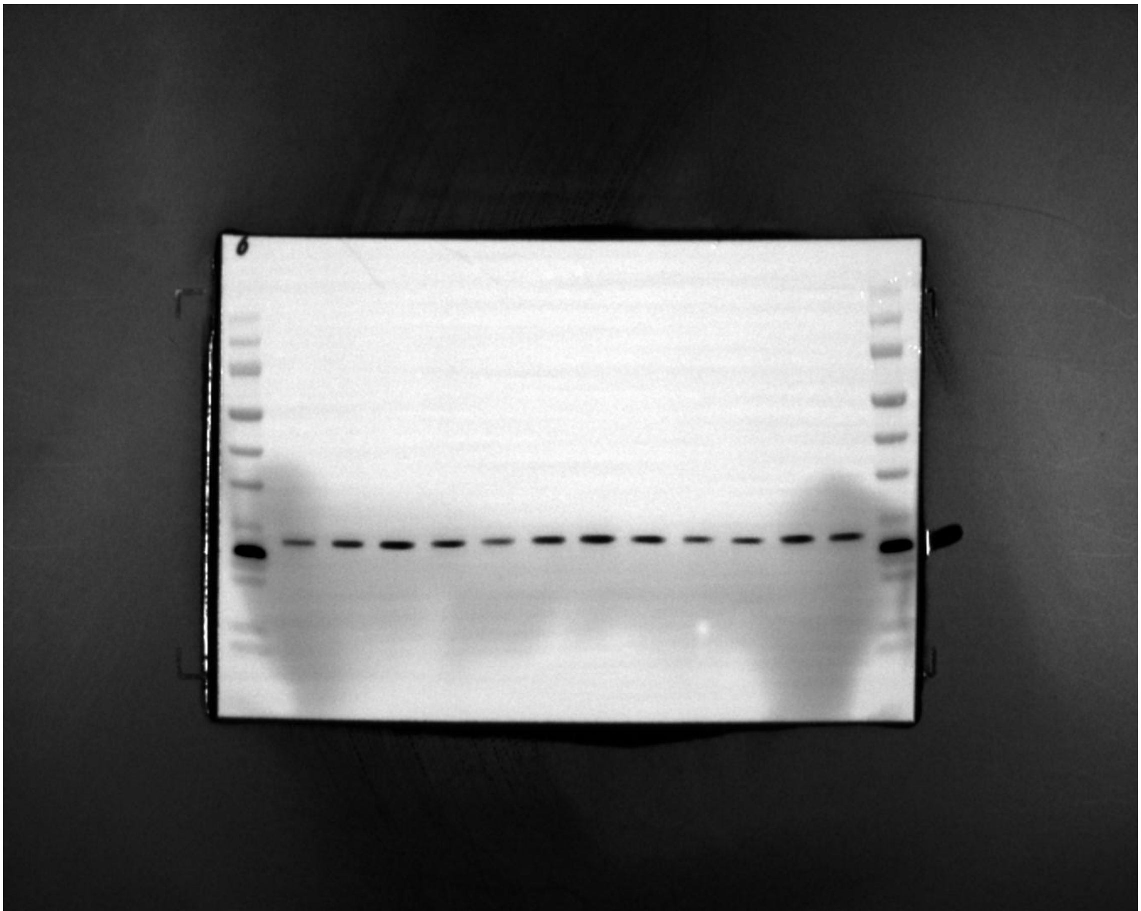

Figure 3E-HMGB1

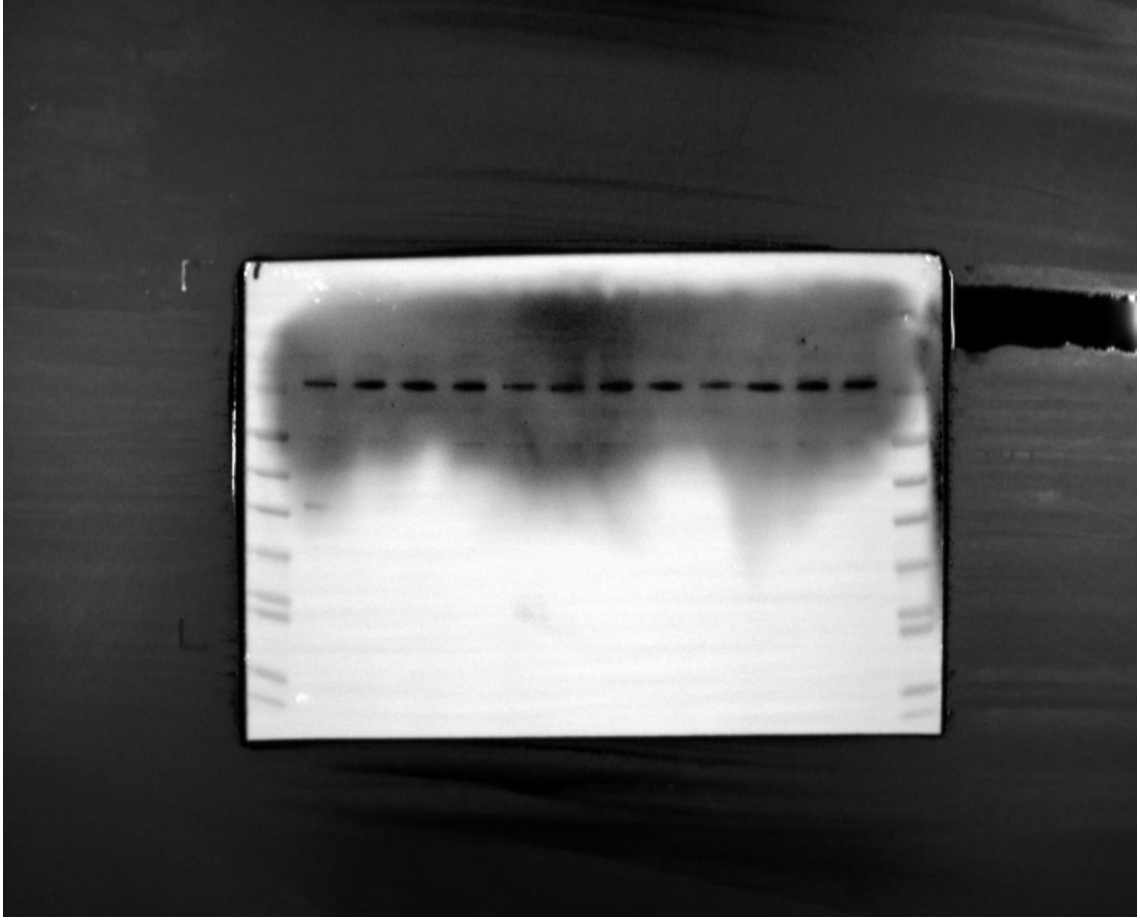

Figure 3E-DAPK

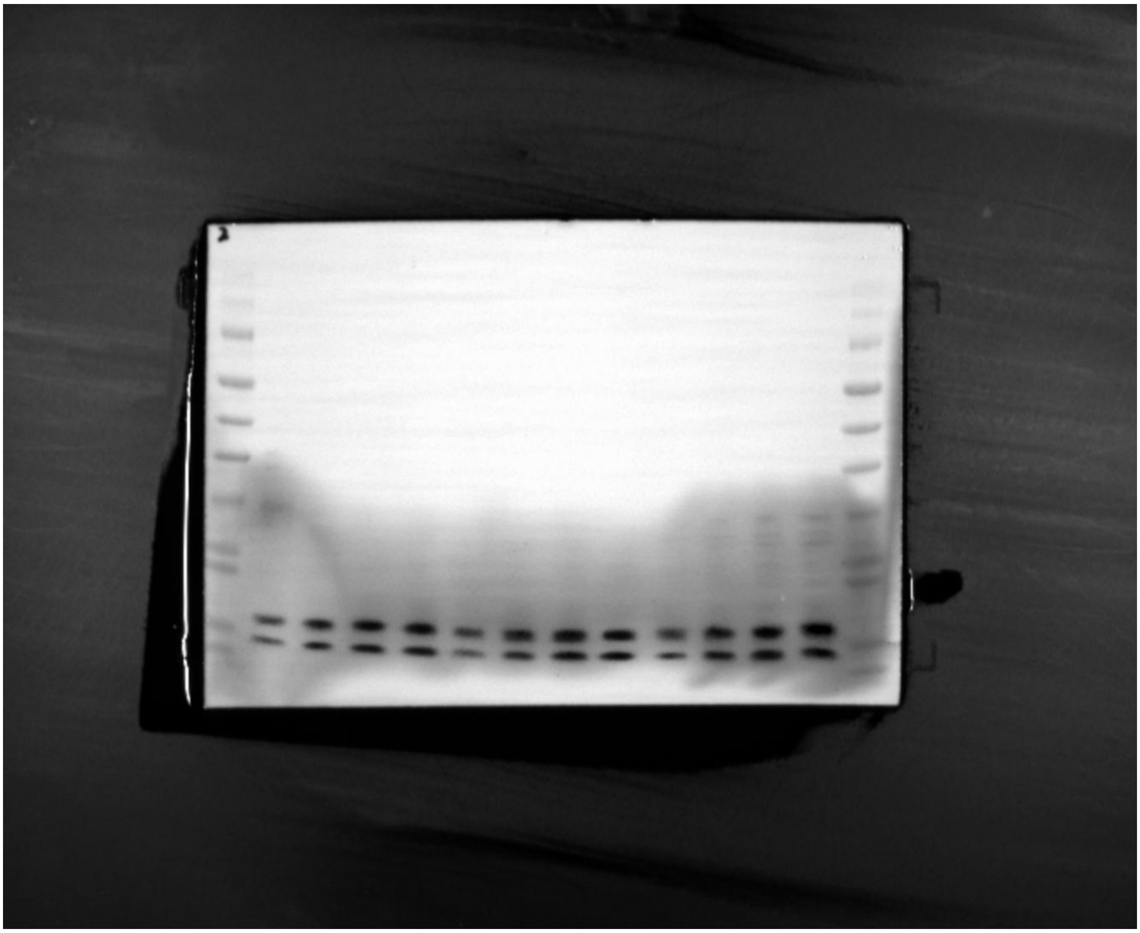

Figure 3E-LC3-I/II

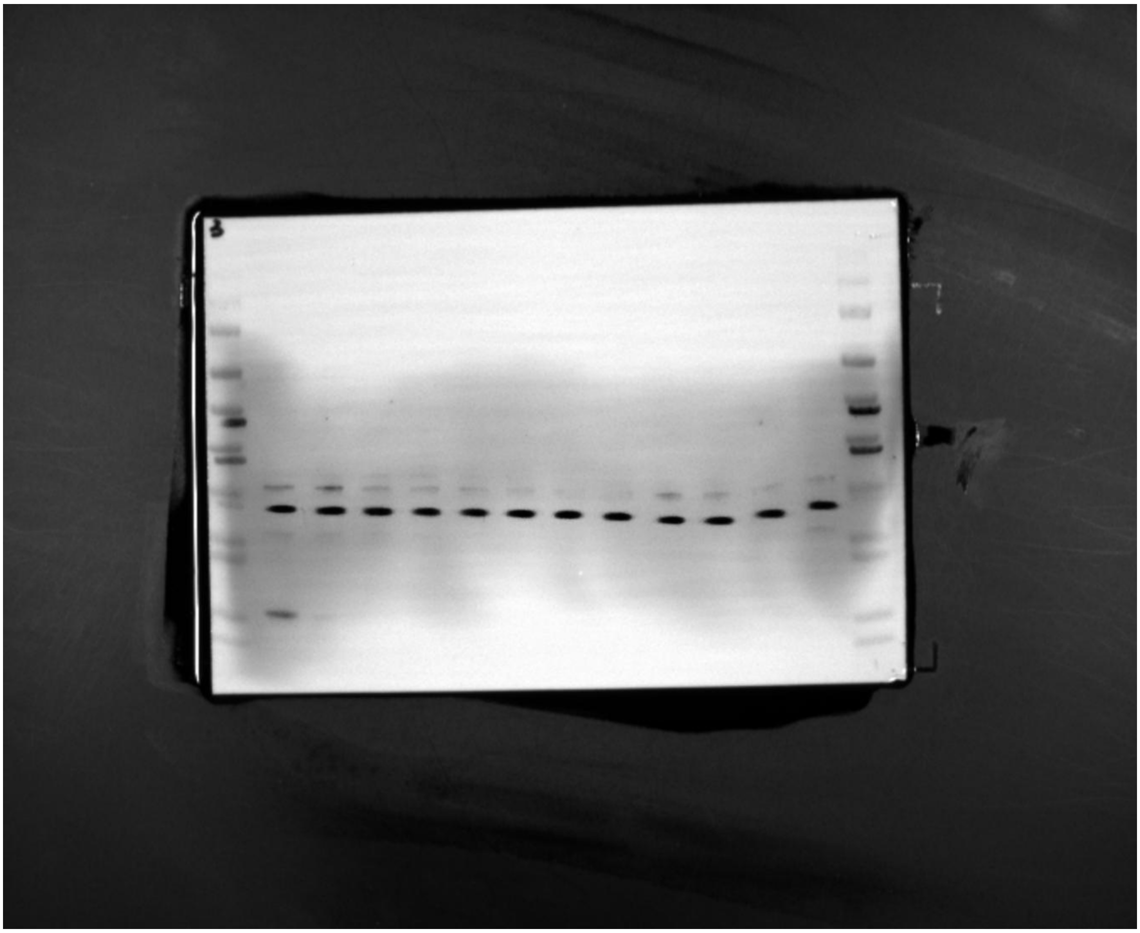

Figure 3E-GAPDH

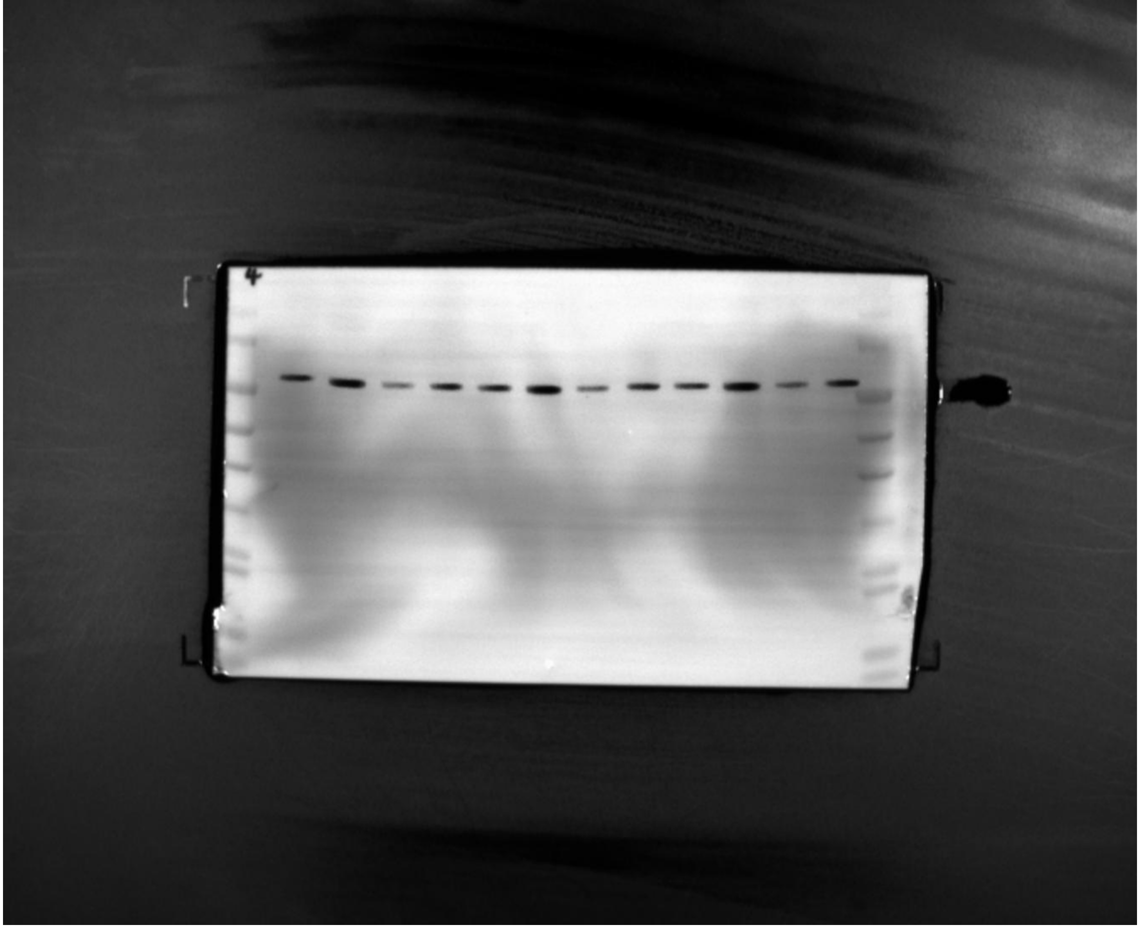

Figure 4B-YAP1

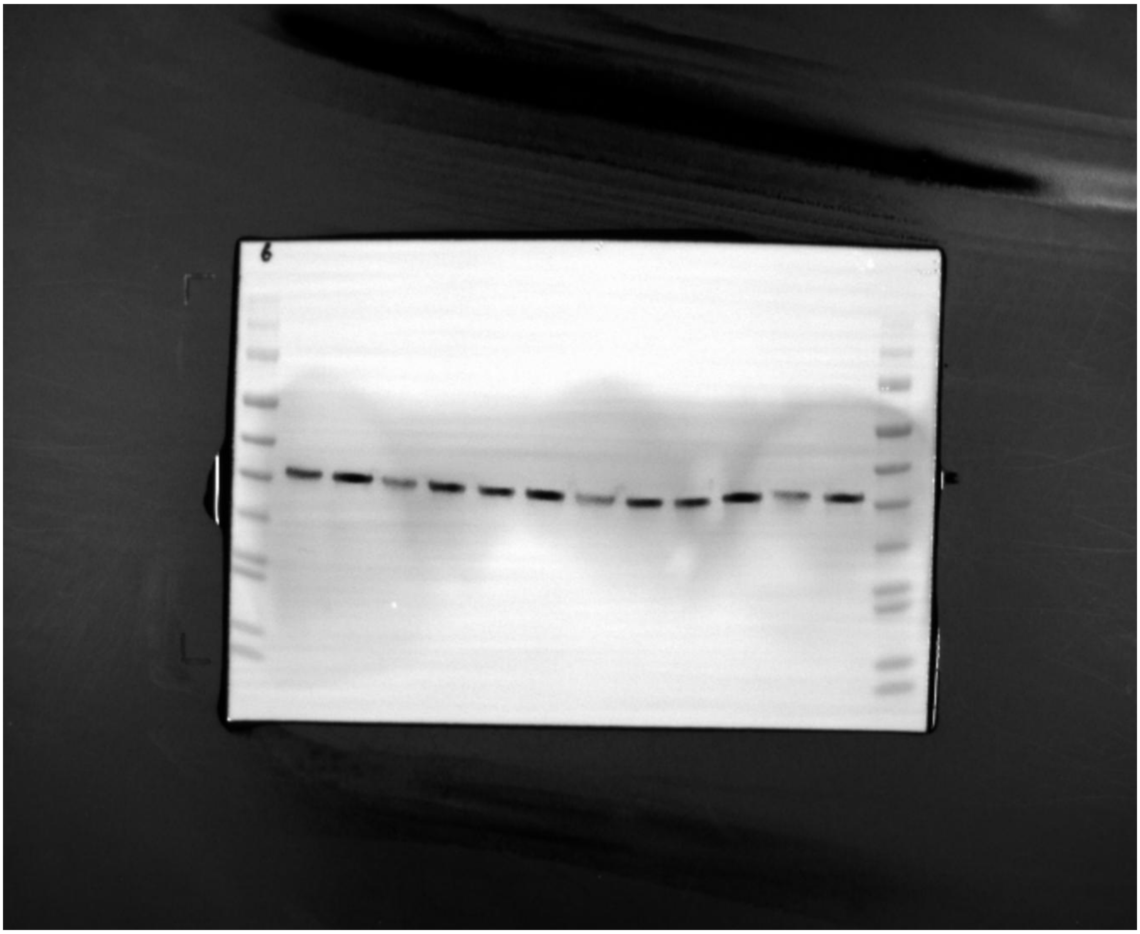

Figure 4B-CYR61

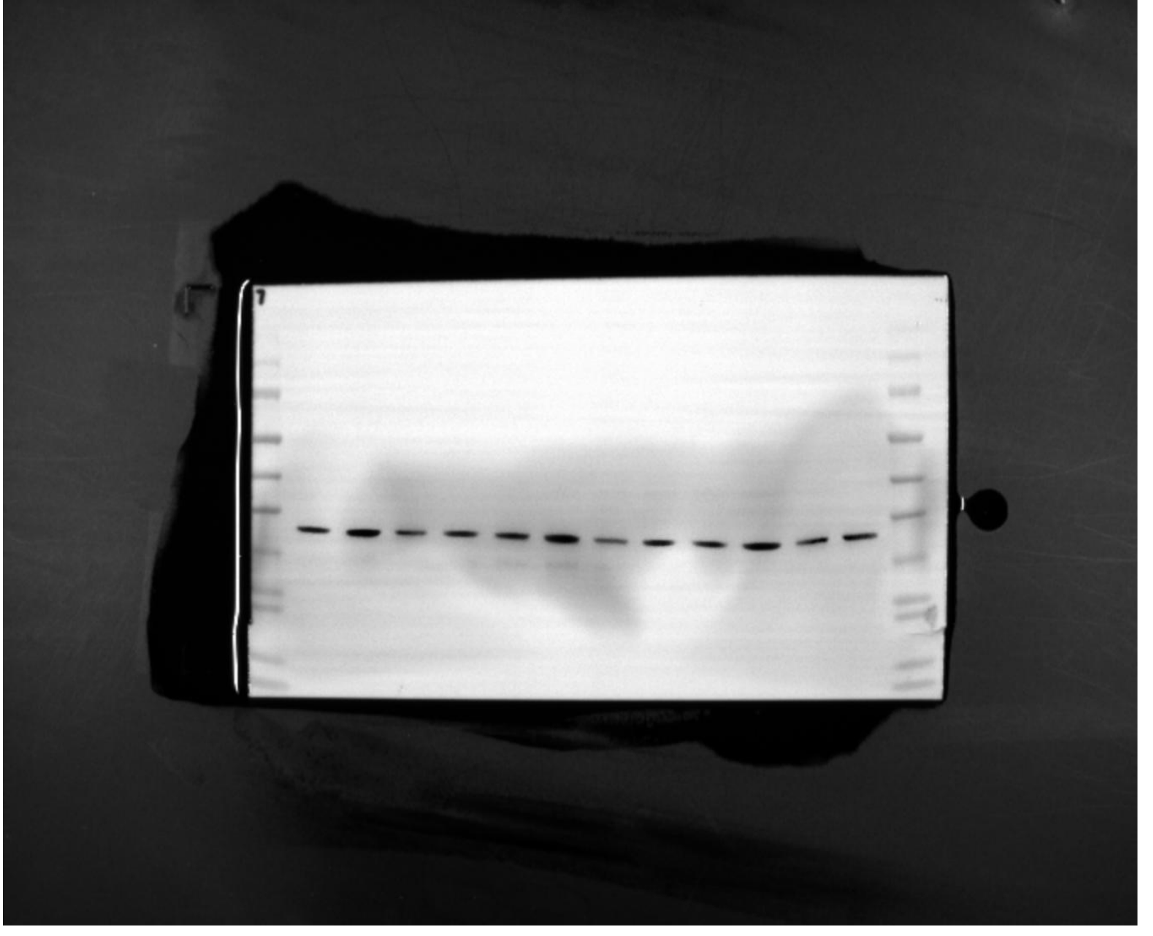

Figure 4B-AREG

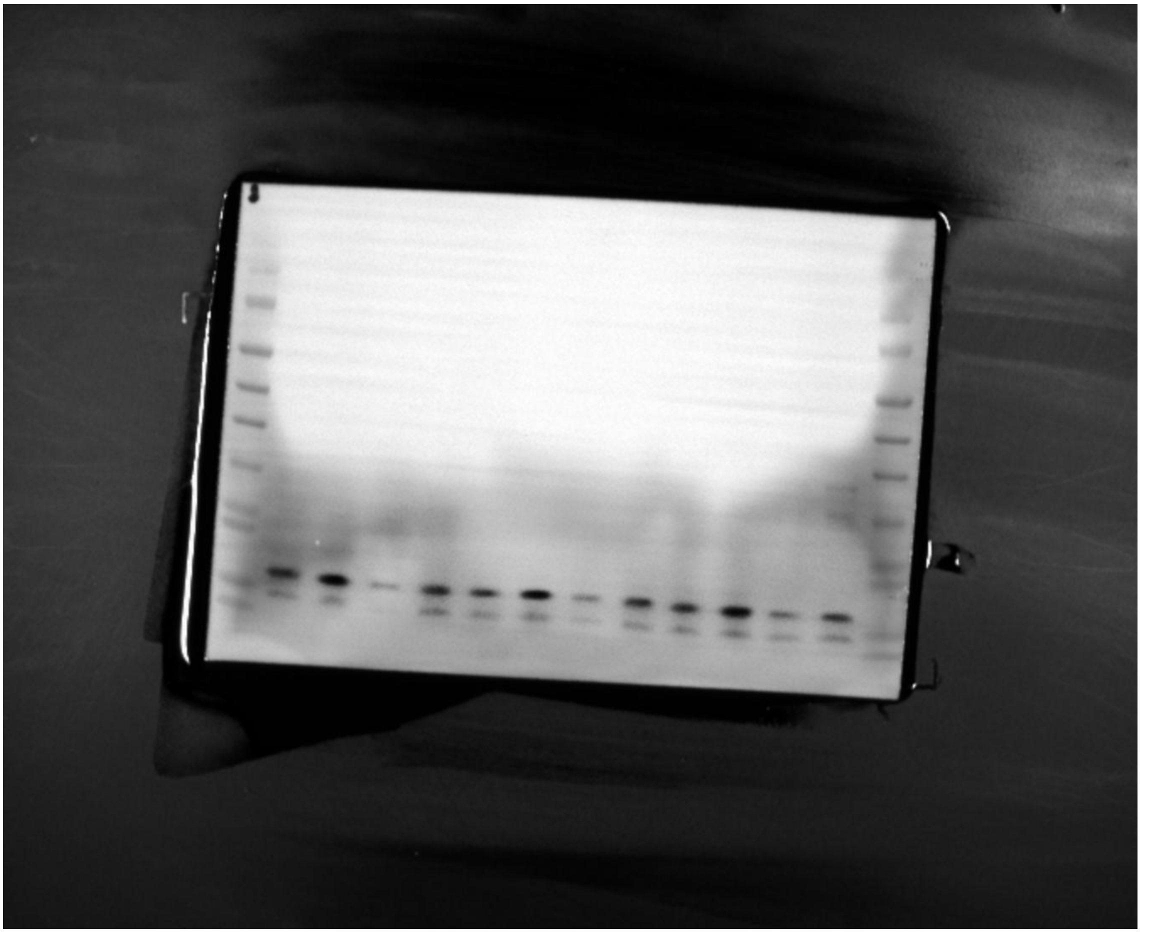

Figure 4B-BIRC5

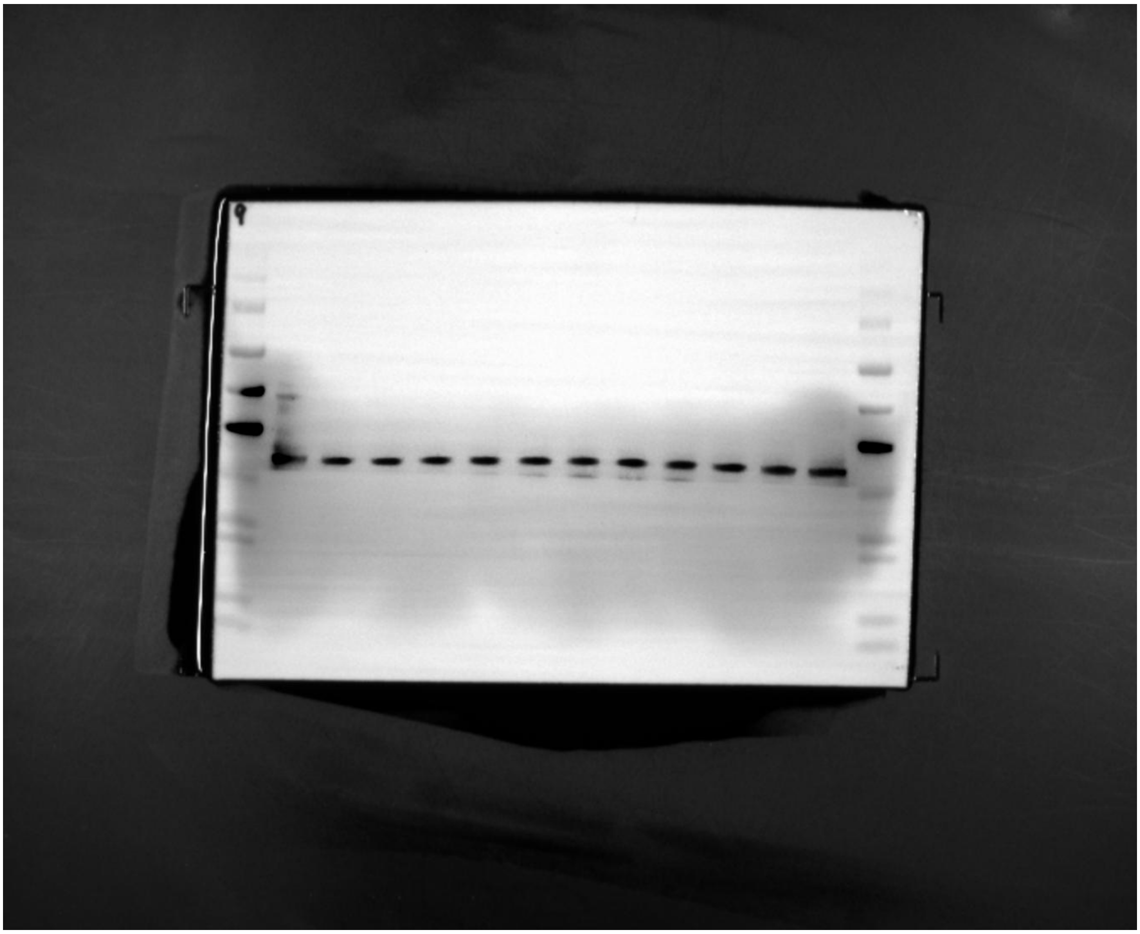

Figure 4B-GAPDH

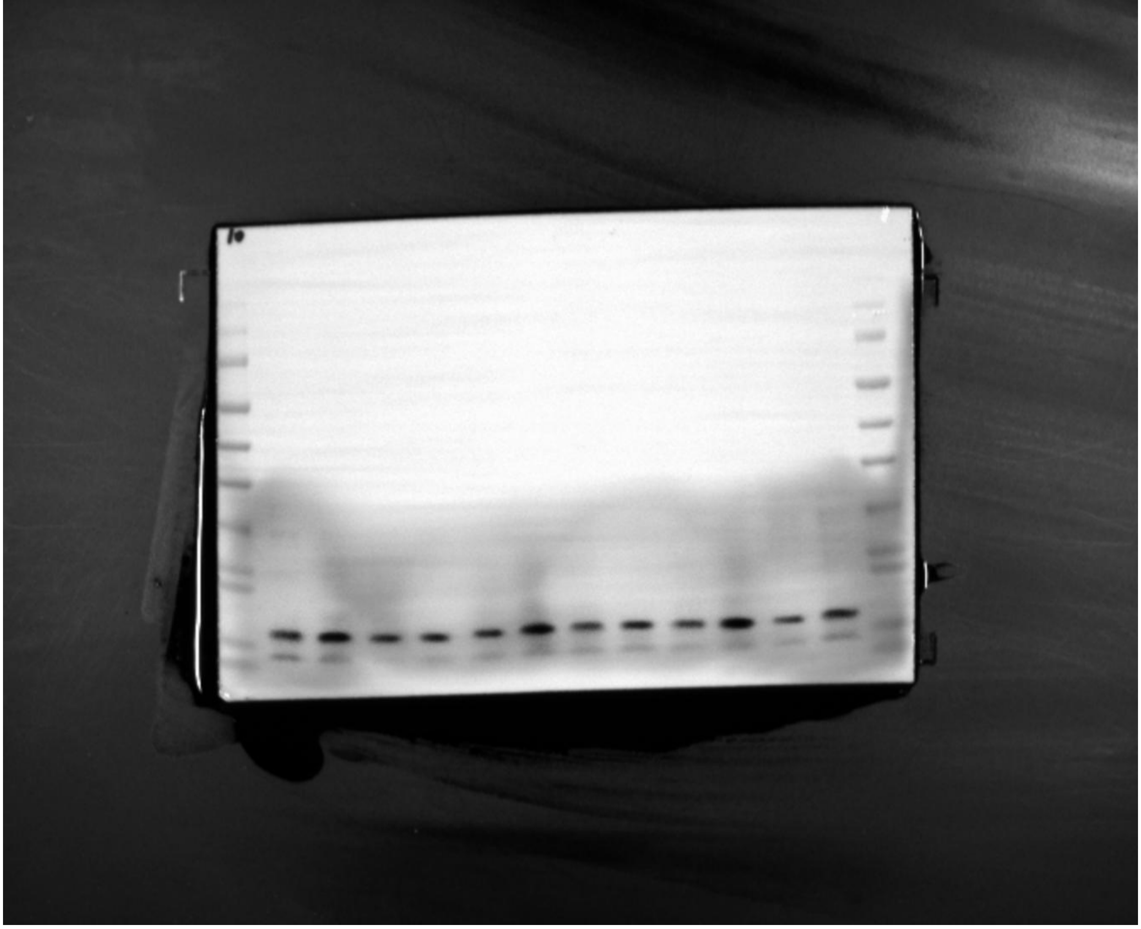

Figure 4E-FUND1

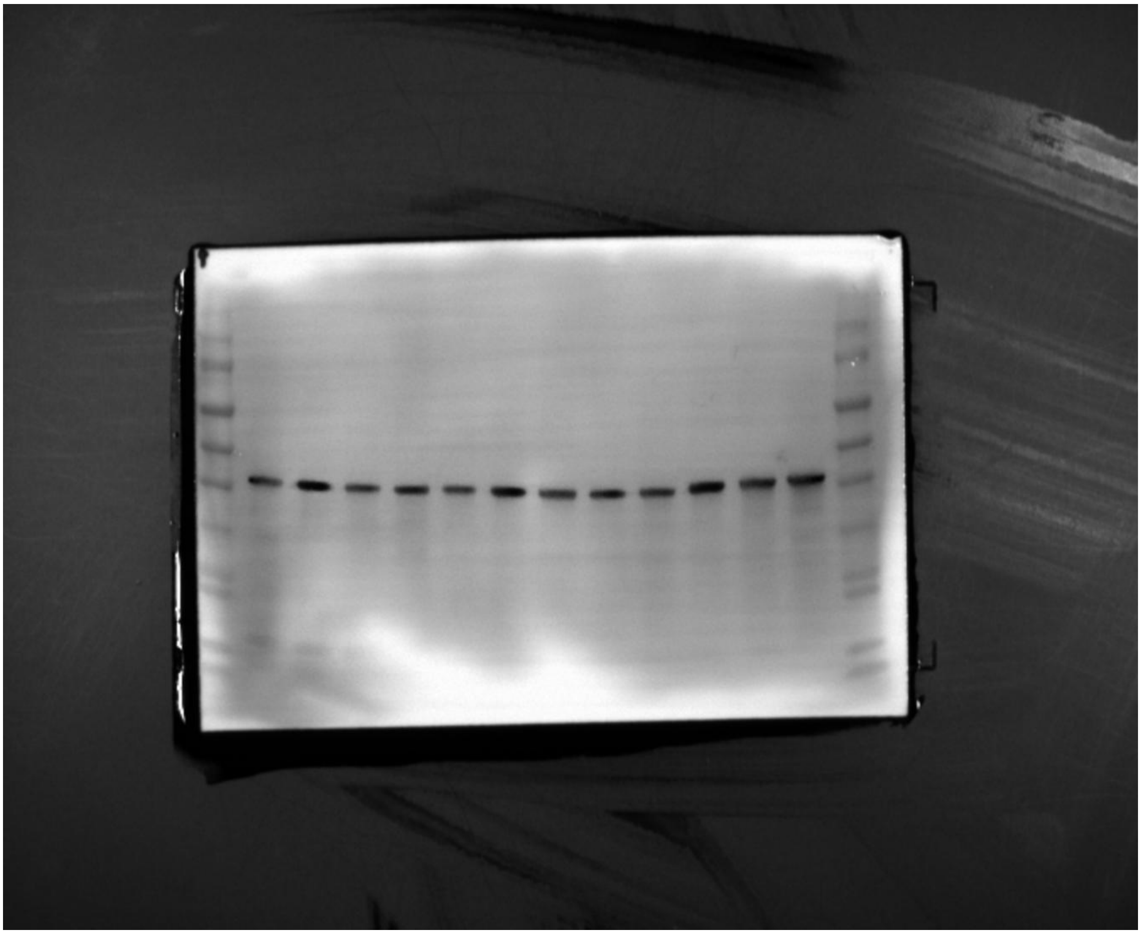

Figure 4E-PINK1

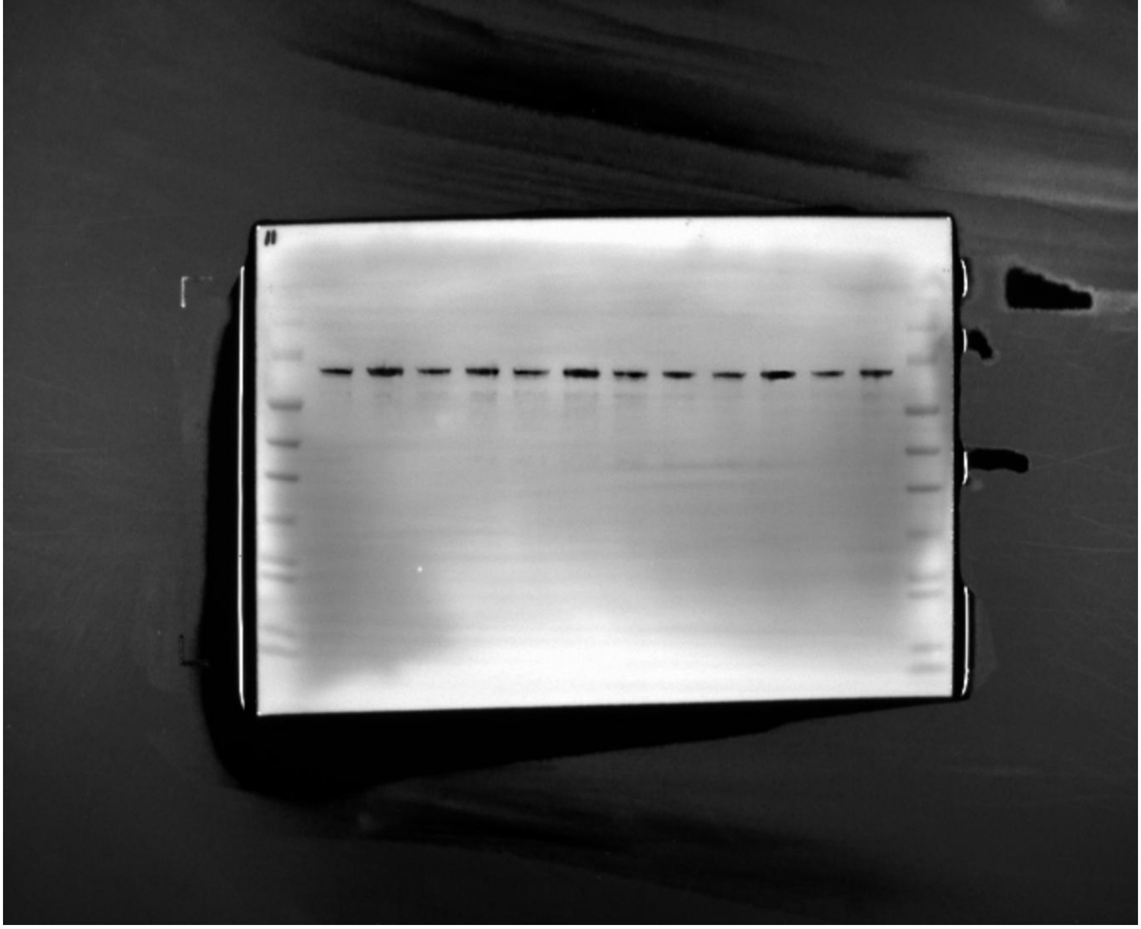

Figure 4E-TBK1

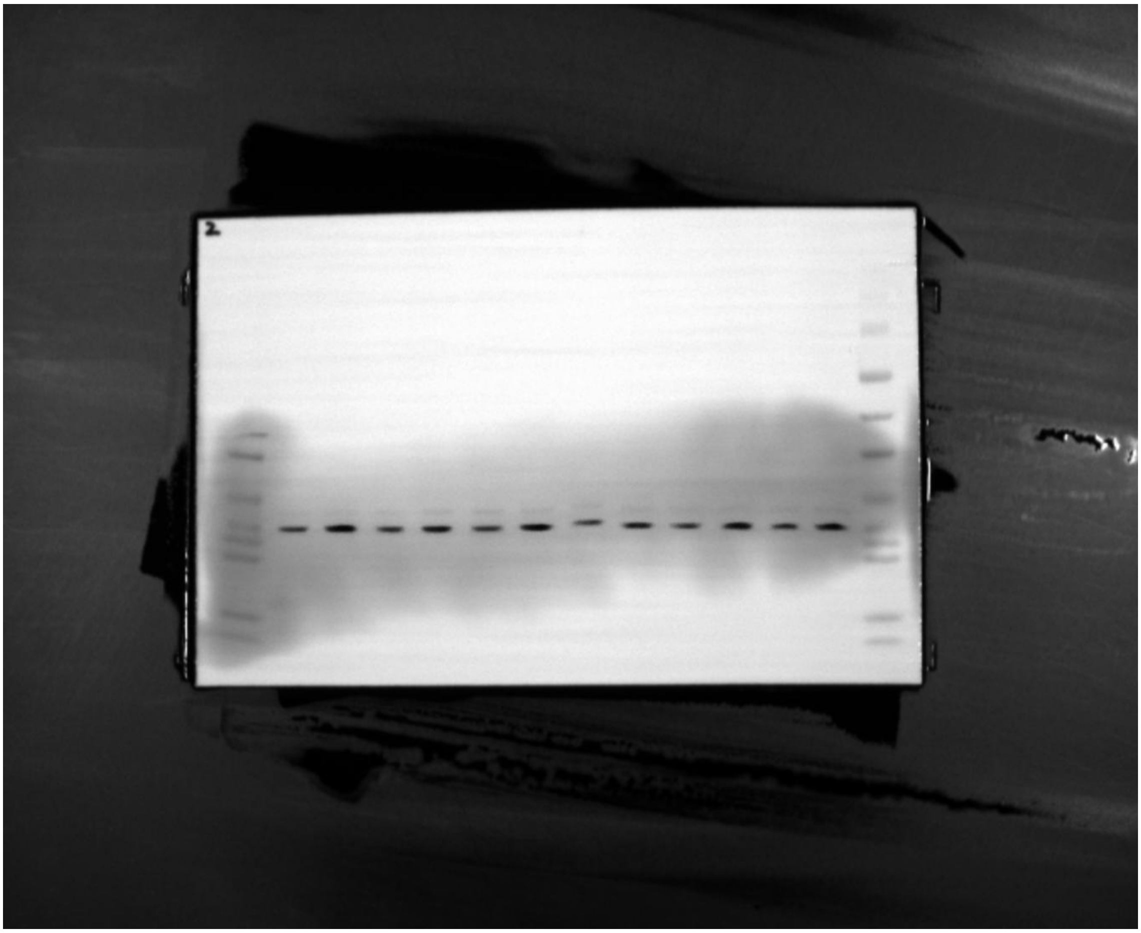

Figure 4E-HMGB1

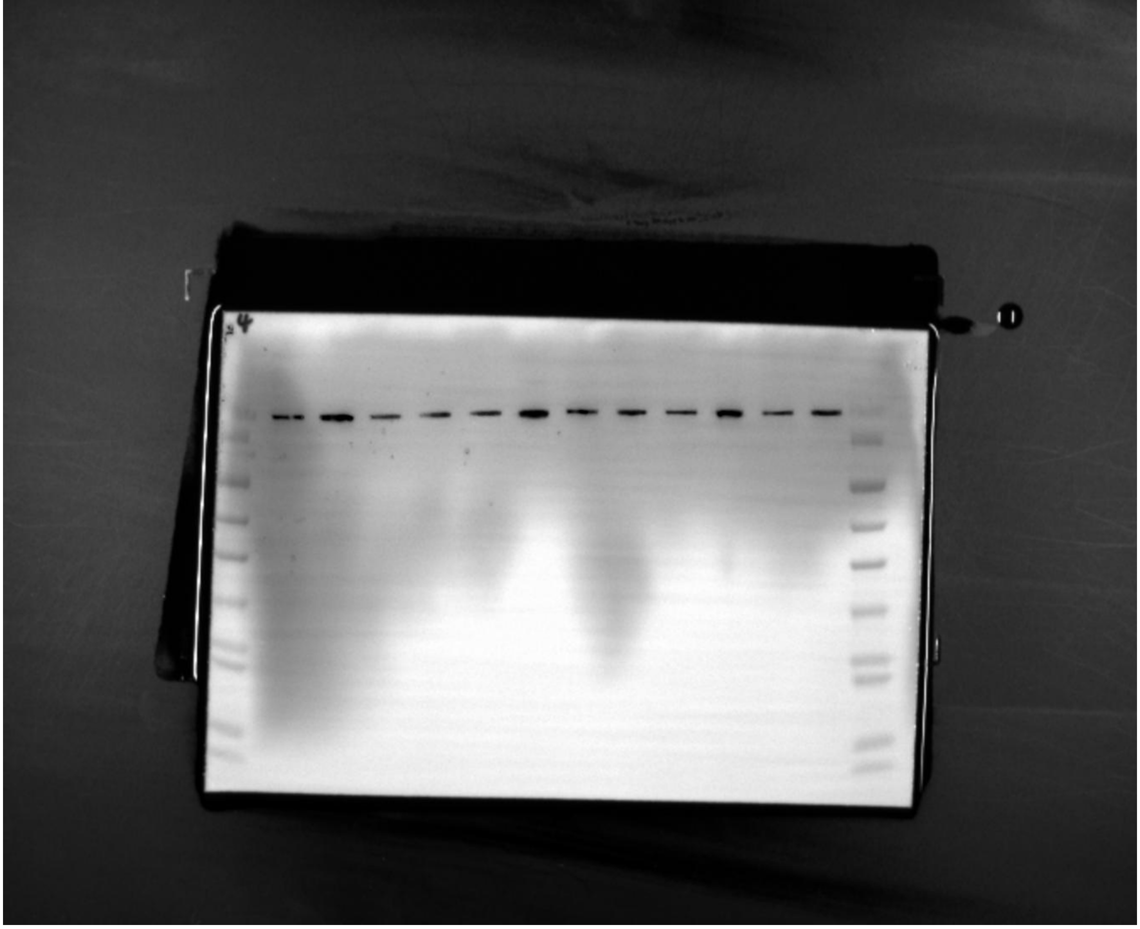

Figure 4E-DAPK

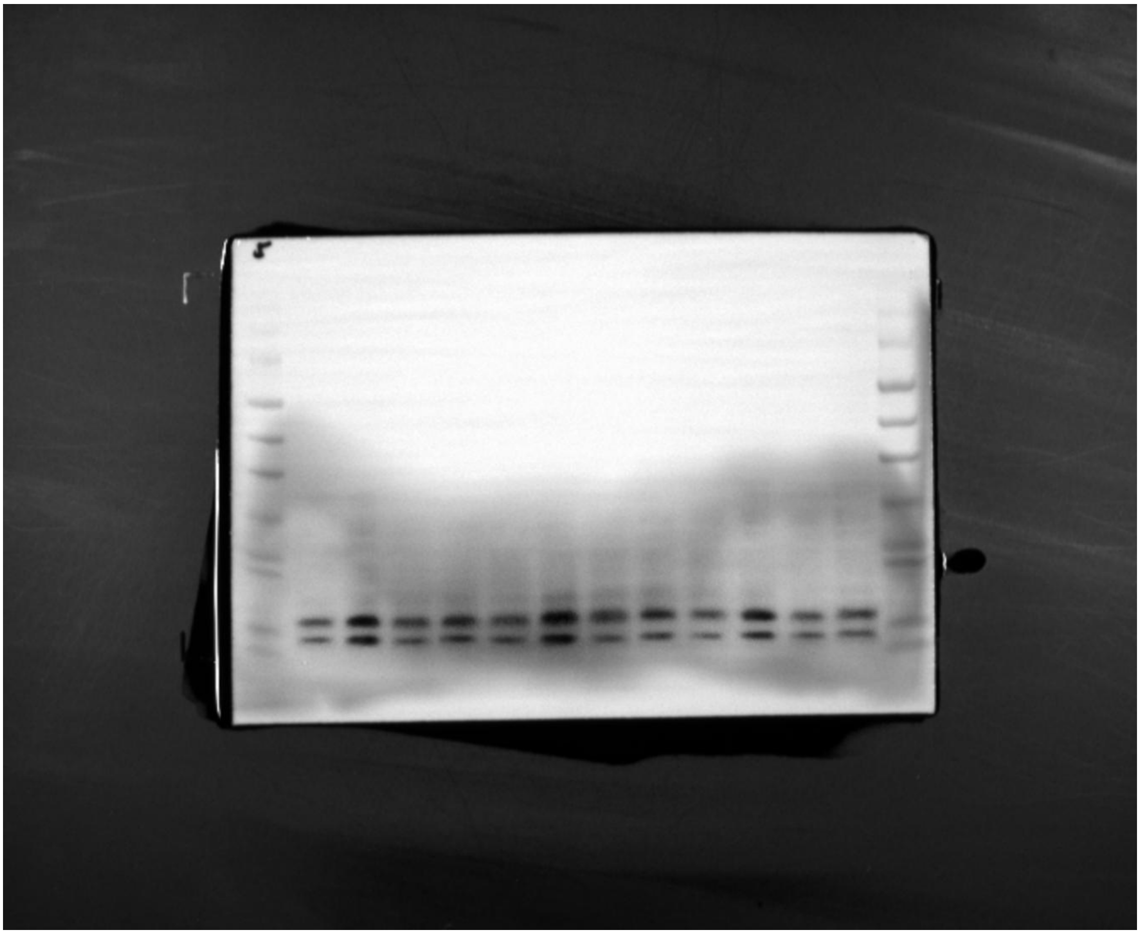

Figure 4E-LC3-I/II

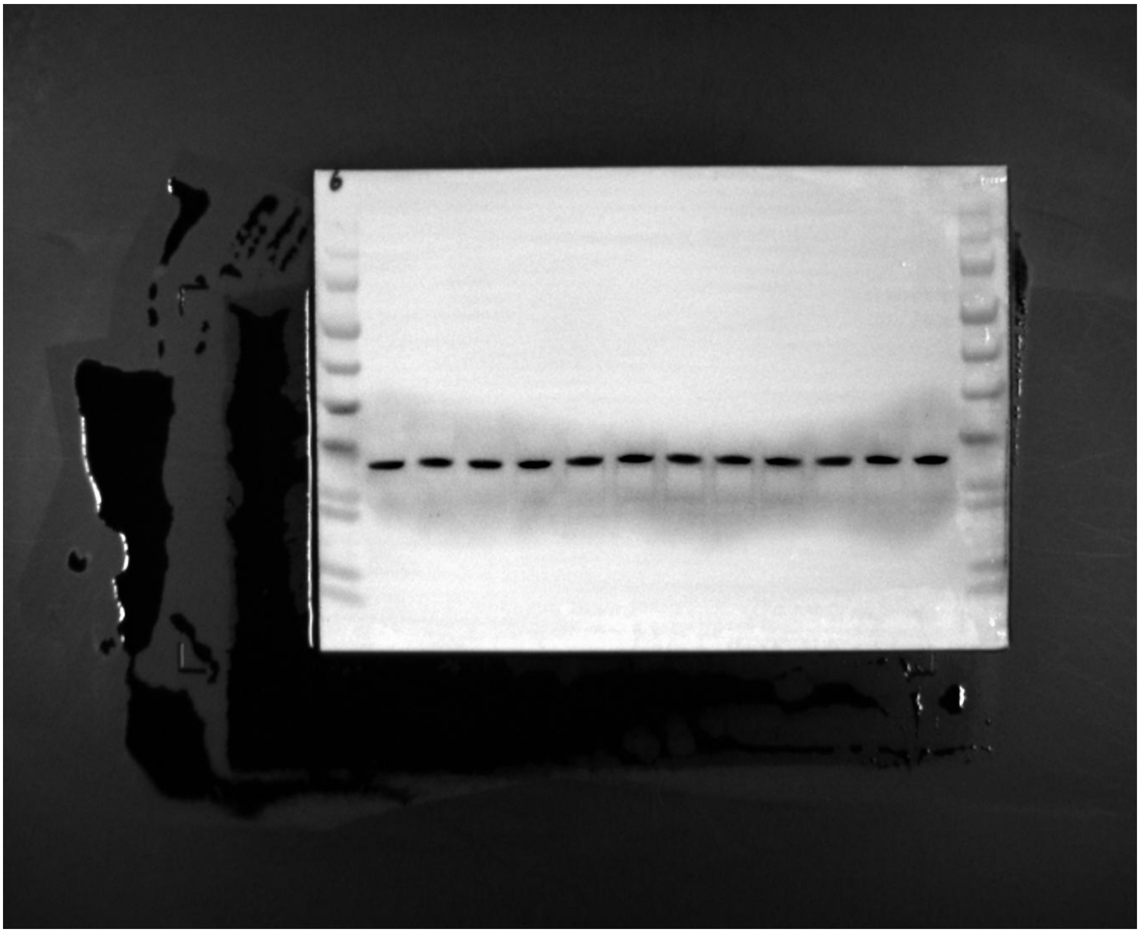

Figure 4E-GAPDH

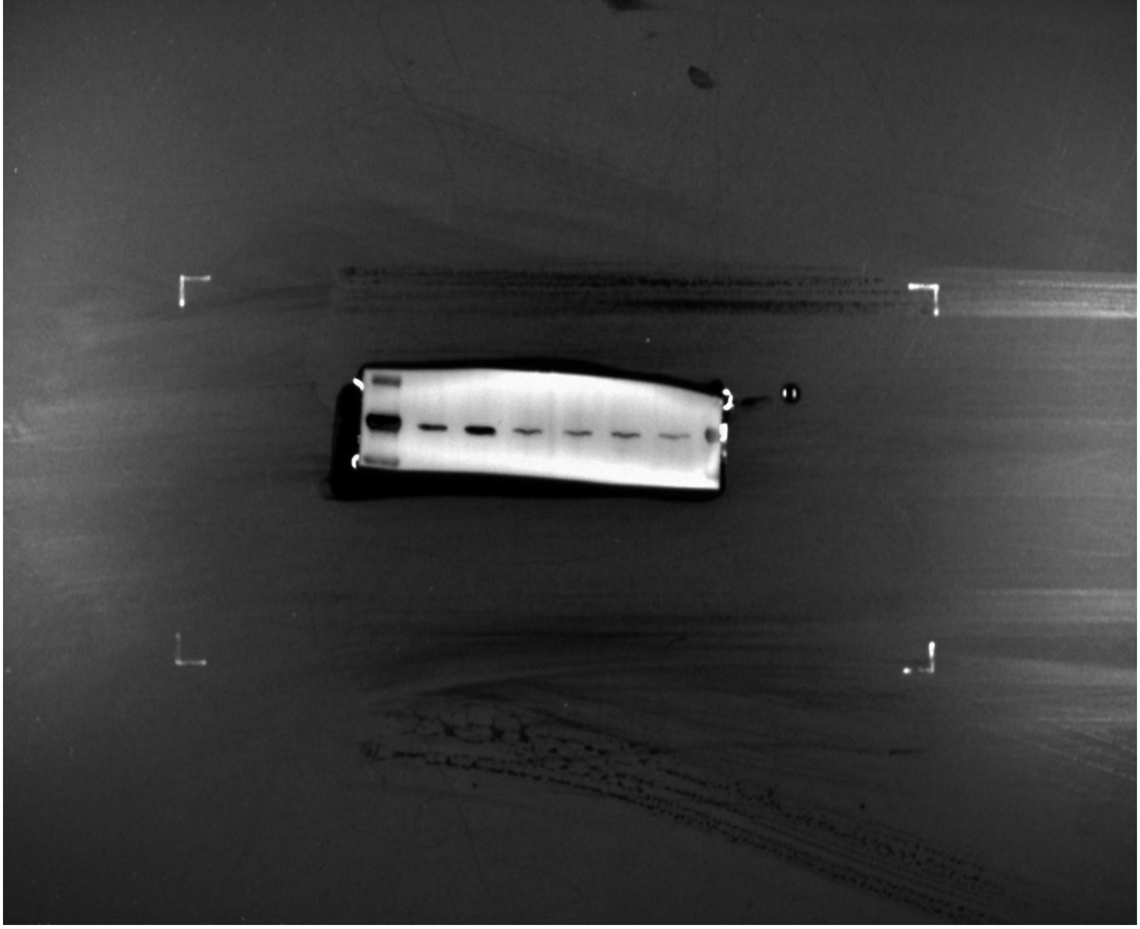

Figure 5C-YAP1

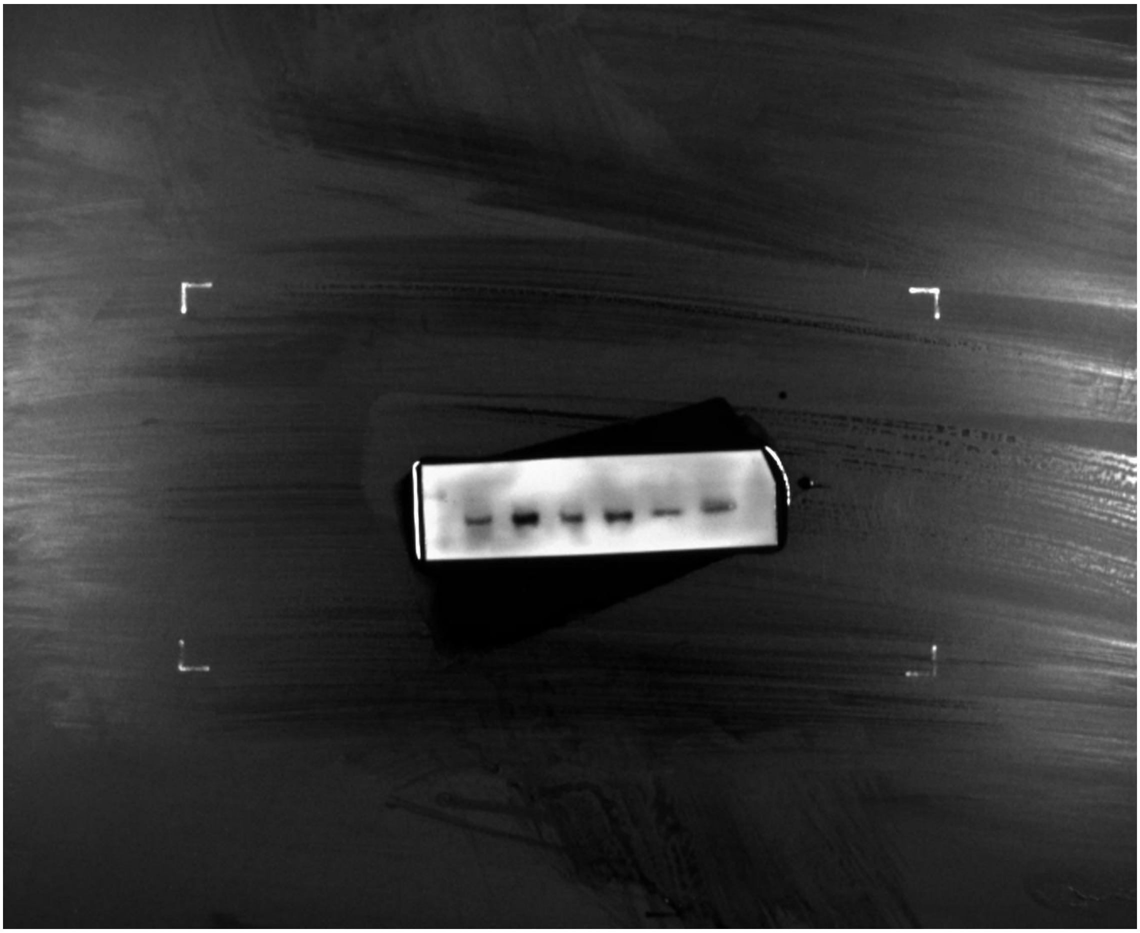

Figure 5C-CTGF

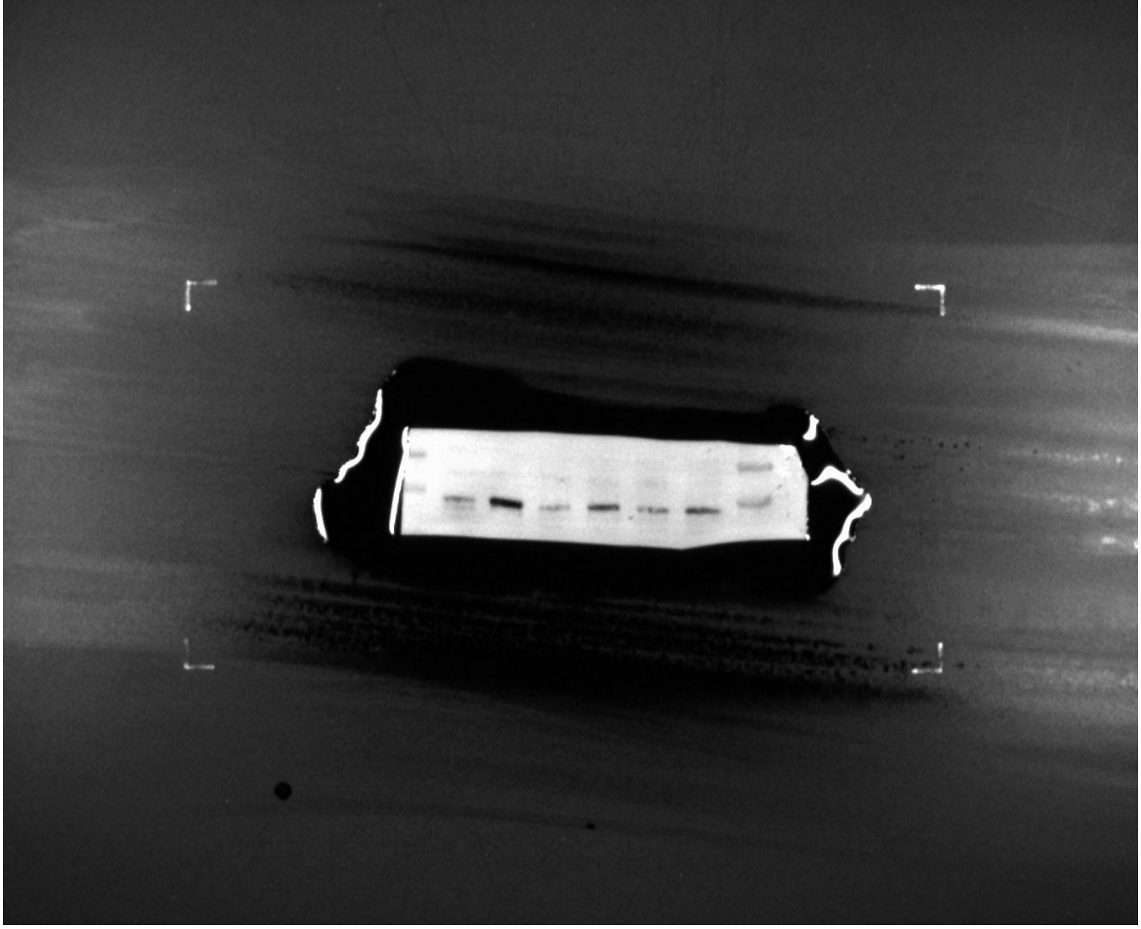

Figure 5C-CYR61

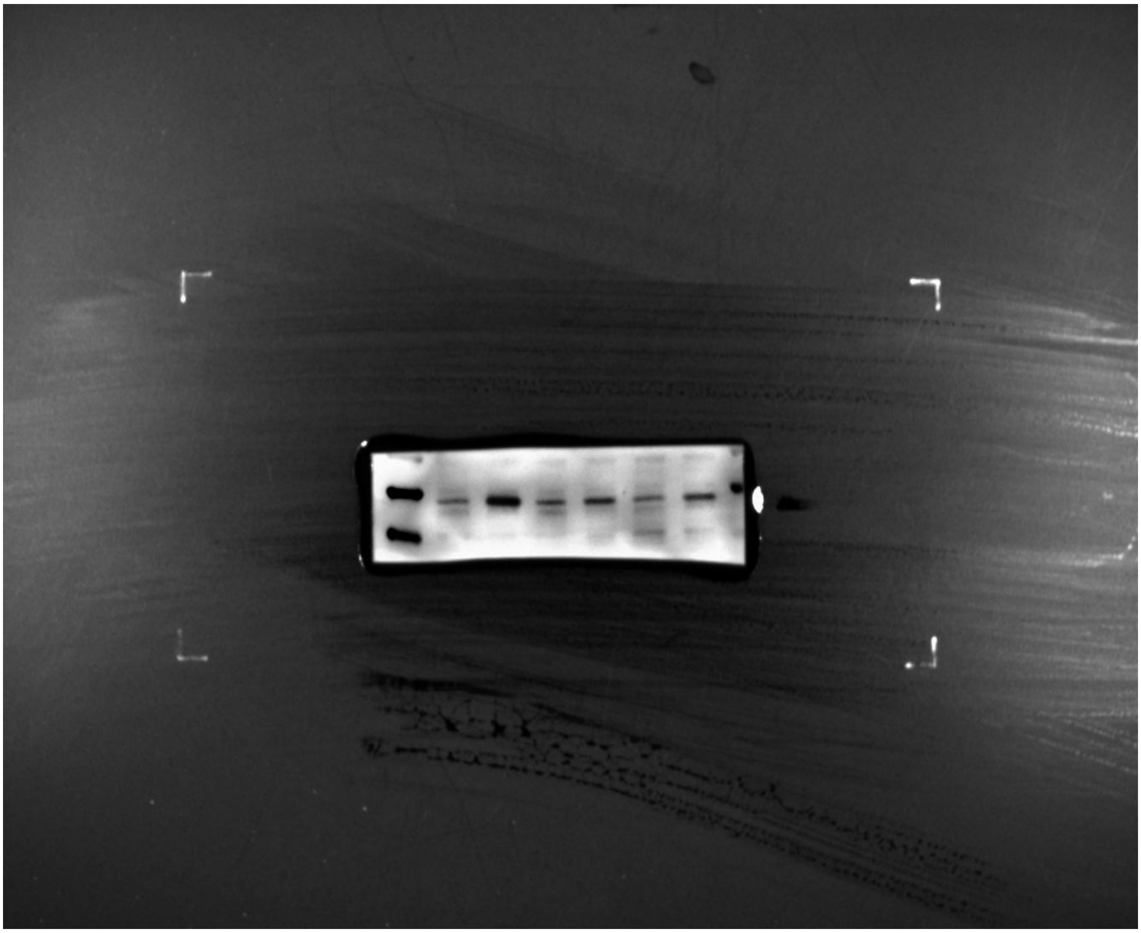

Figure 5C-AREG

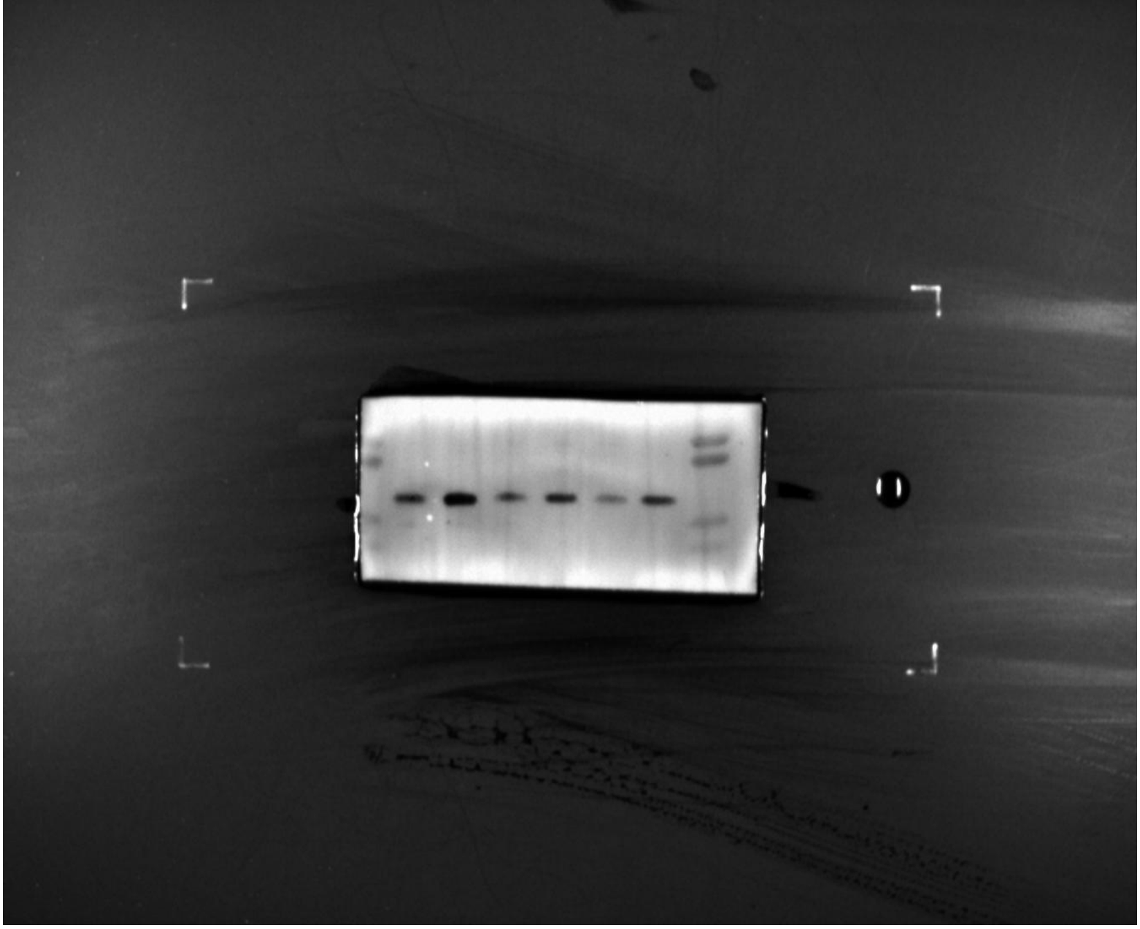

Figure 5C-BIRC5

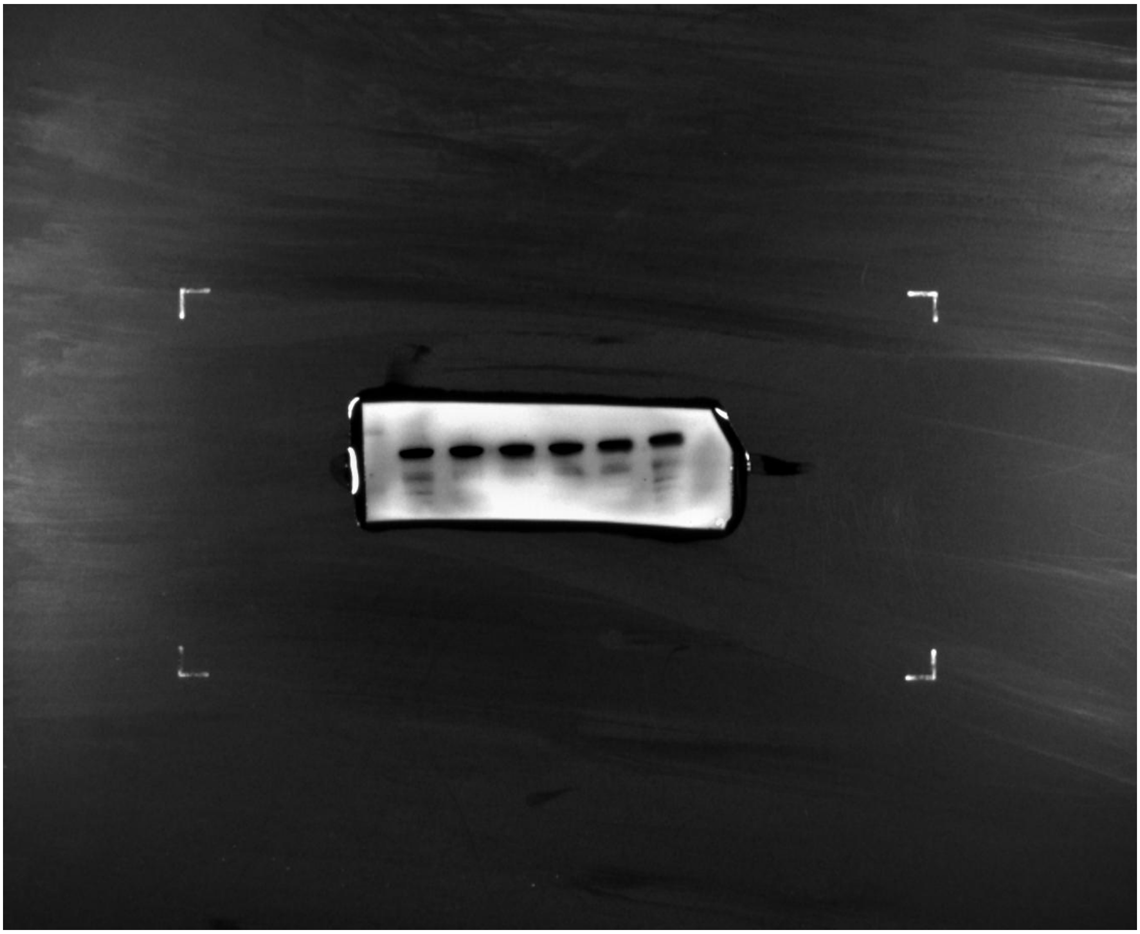

Figure 5C-GAPDH

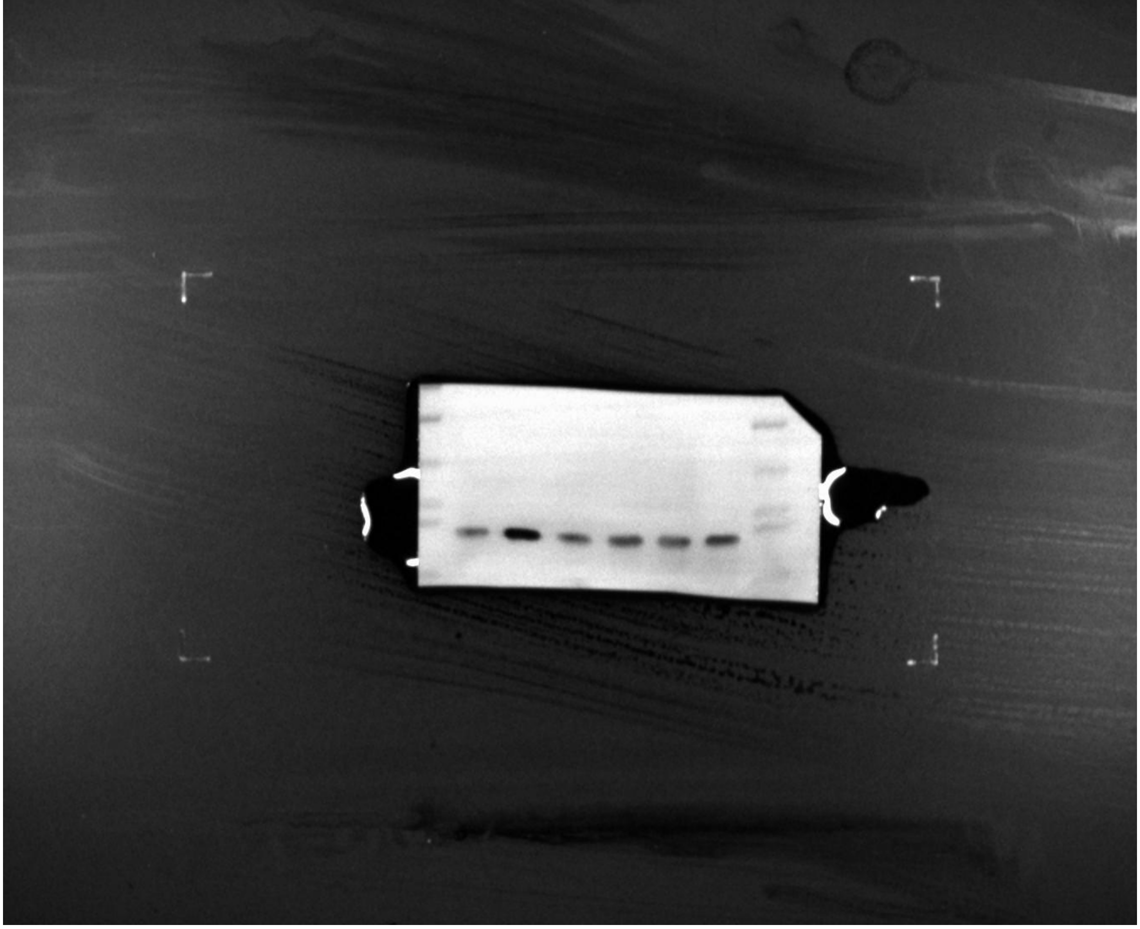

Figure 5E-FUND1

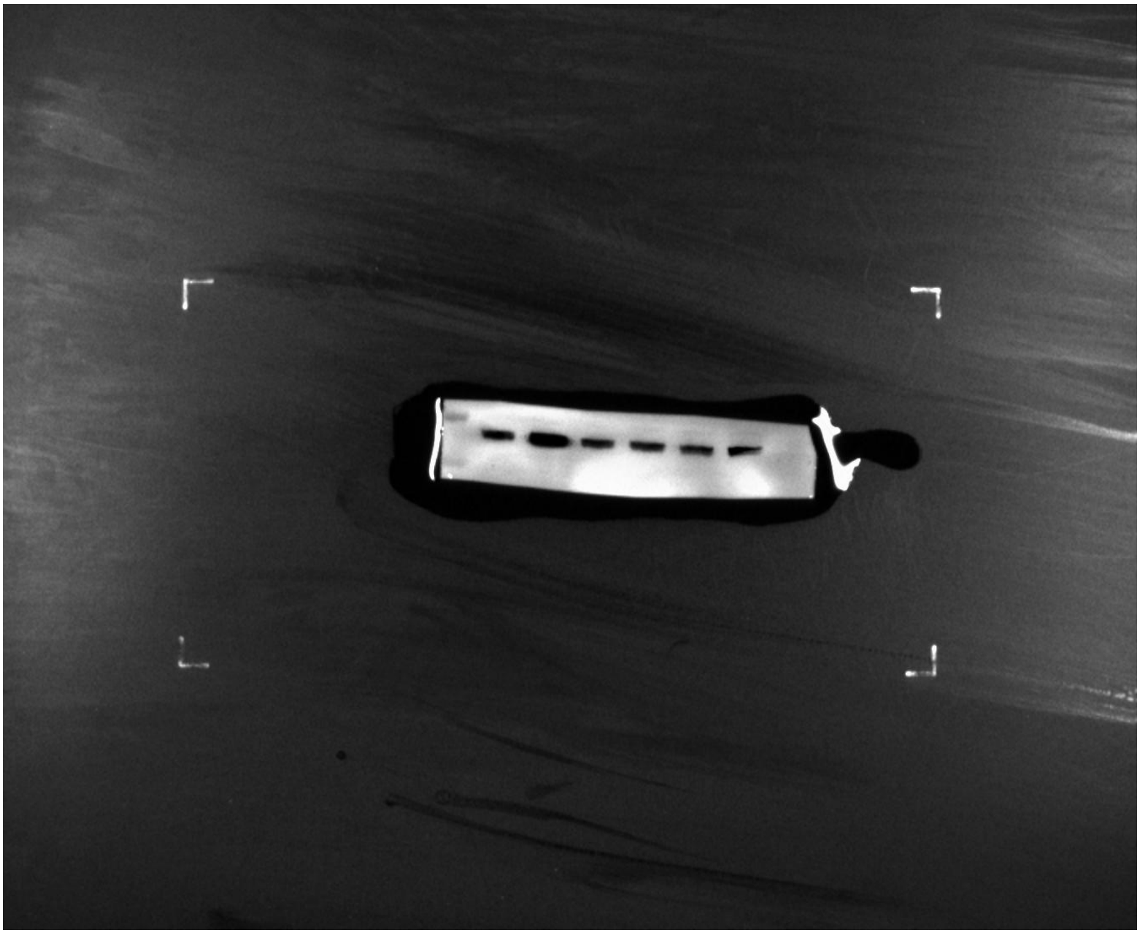

Figure 5E-PINK1

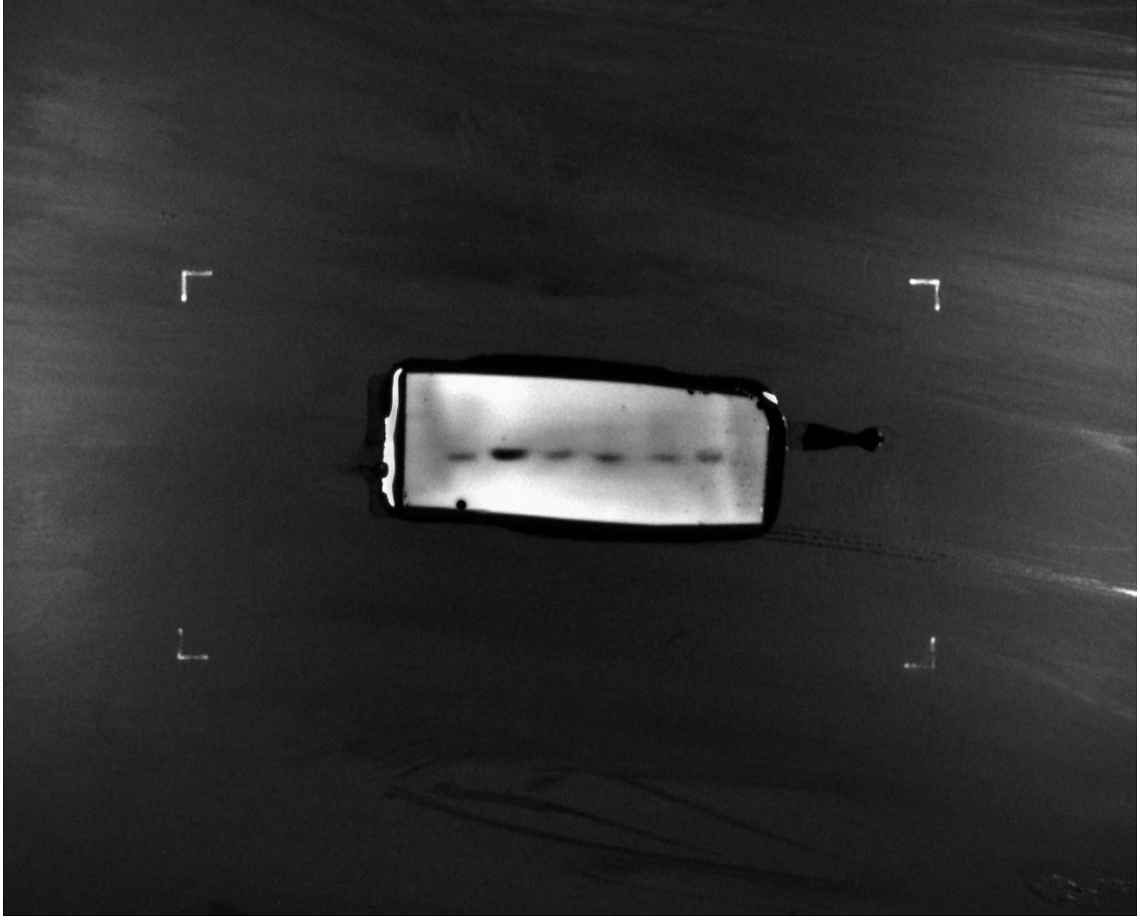

Figure 5E-TBK1

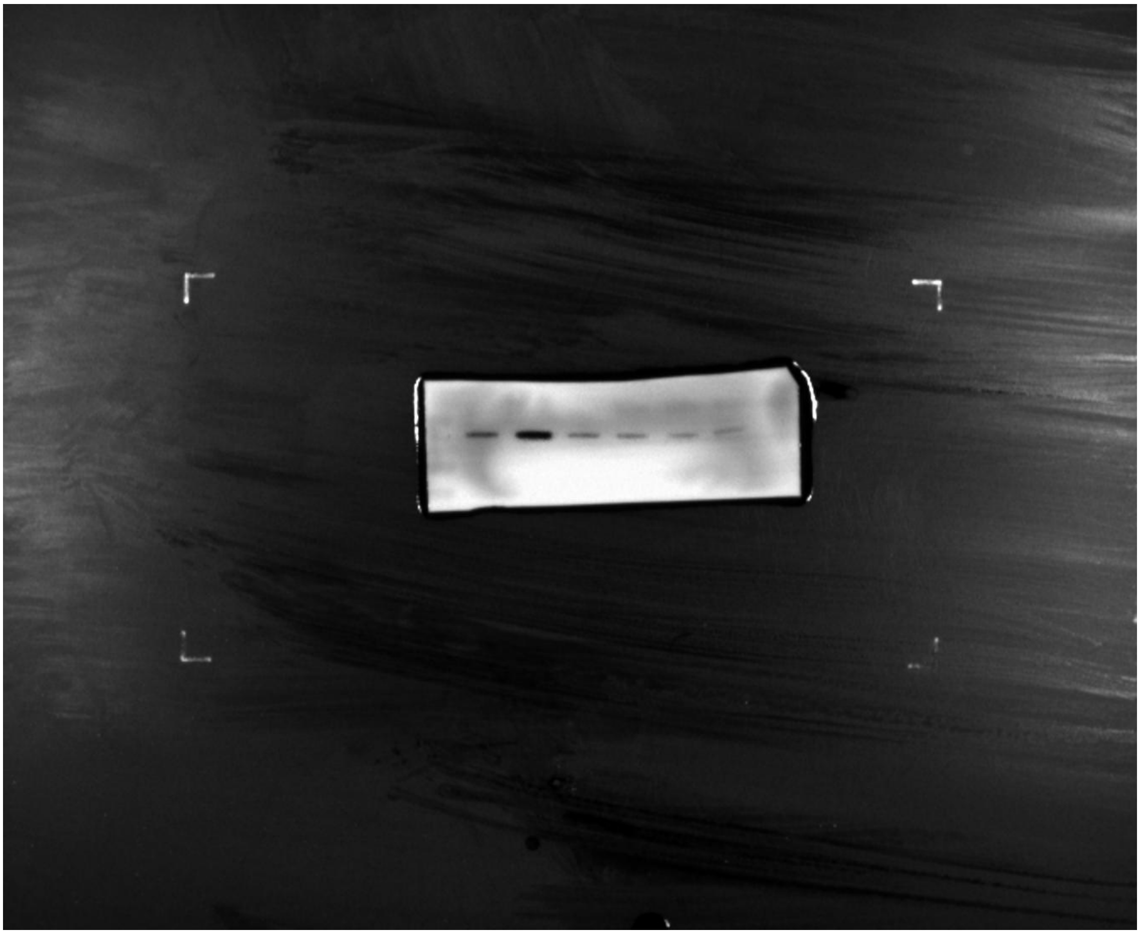

Figure 5E-HMGB1

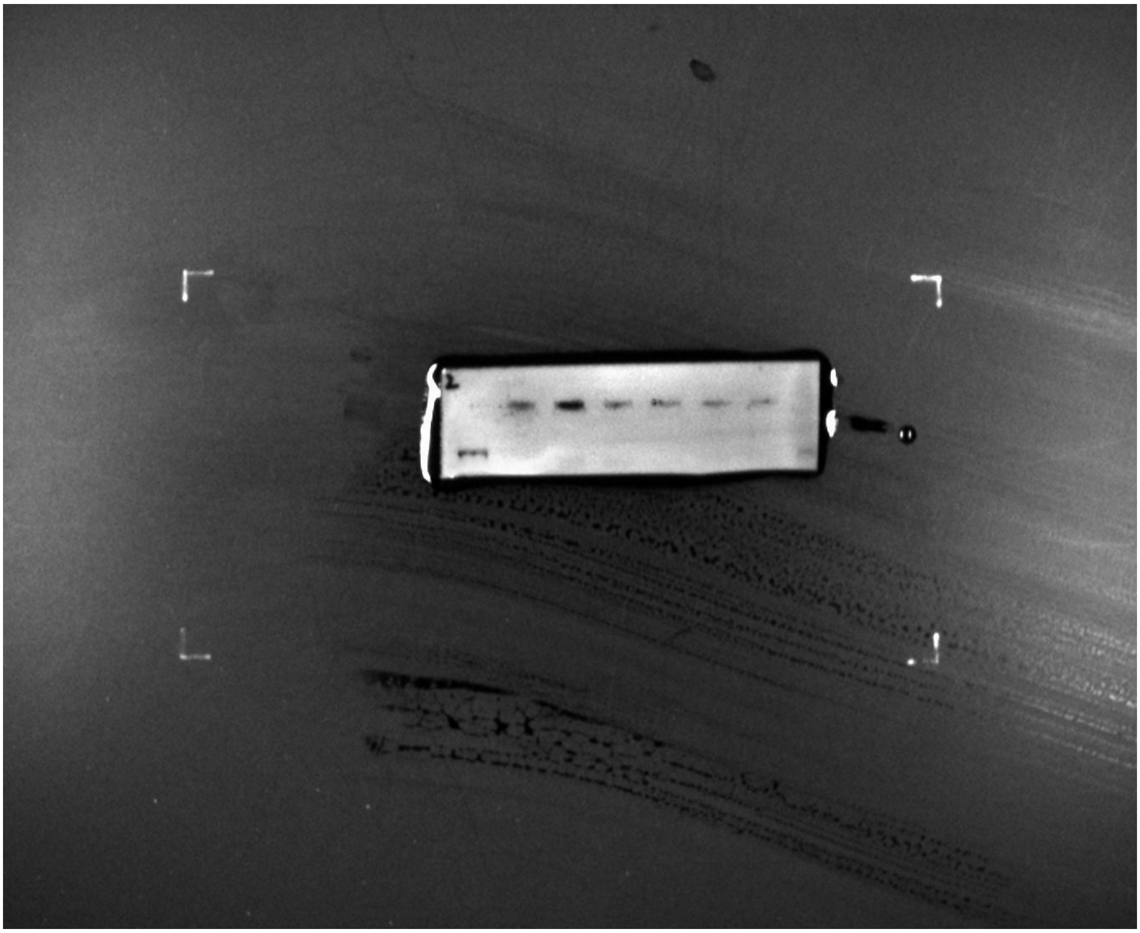

Figure 5E-DAPK

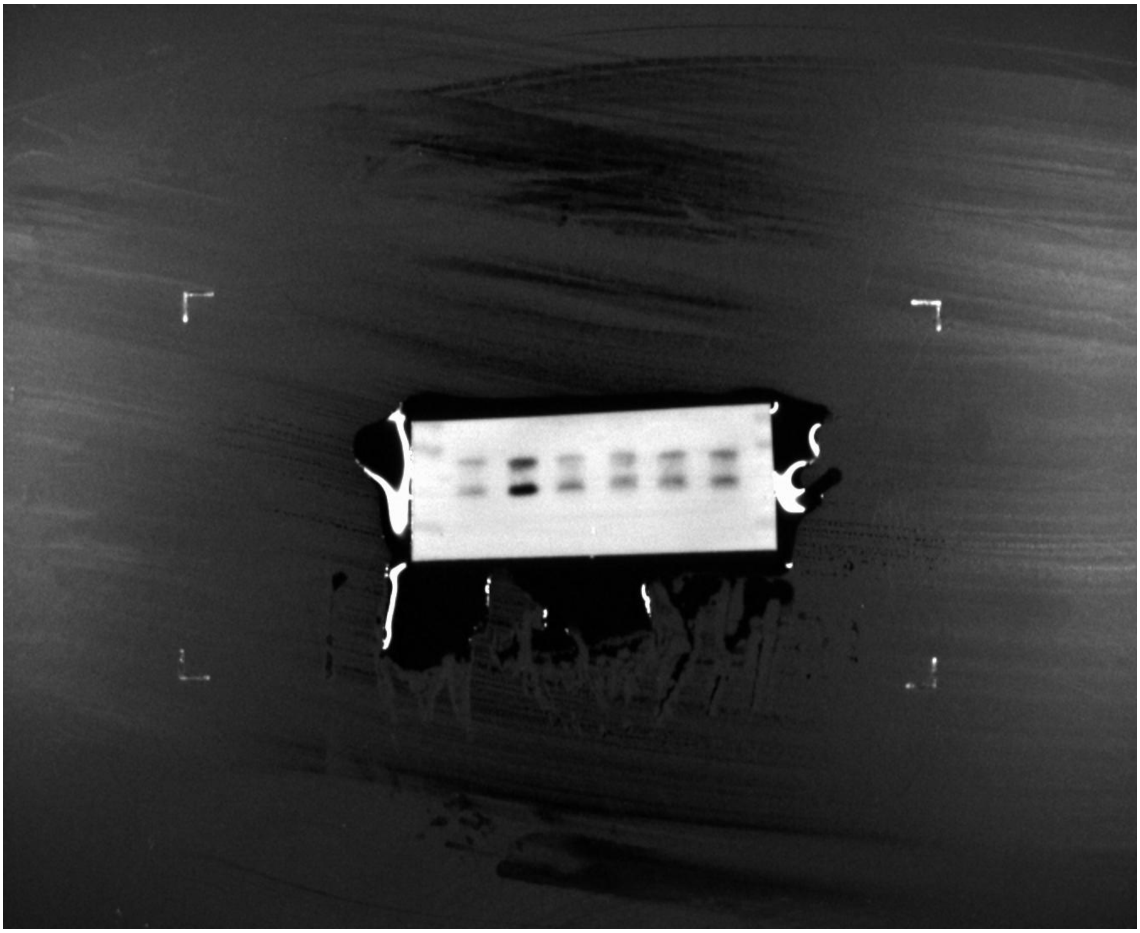

Figure 5E-LC3-I/II

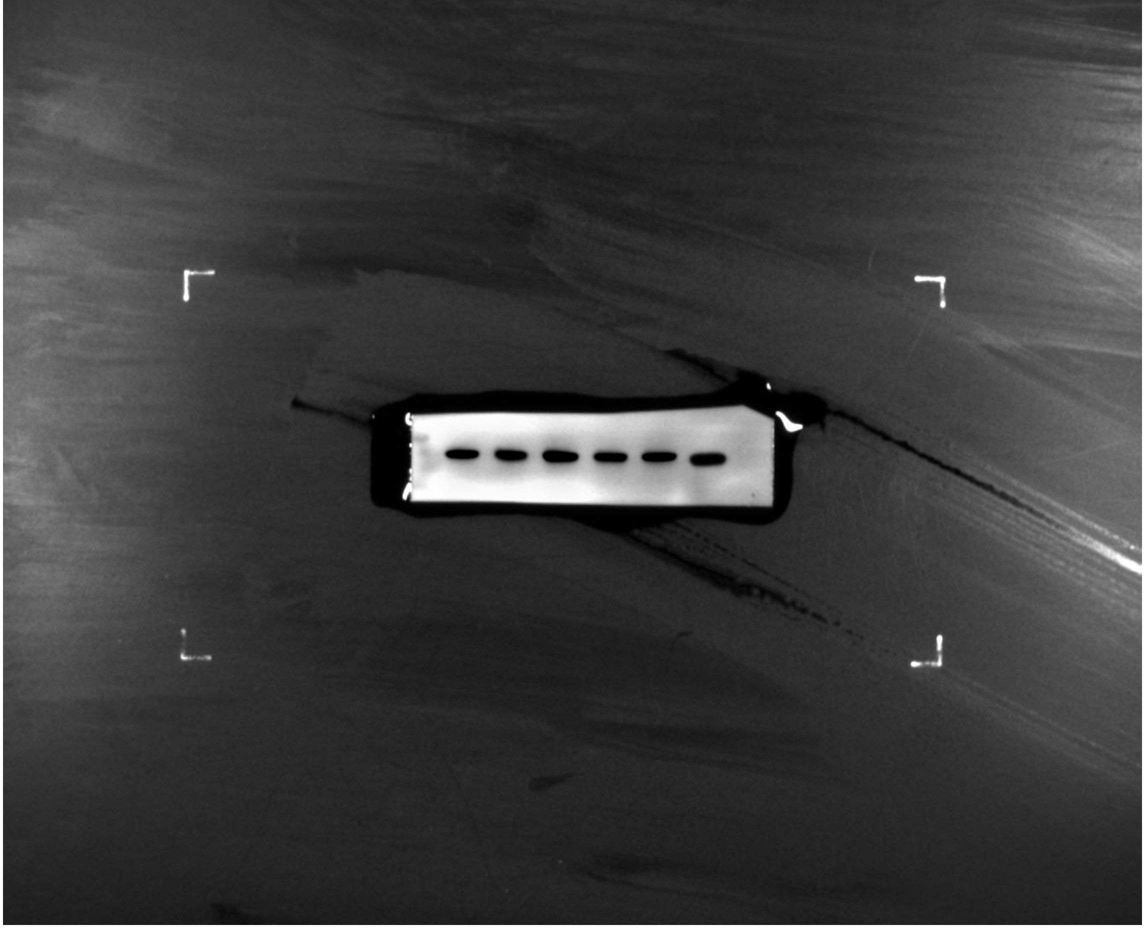

Figure 5E-GAPDH

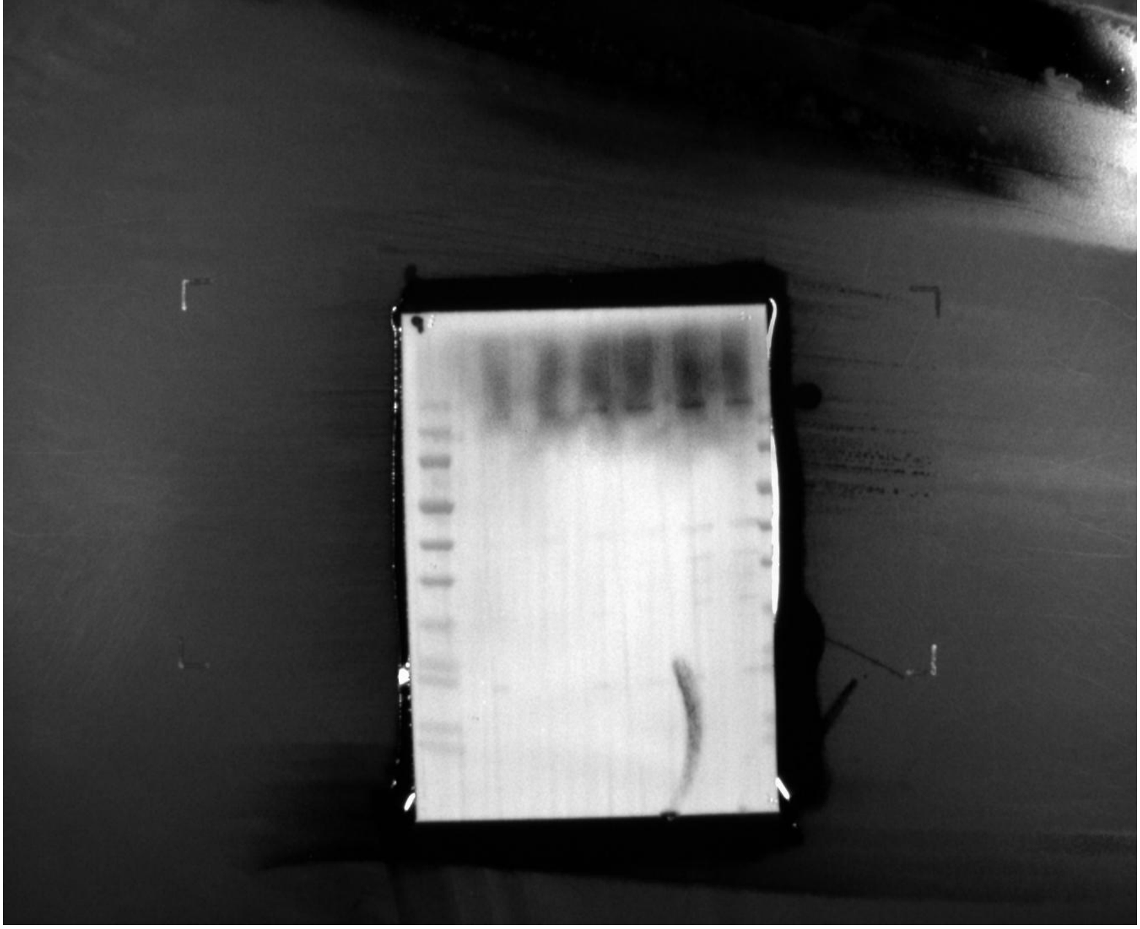

Figure 6A-O-GlcNAc

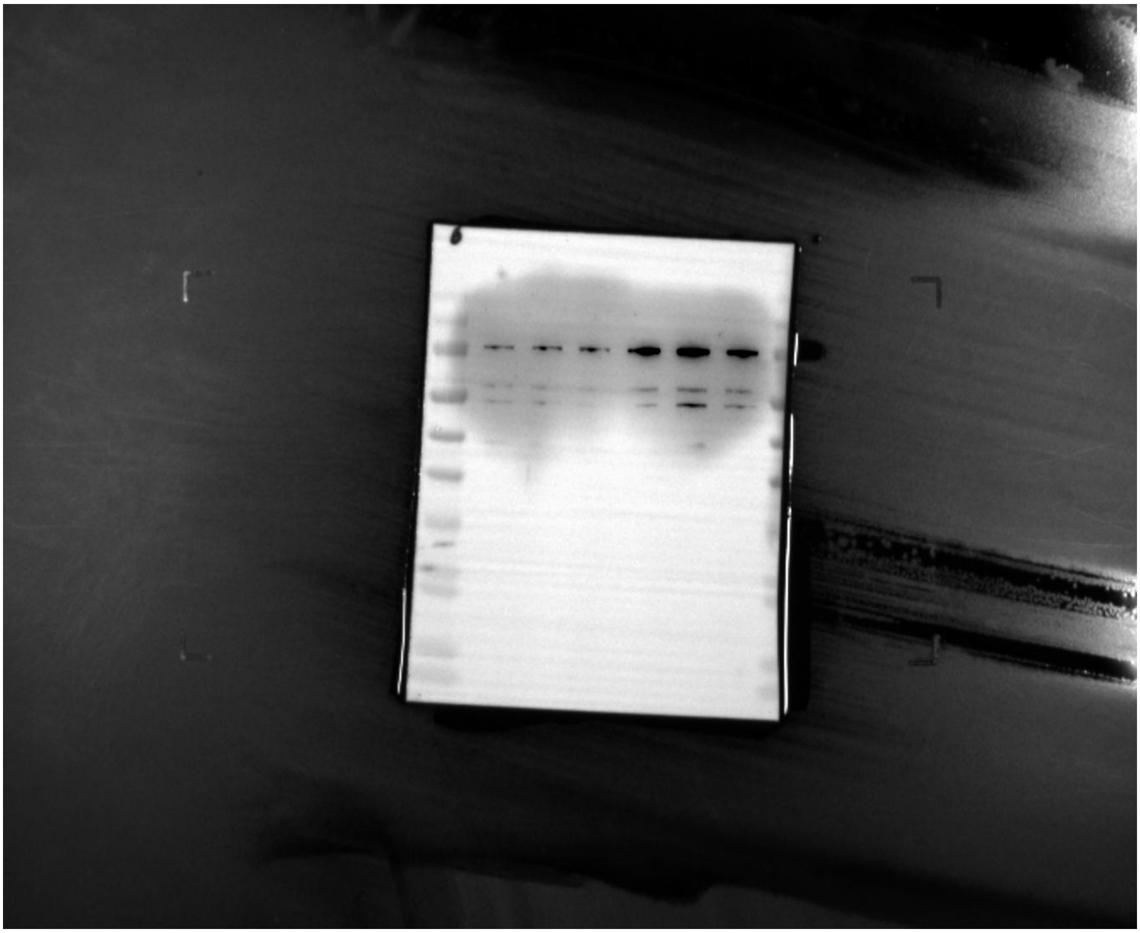

Figure 6A-OGT

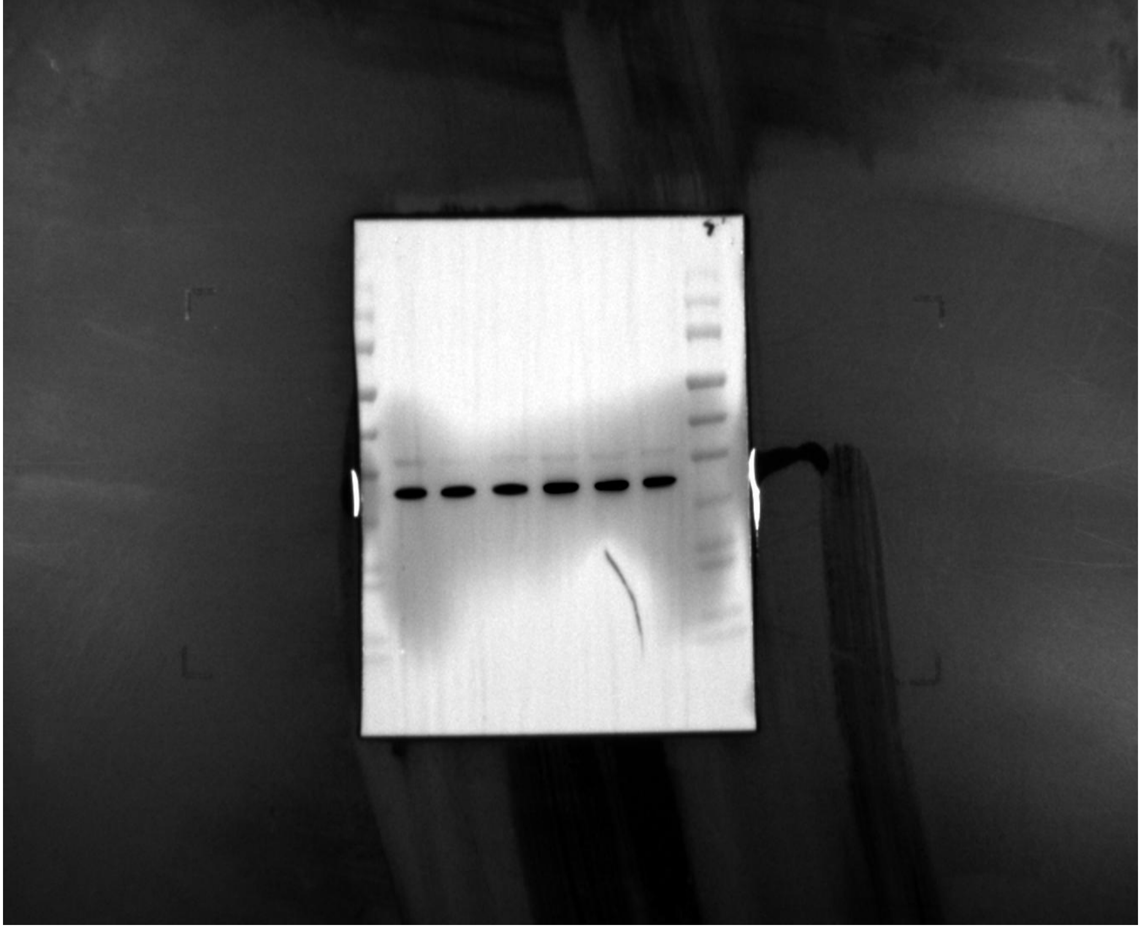

Figure 6A-GAPDH

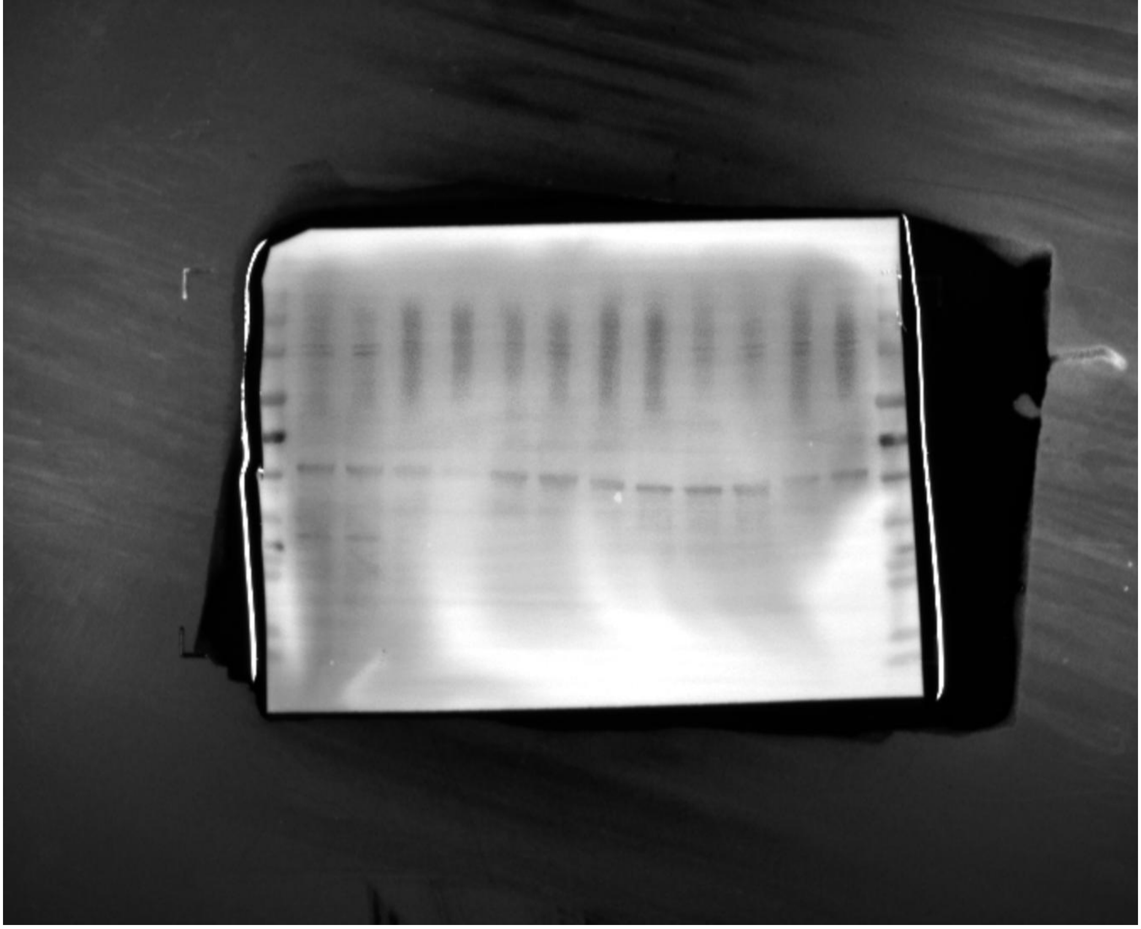

Figure 6B-O-GlcNAc

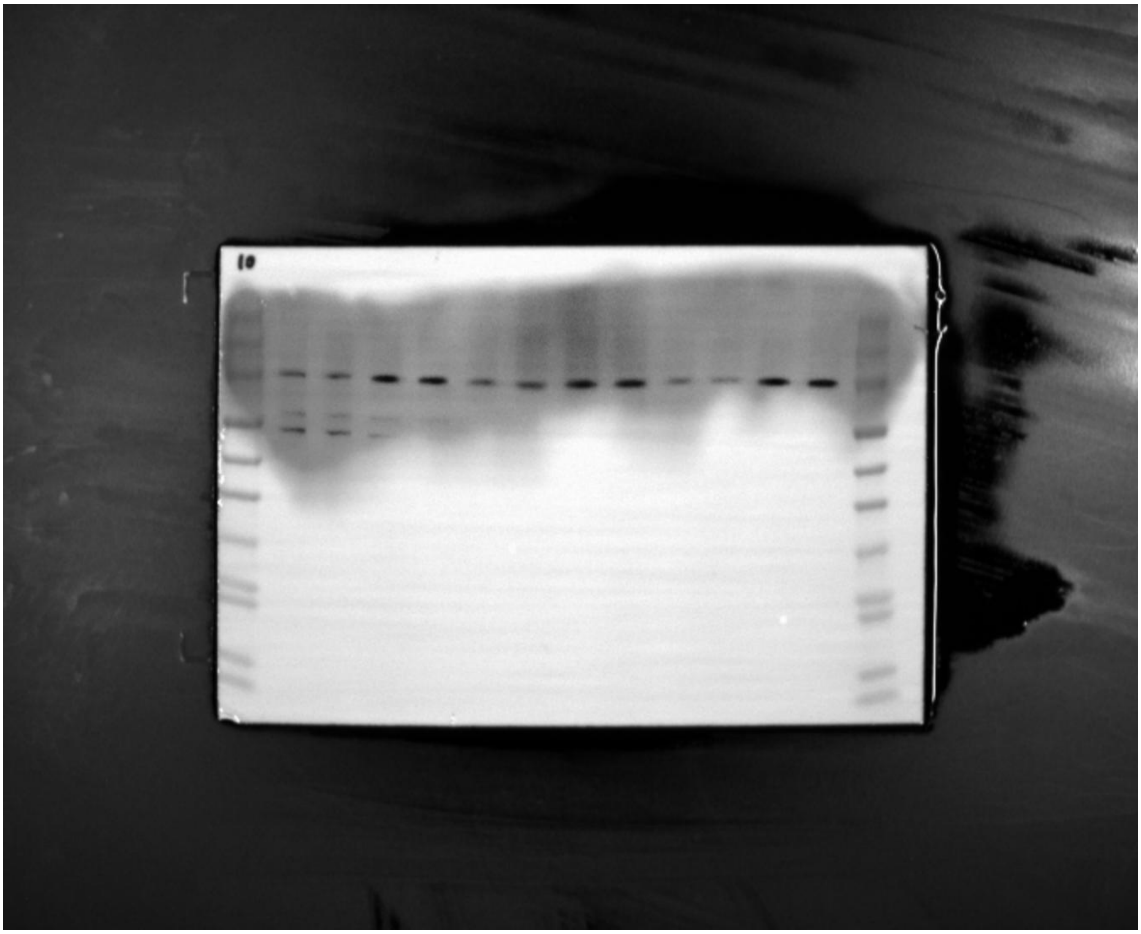

Figure 6B-OGT

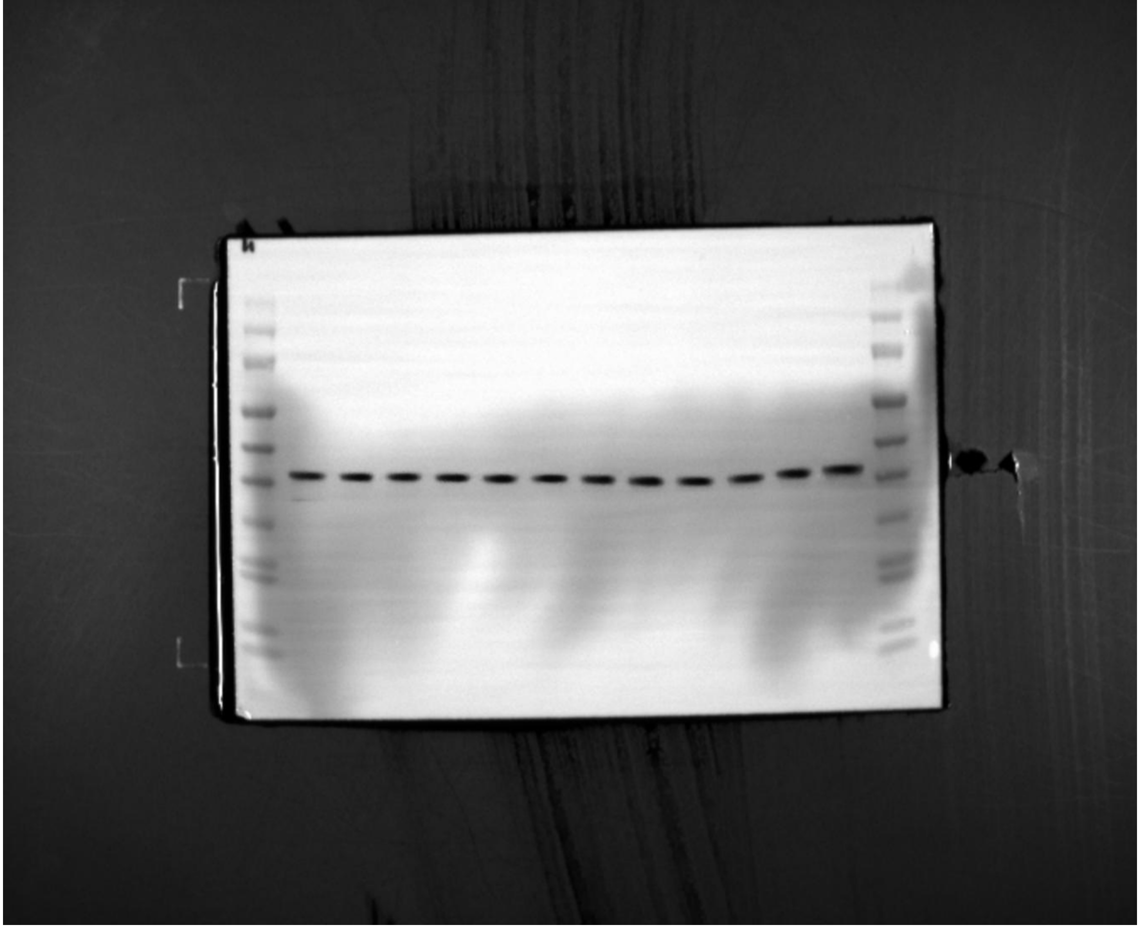

Figure 6B-GAPDH

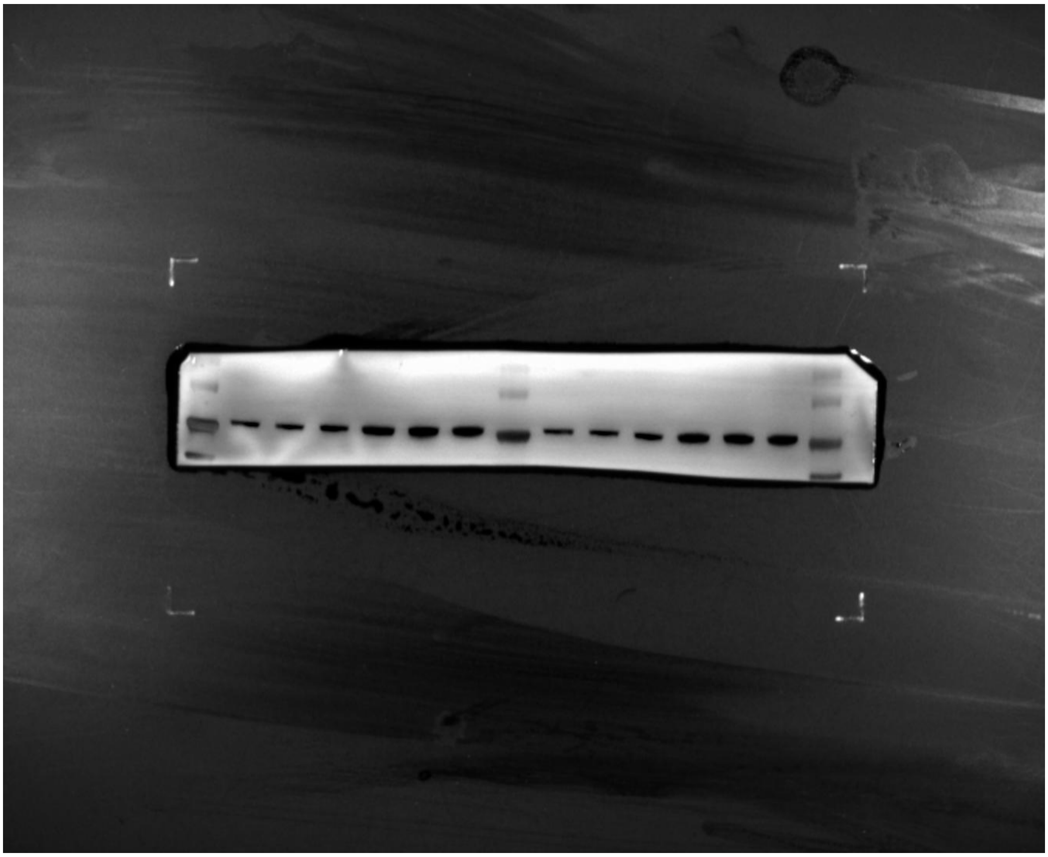

Figure 6C-YAP1-Input

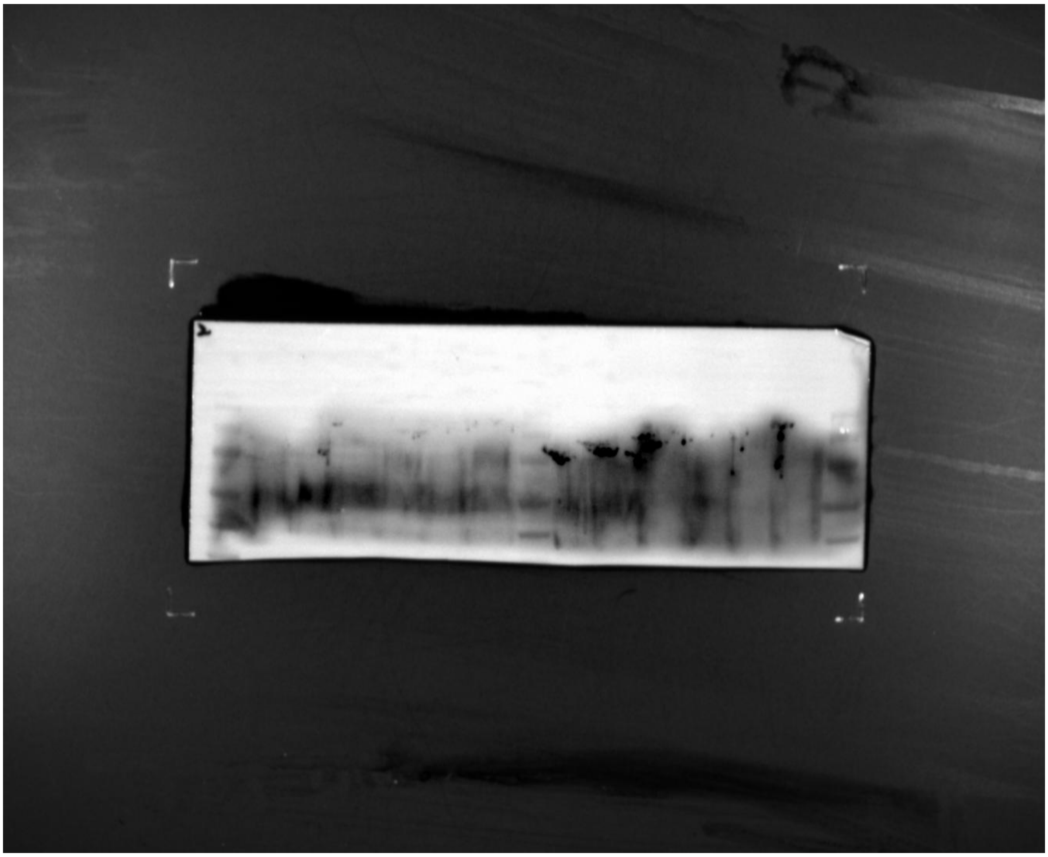

Figure 6C-O-GlcNAc-YAP1-IgG IP

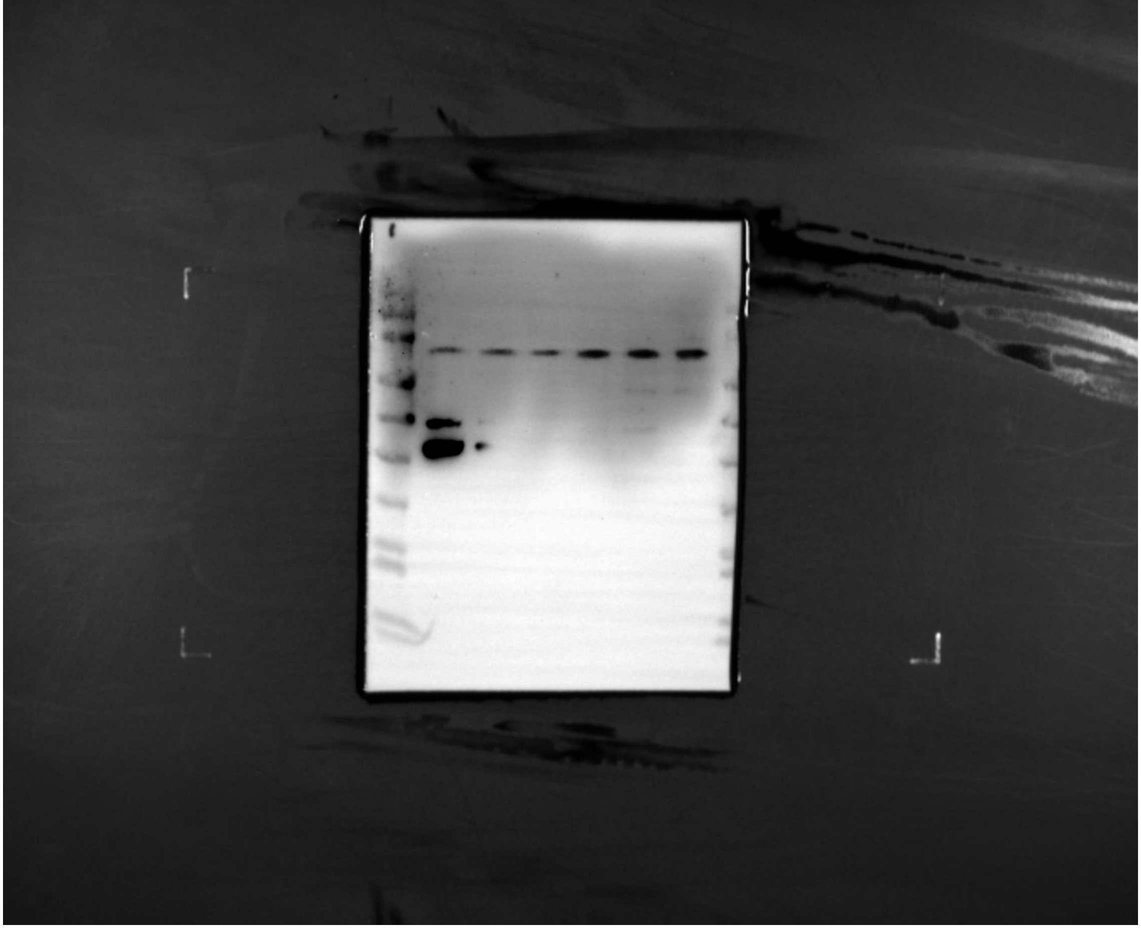

Figure 6C-O-GlcNAc-YAP1

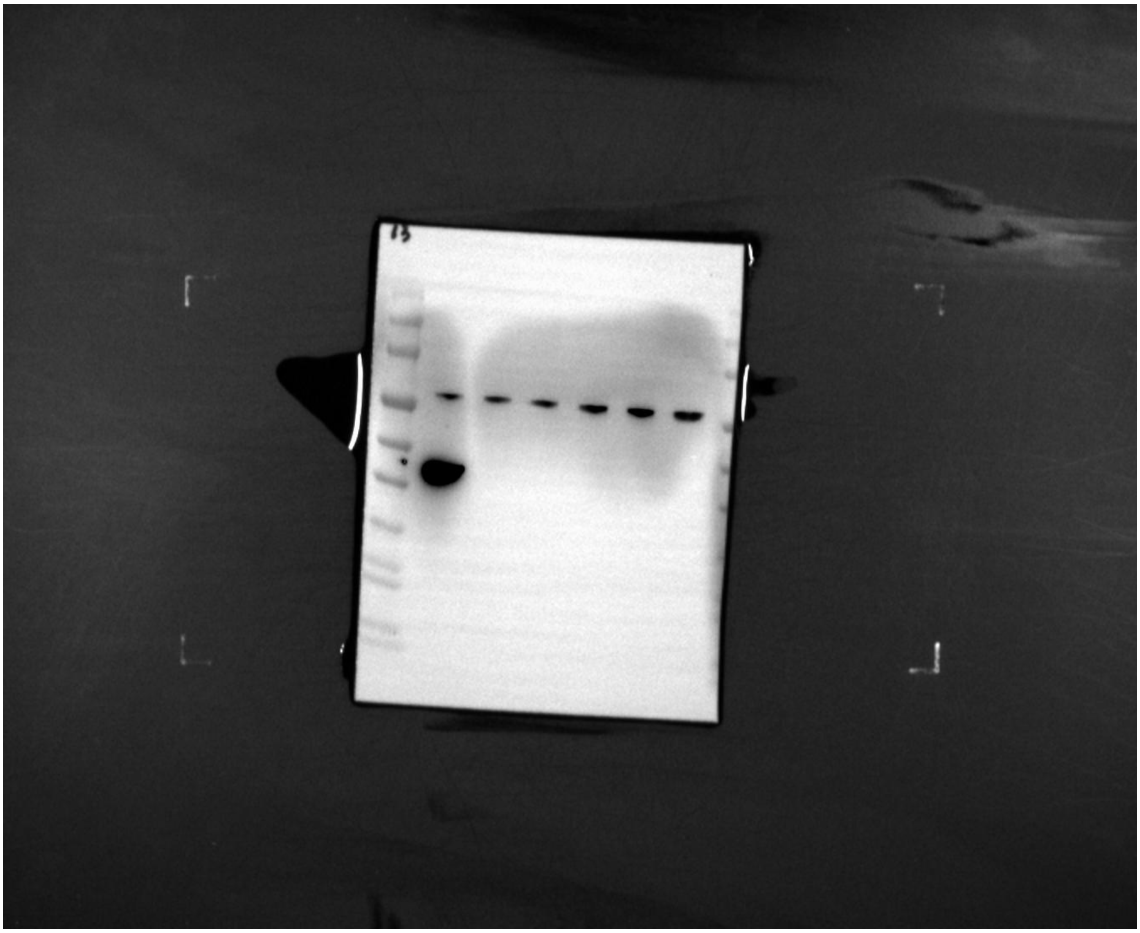

Figure 6C-HIF1A

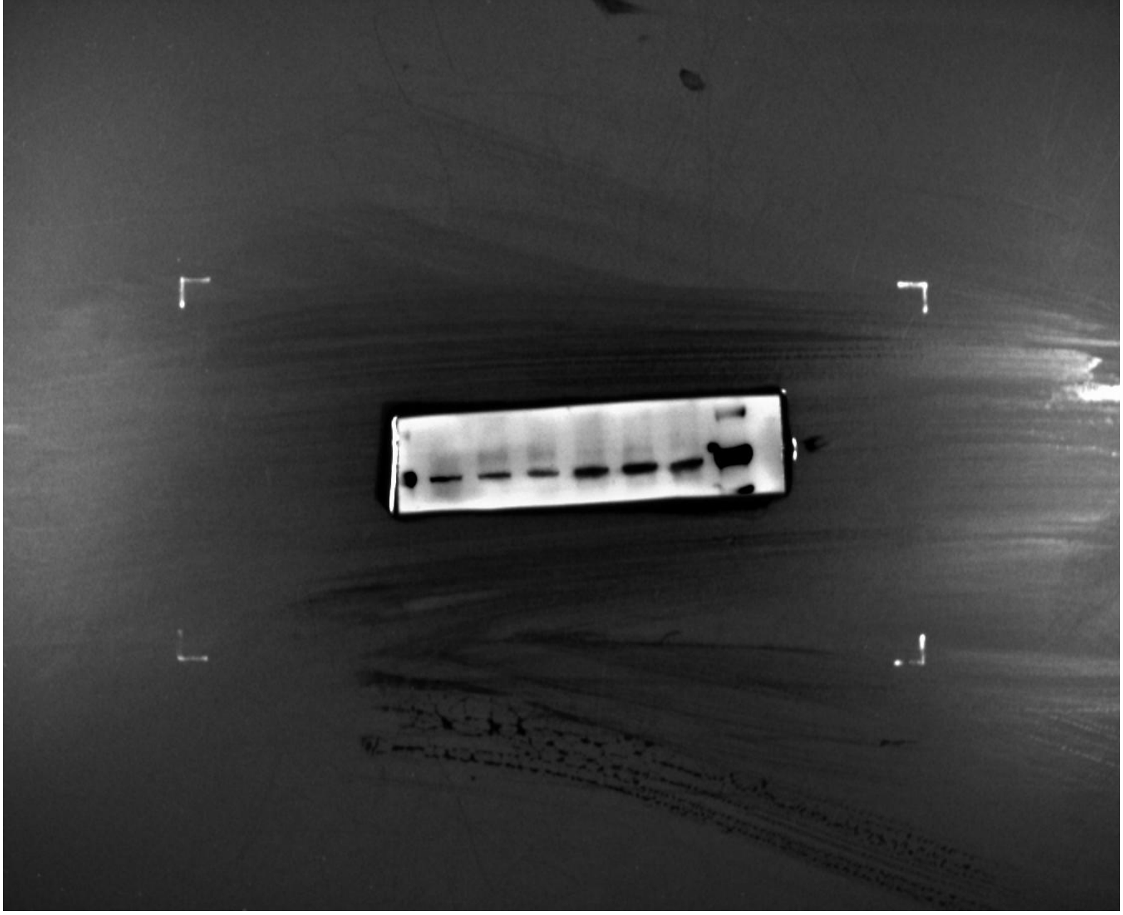

Figure 6C-YAP1 IB

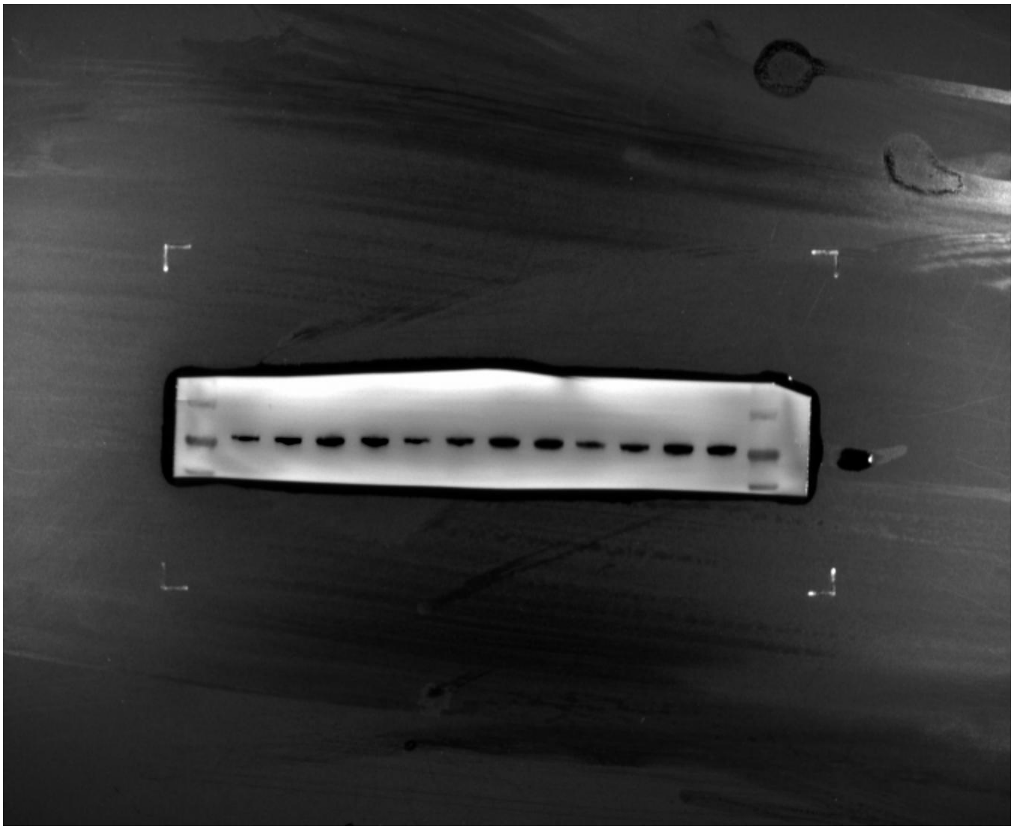

Figure 6D-Input-YAP1

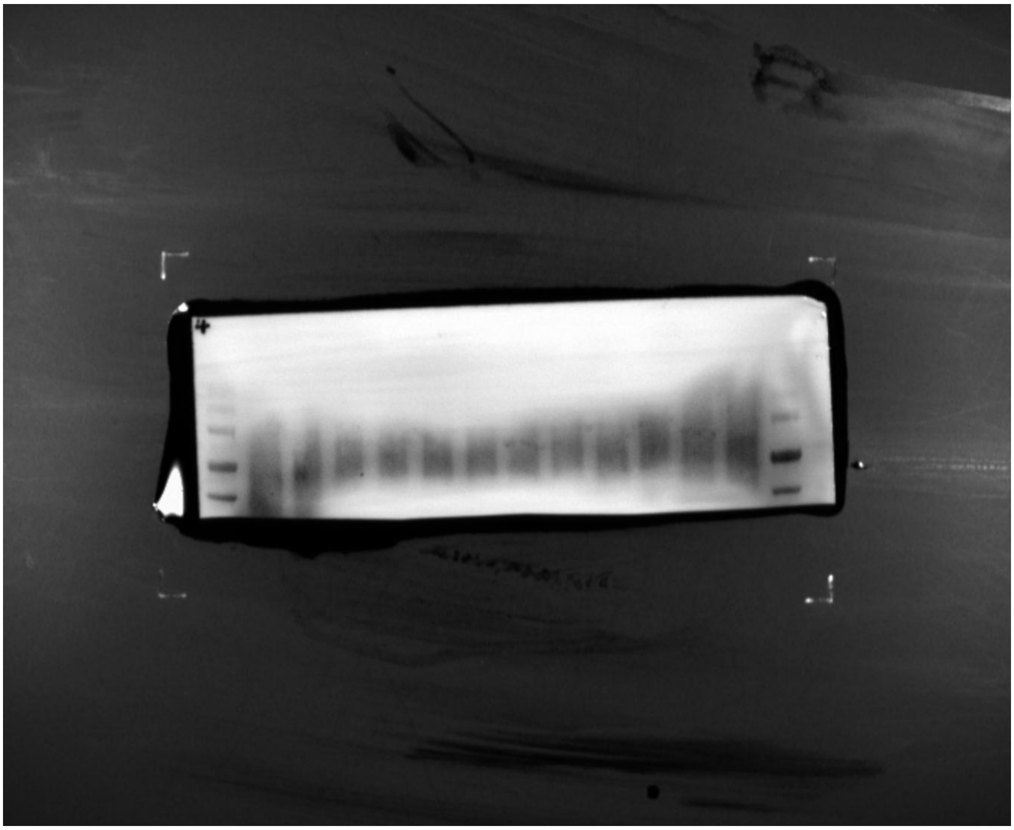

Figure 6D-O-GlcNAc-YAP1-IgG IP

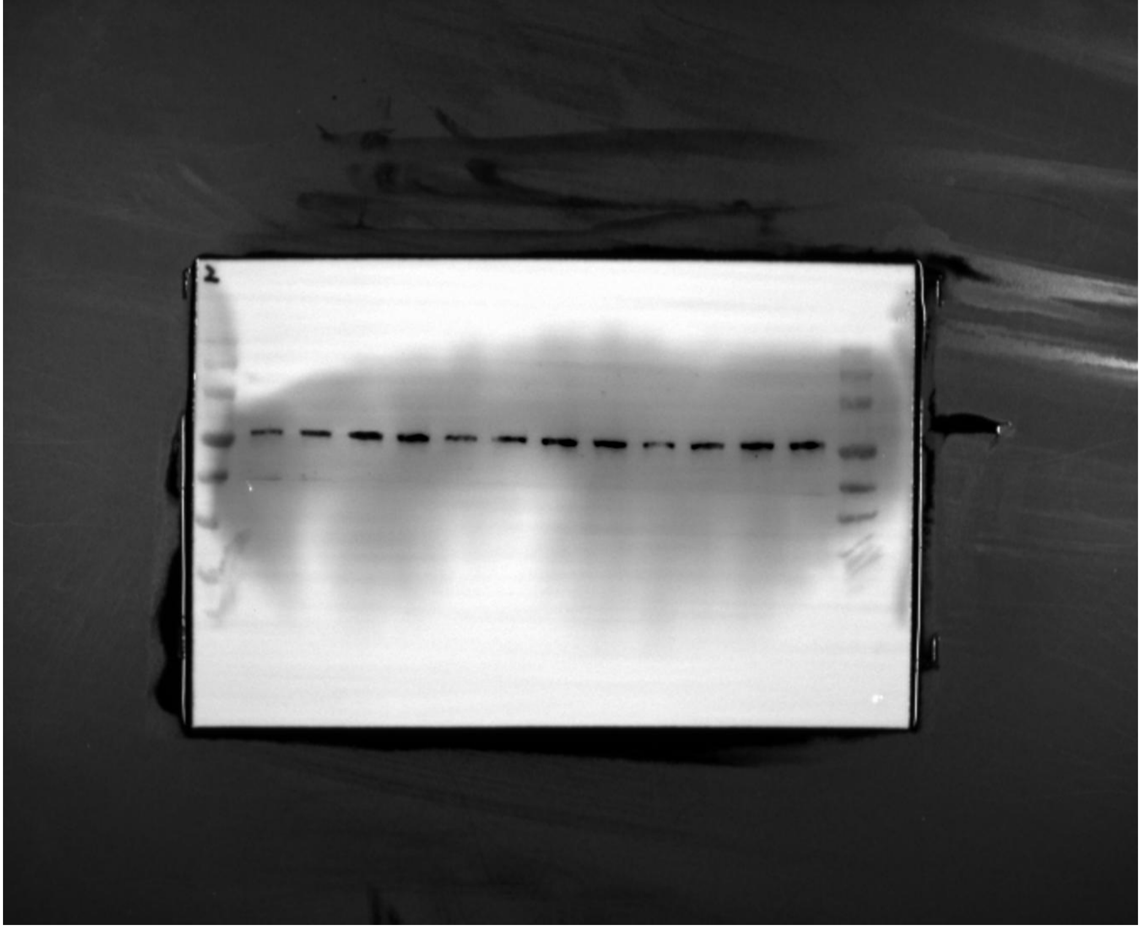
Figure 6D-O-GlcNAc-YAP1

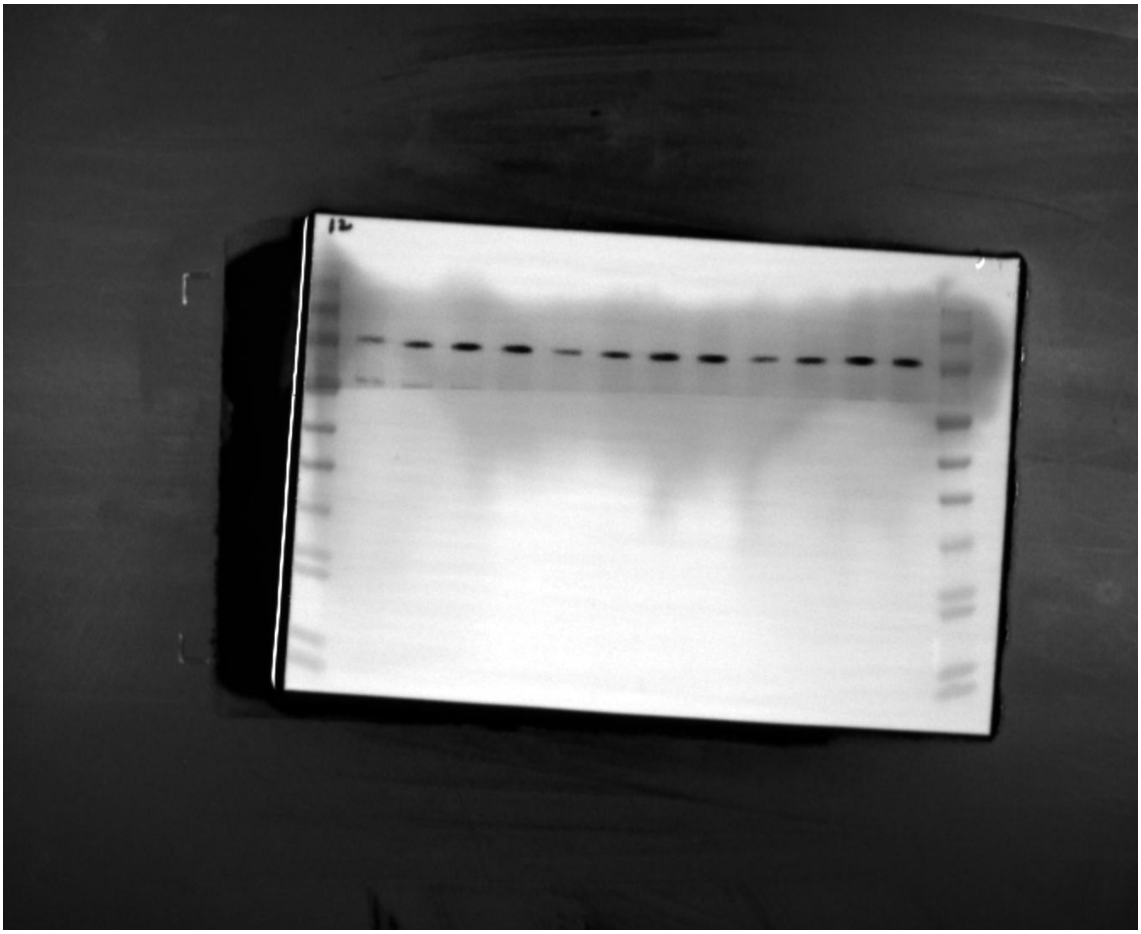
Figure 6D-HIF1A

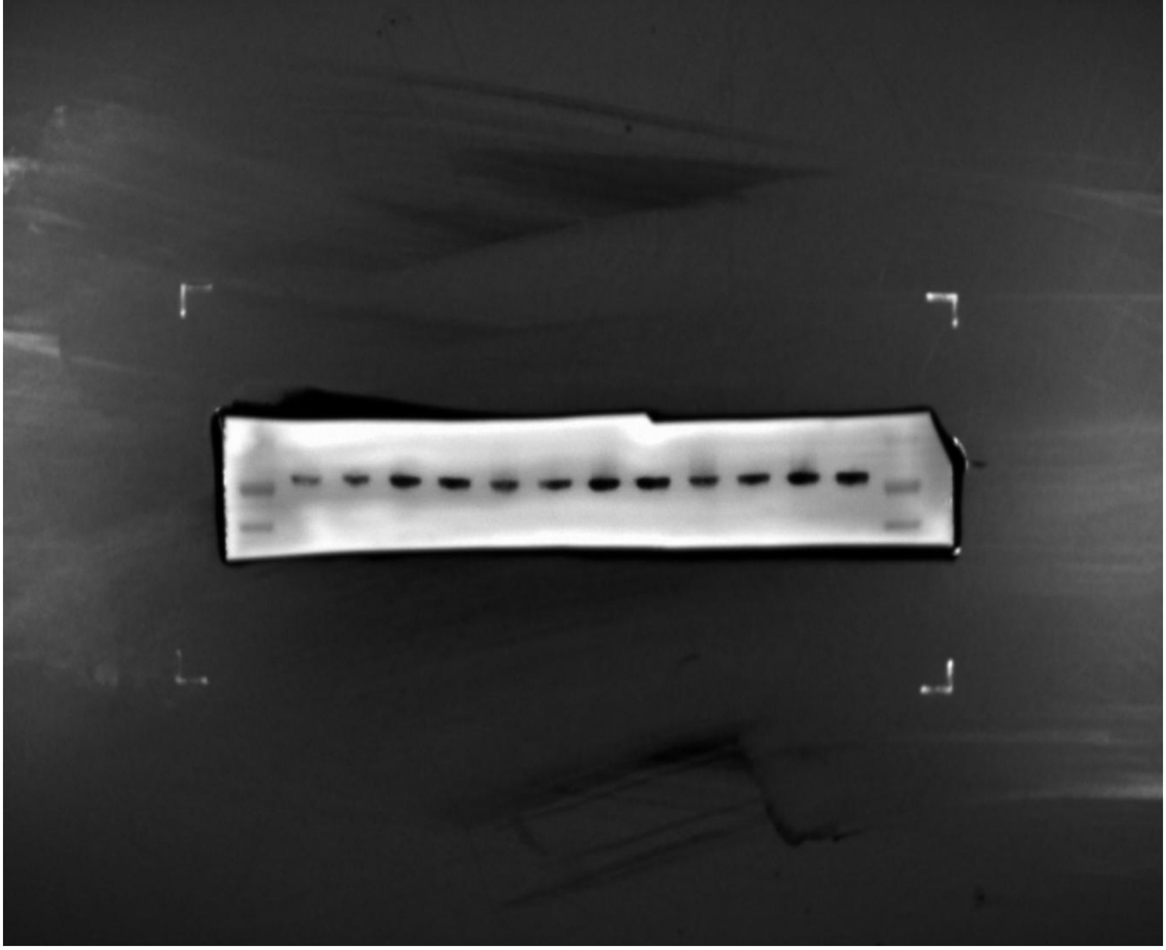

Figure 6D-YAP1

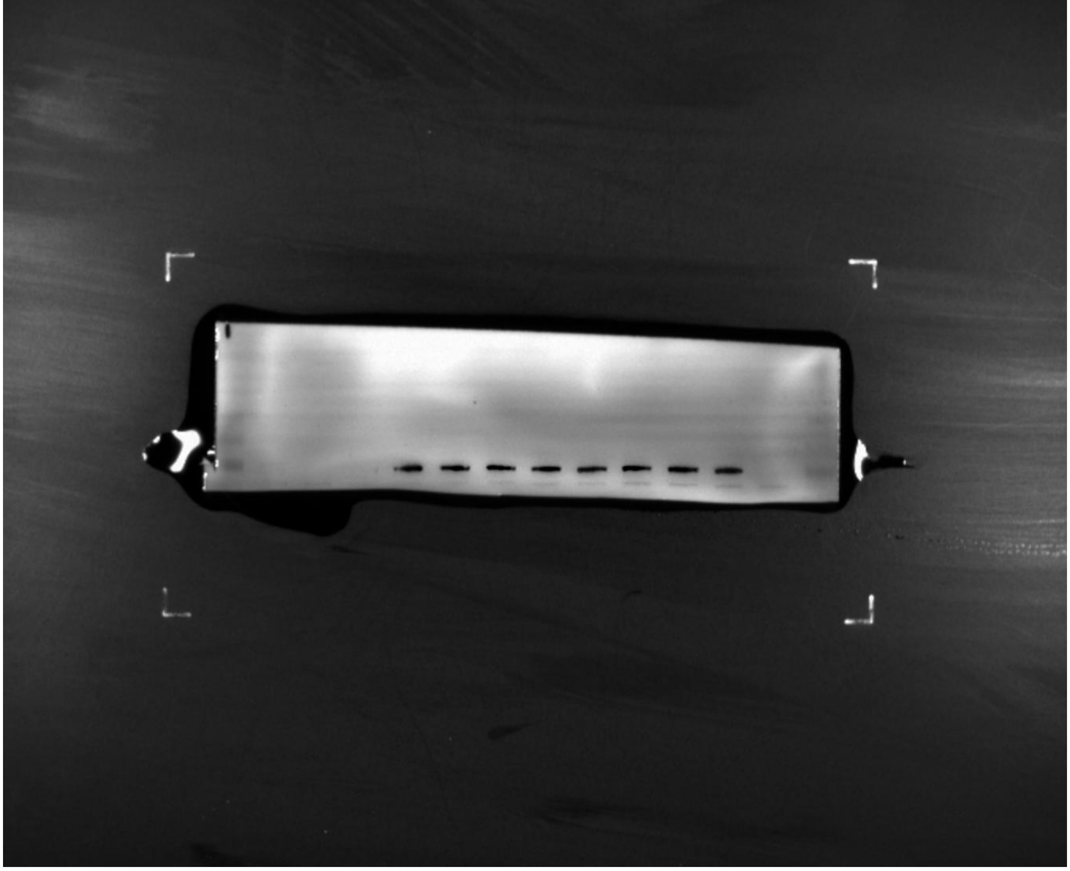

Figure 6E-Input-FLAG

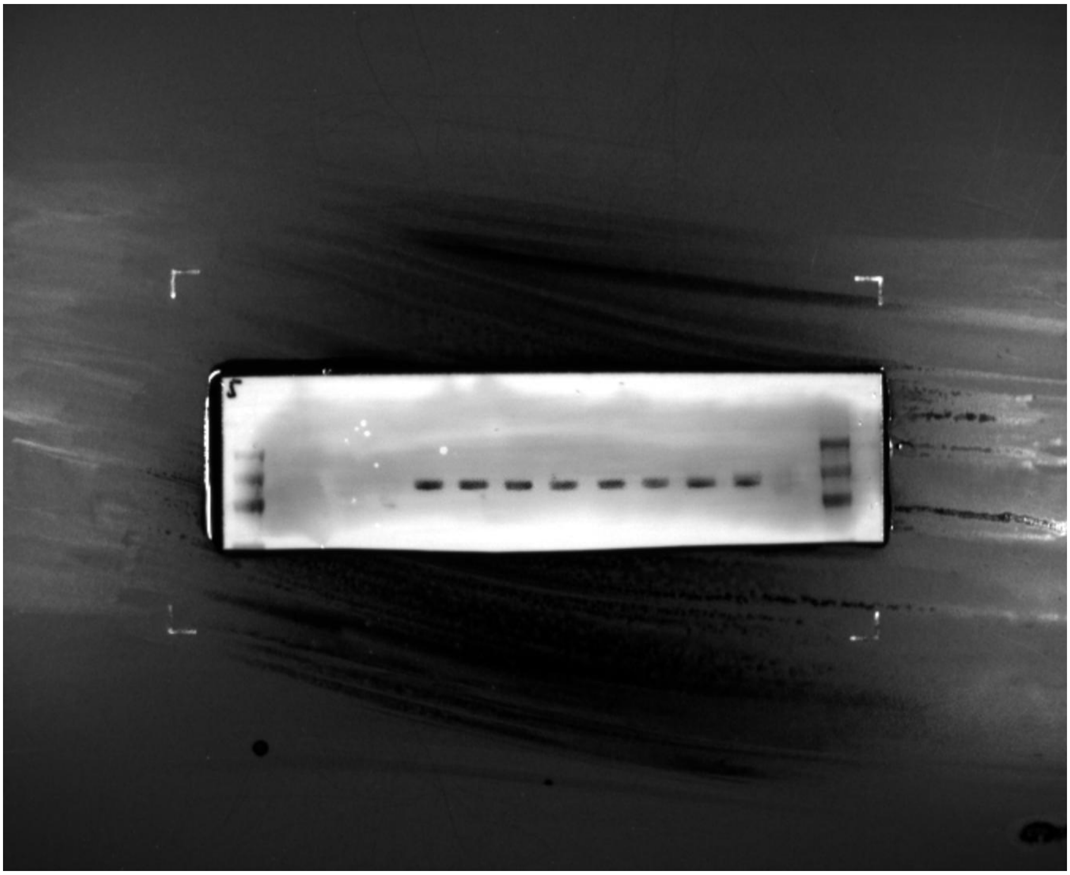

Figure 6E-IP-FLAG

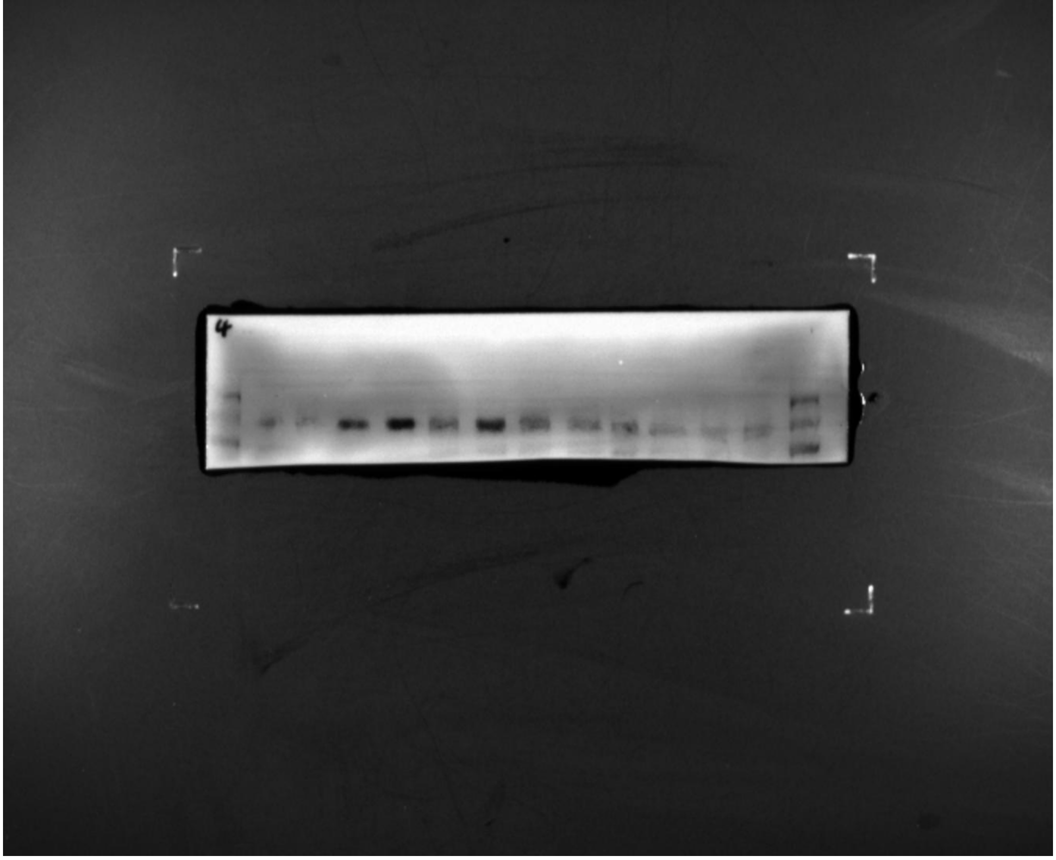

Figure 6E-O-GlcNAc-YAP1

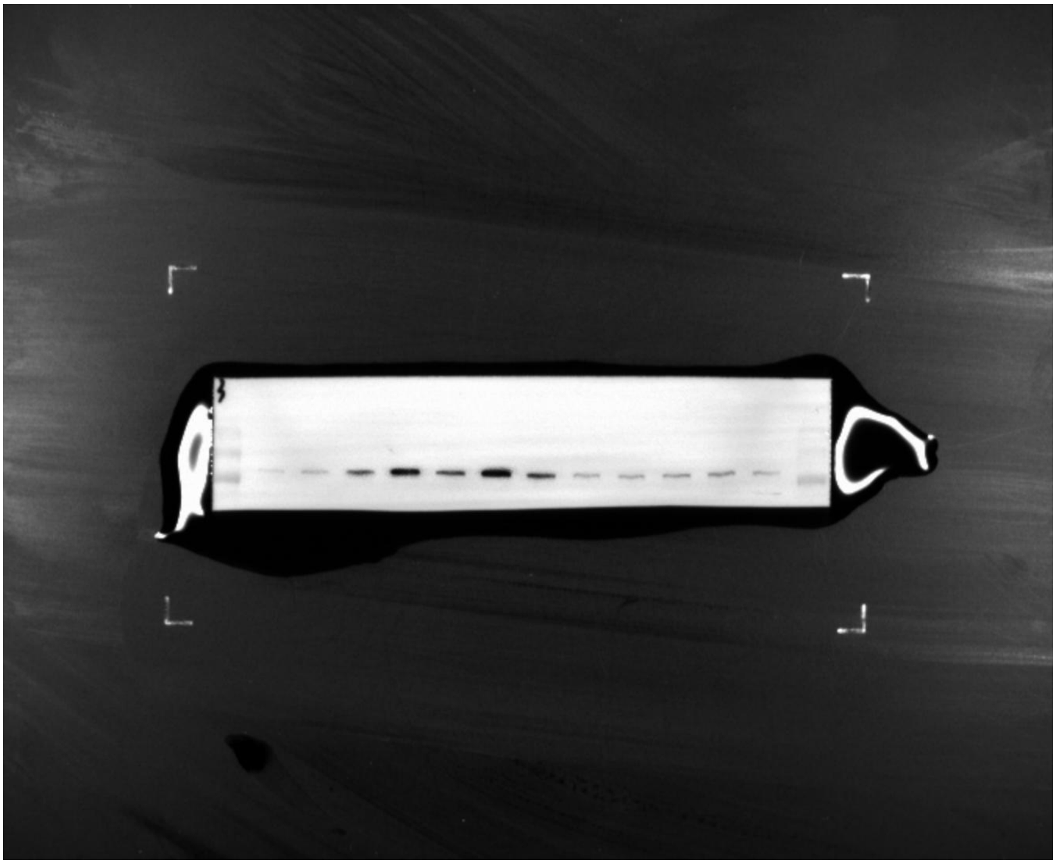

Figure 6E-HIF1A

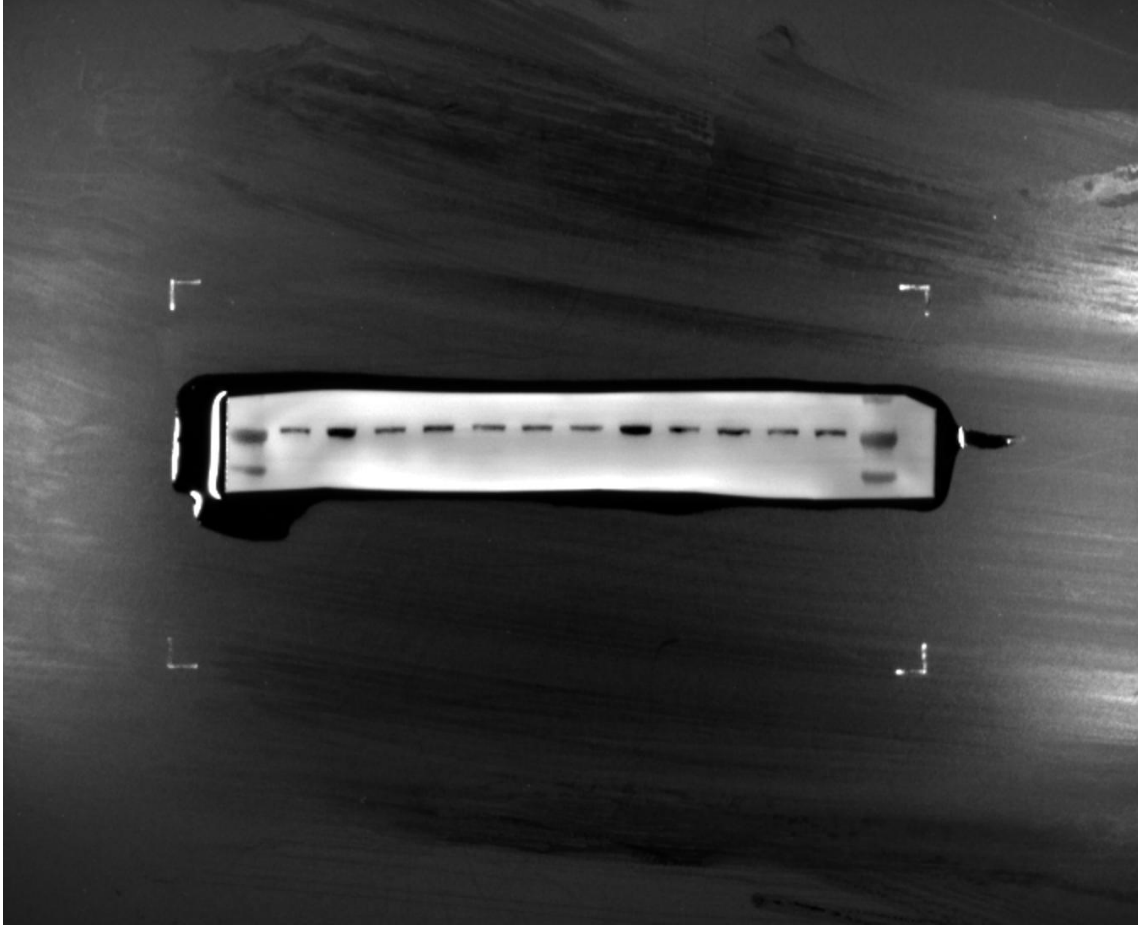

Figure 7B-YAP1

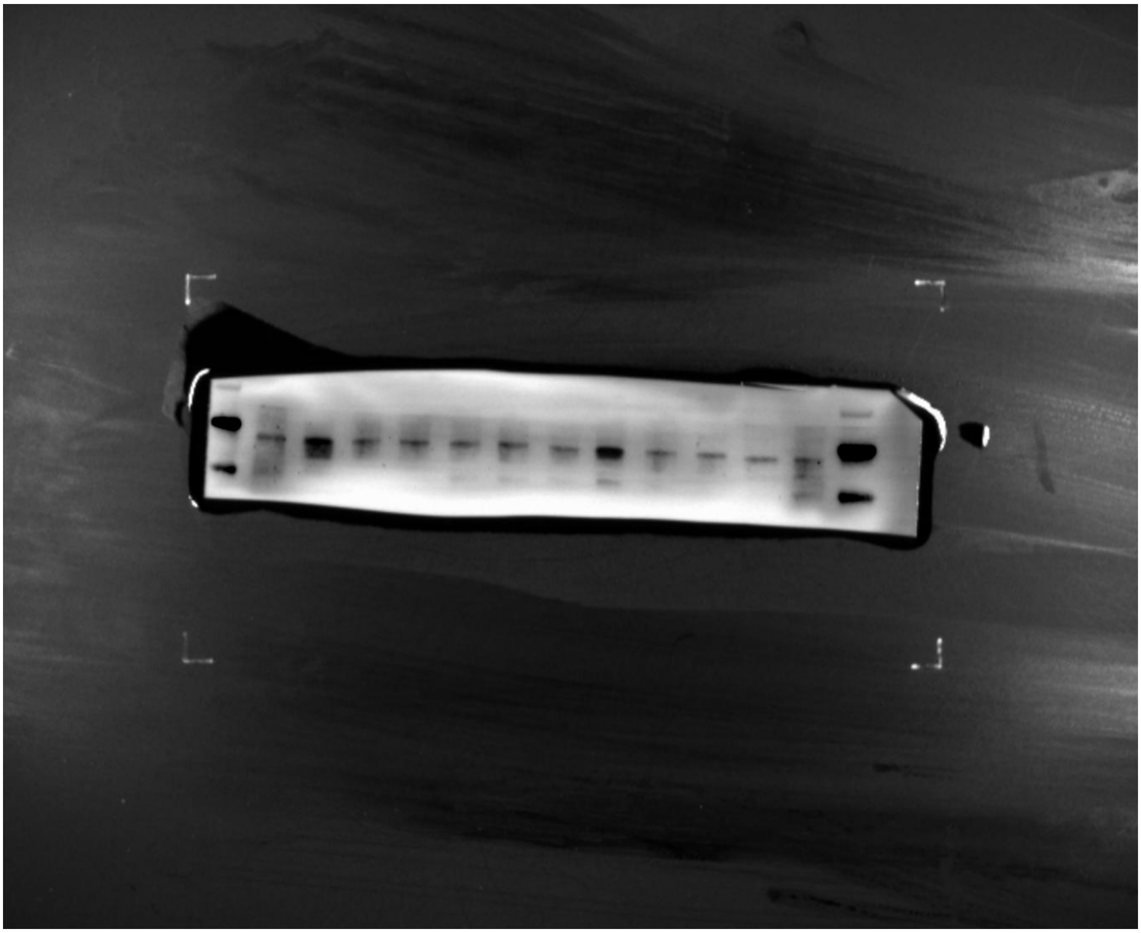

Figure 7B-CYR61

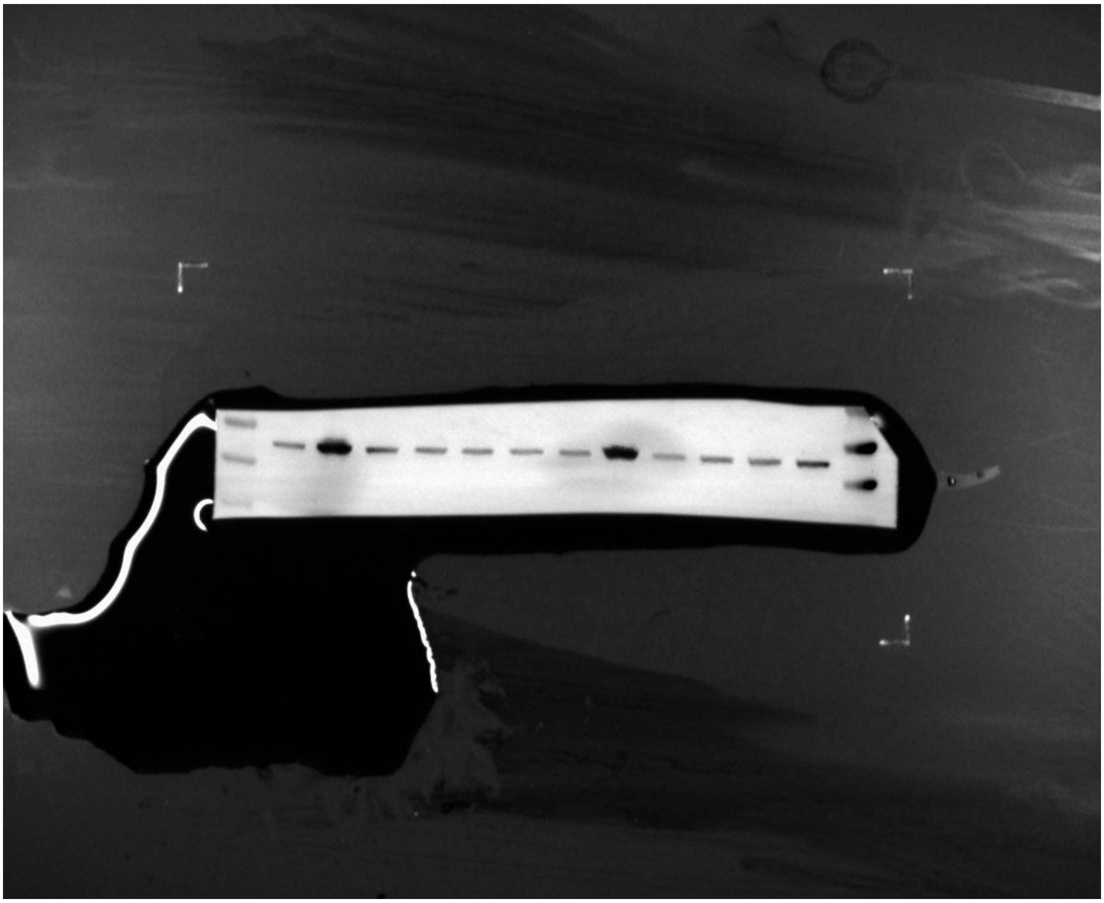

Figure 7B-AREG

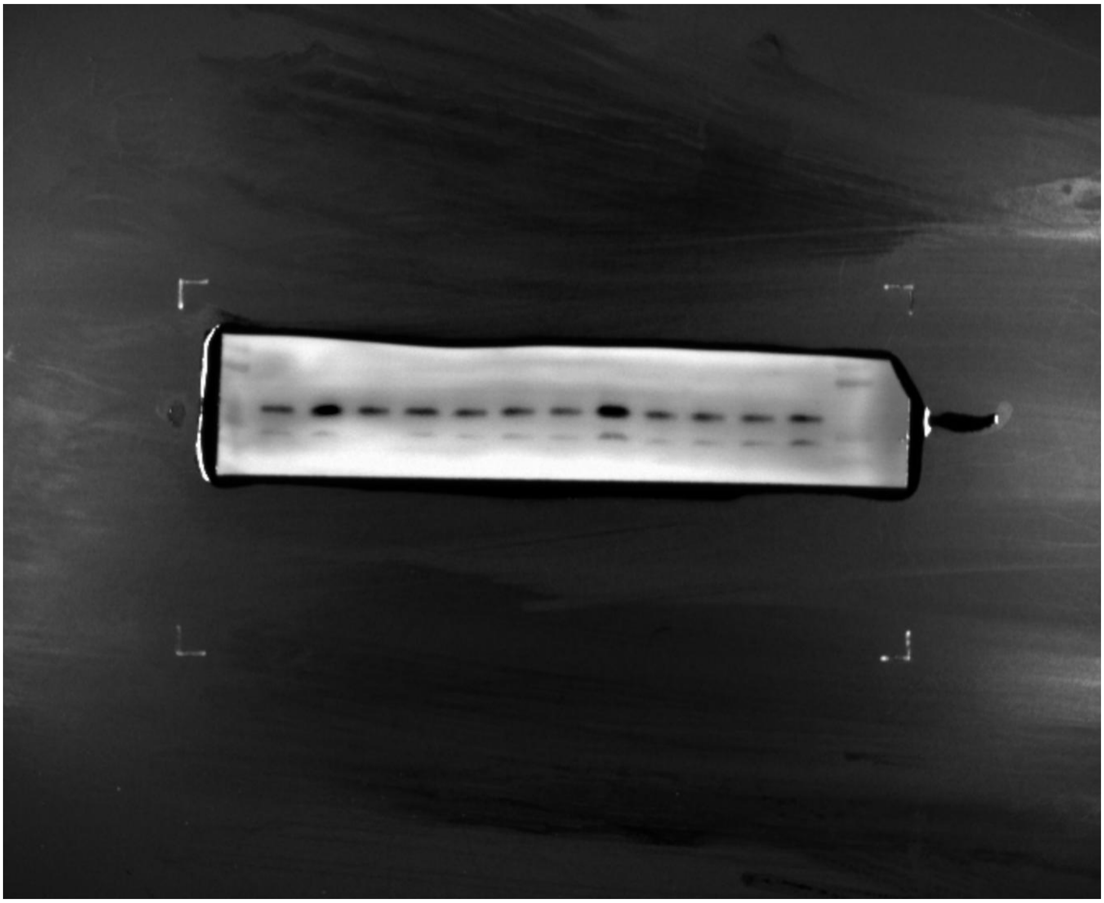

Figure 7B-BIRC5

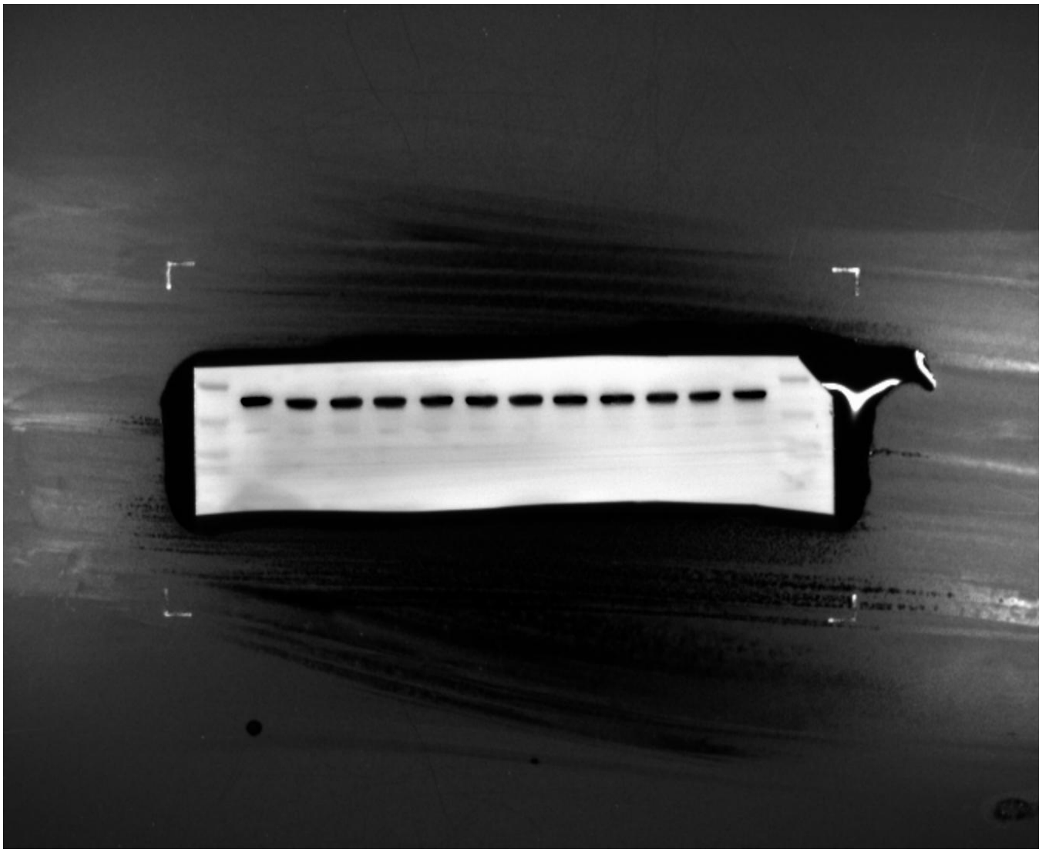

Figure 7B-GAPDH

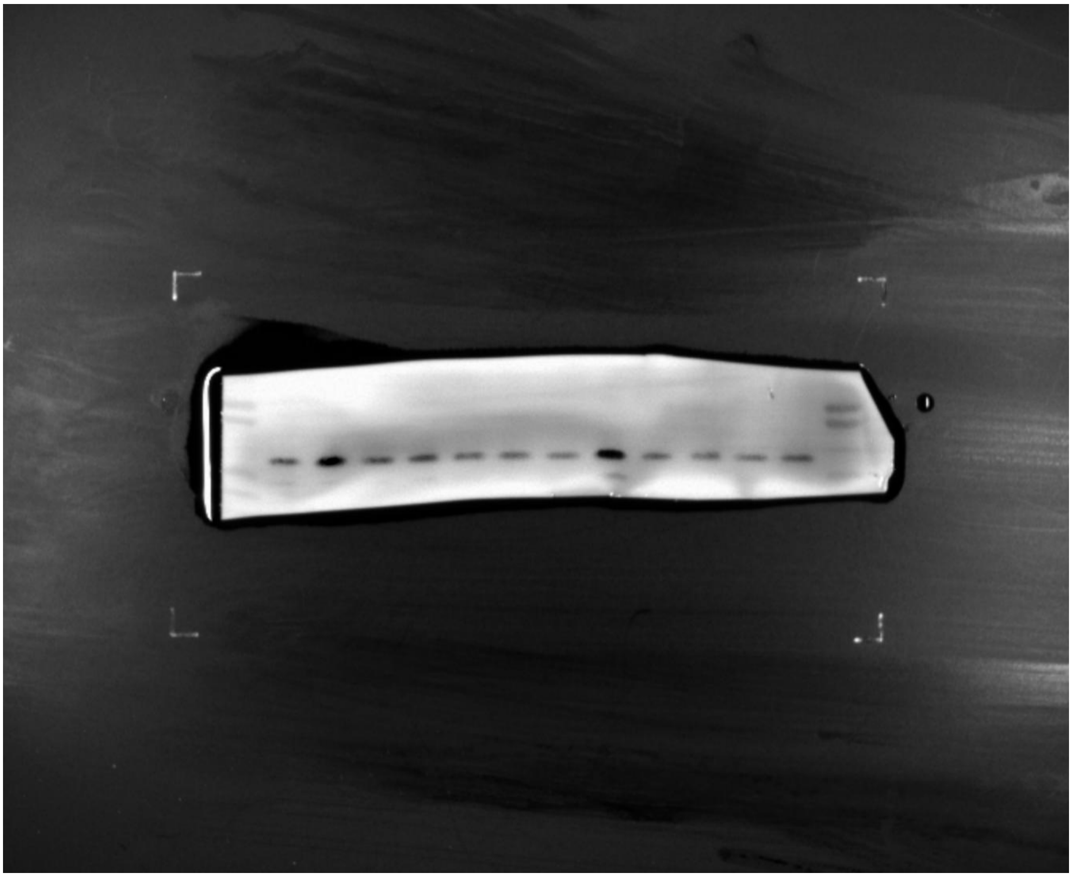

Figure 7E-FUND1

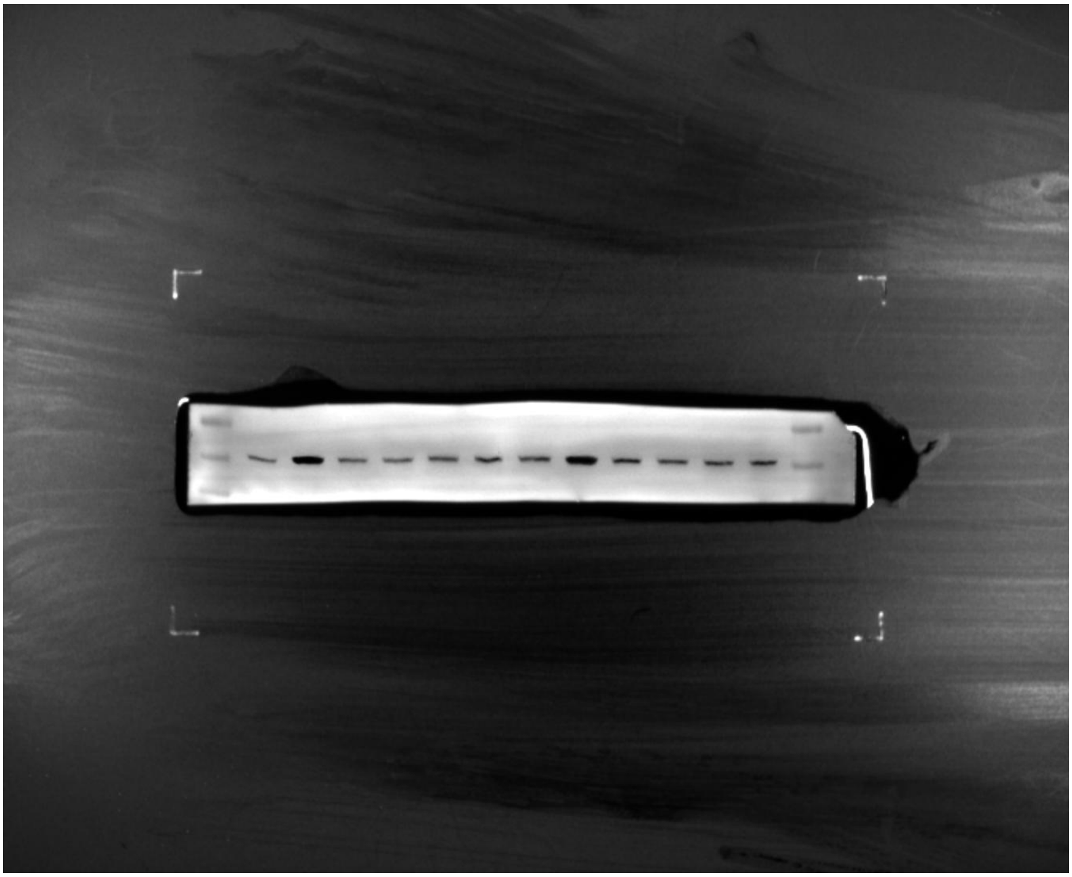

Figure 7E-PINK1

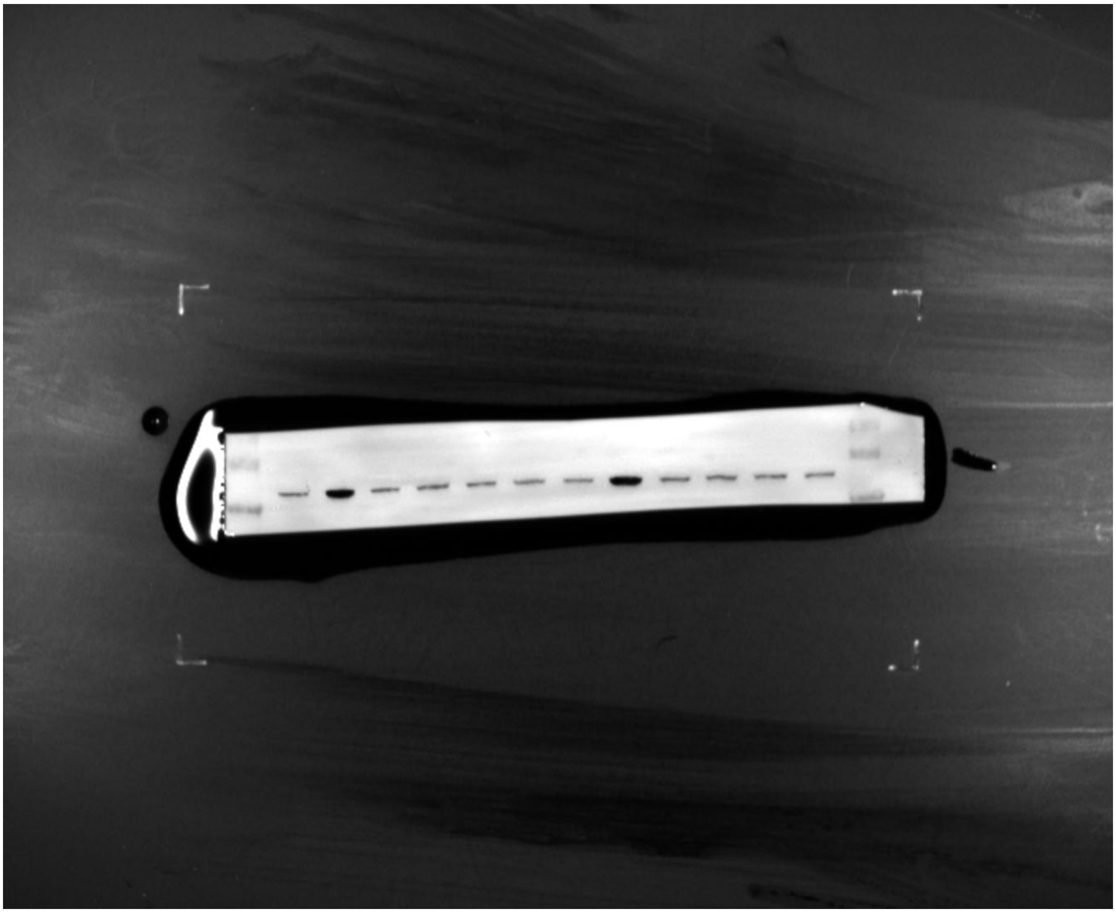

Figure 7E-TBK1

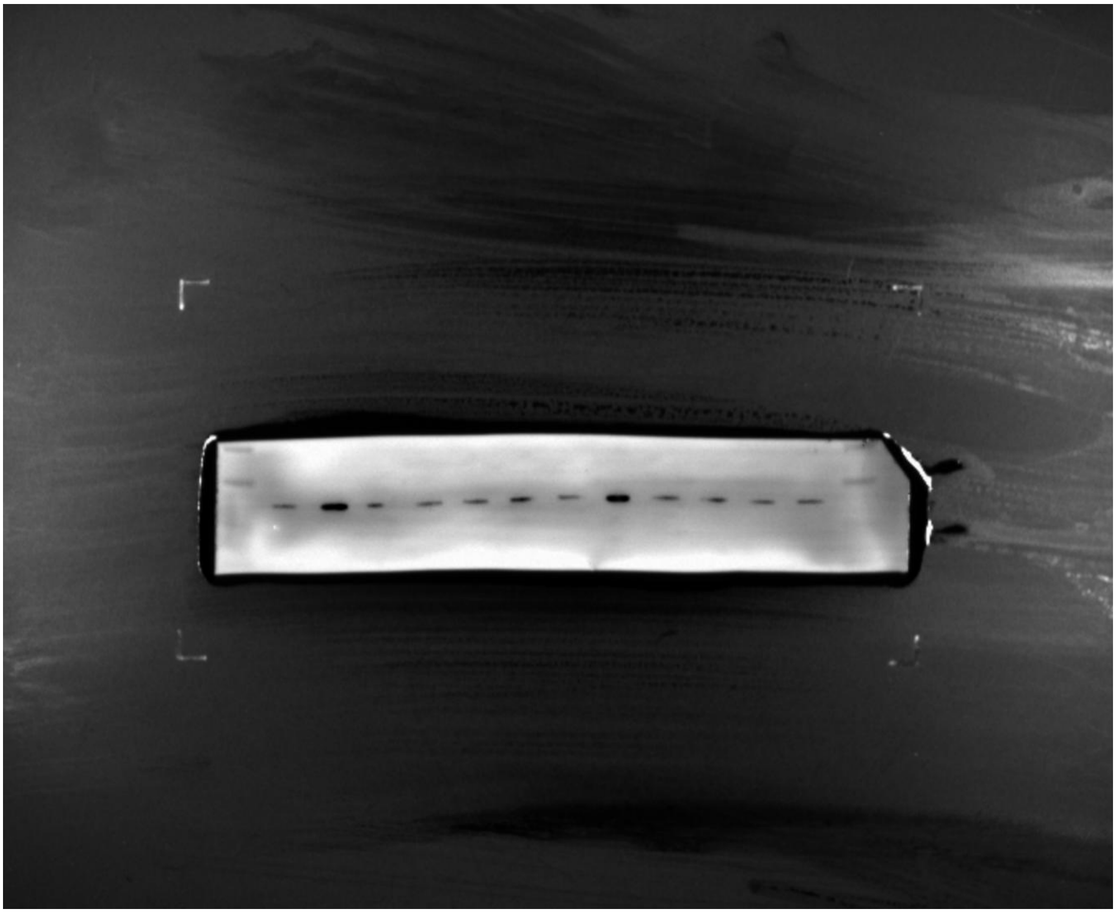

Figure 7E-HMGB1

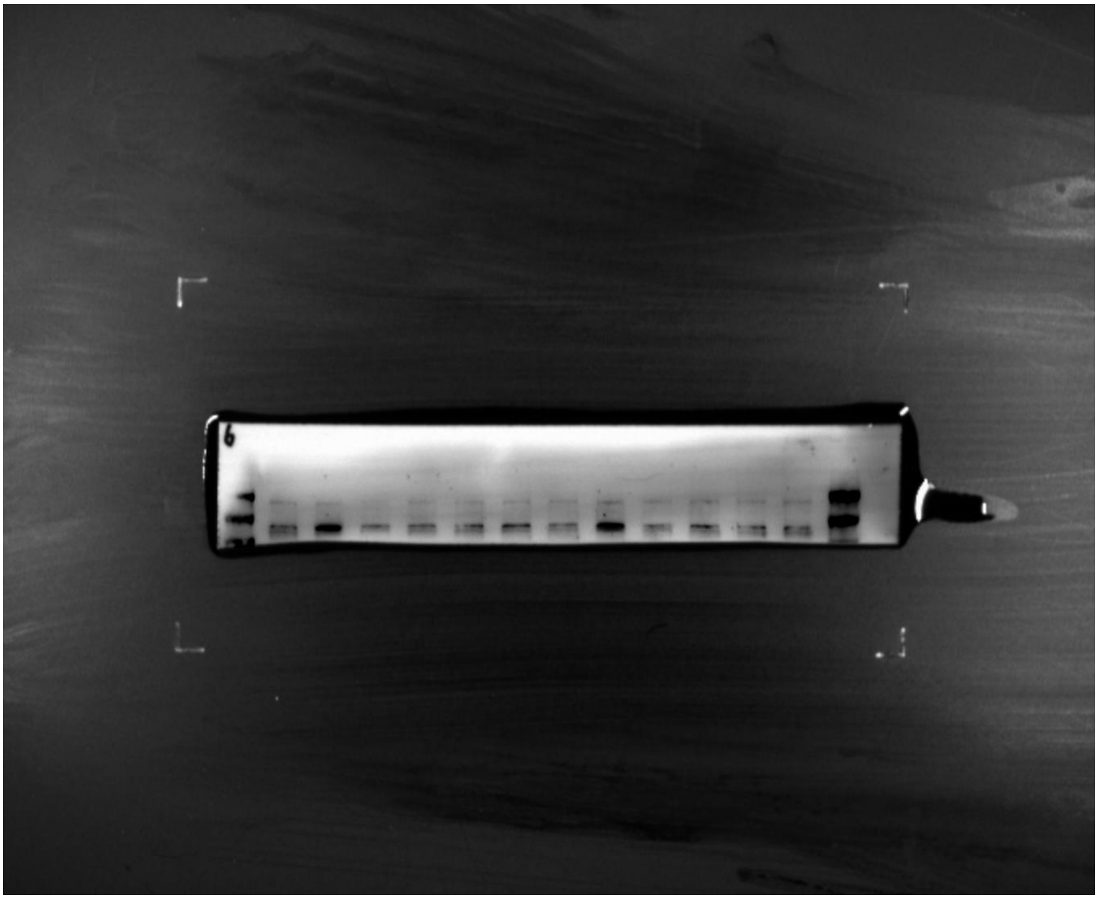

Figure 7E-DAPK

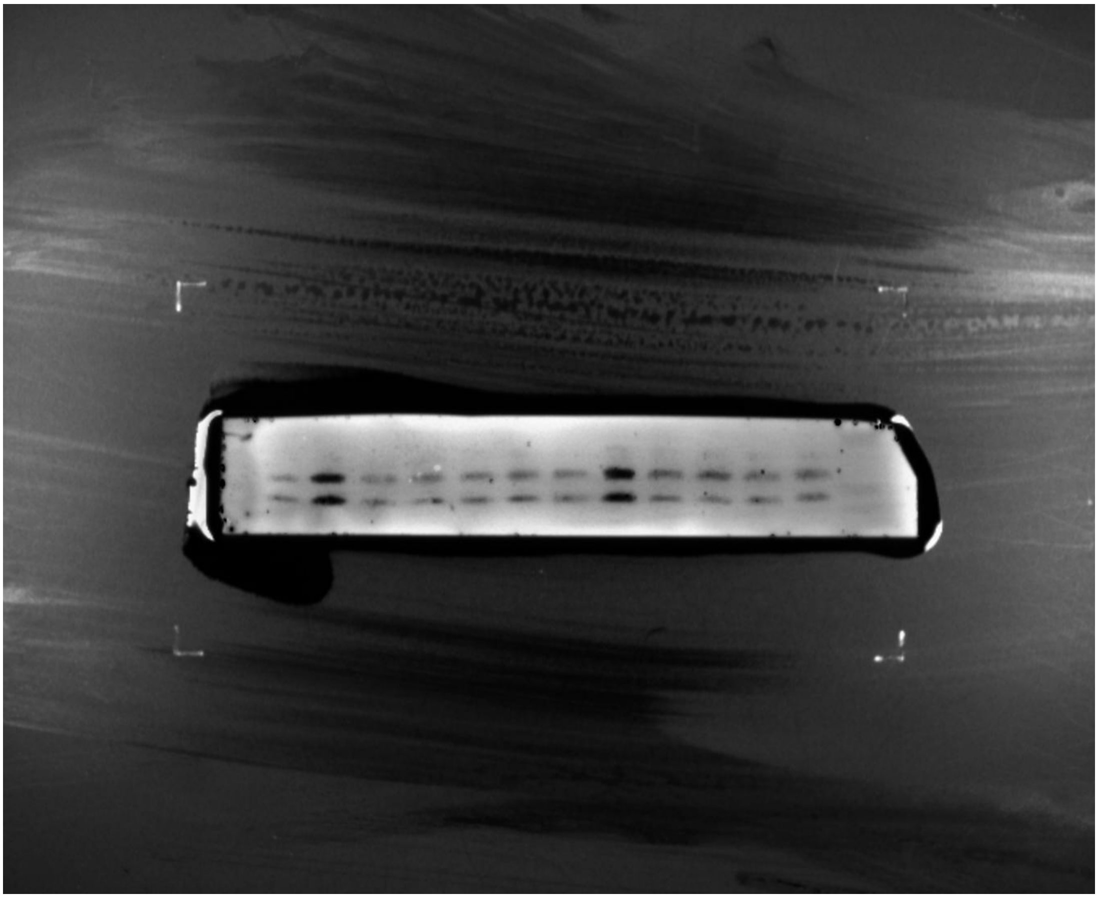

Figure 7E-LC3-I/II

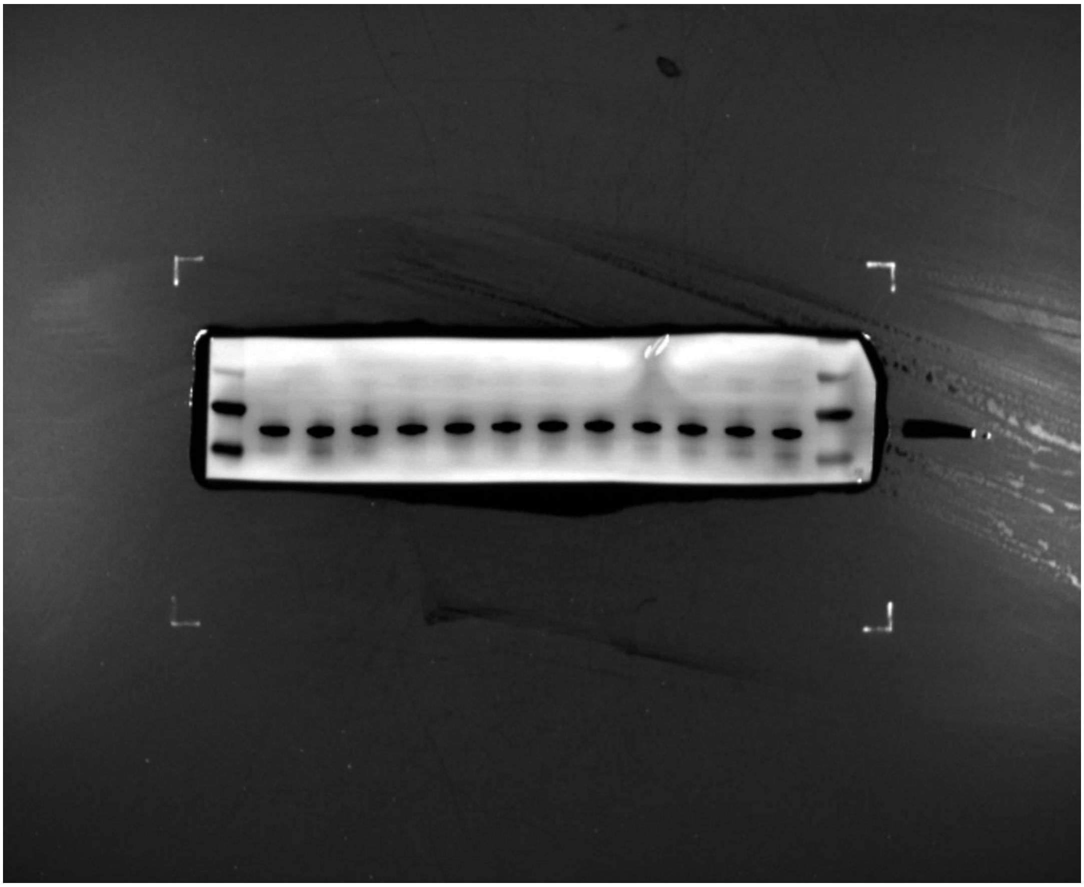

Figure 7E-GAPDH

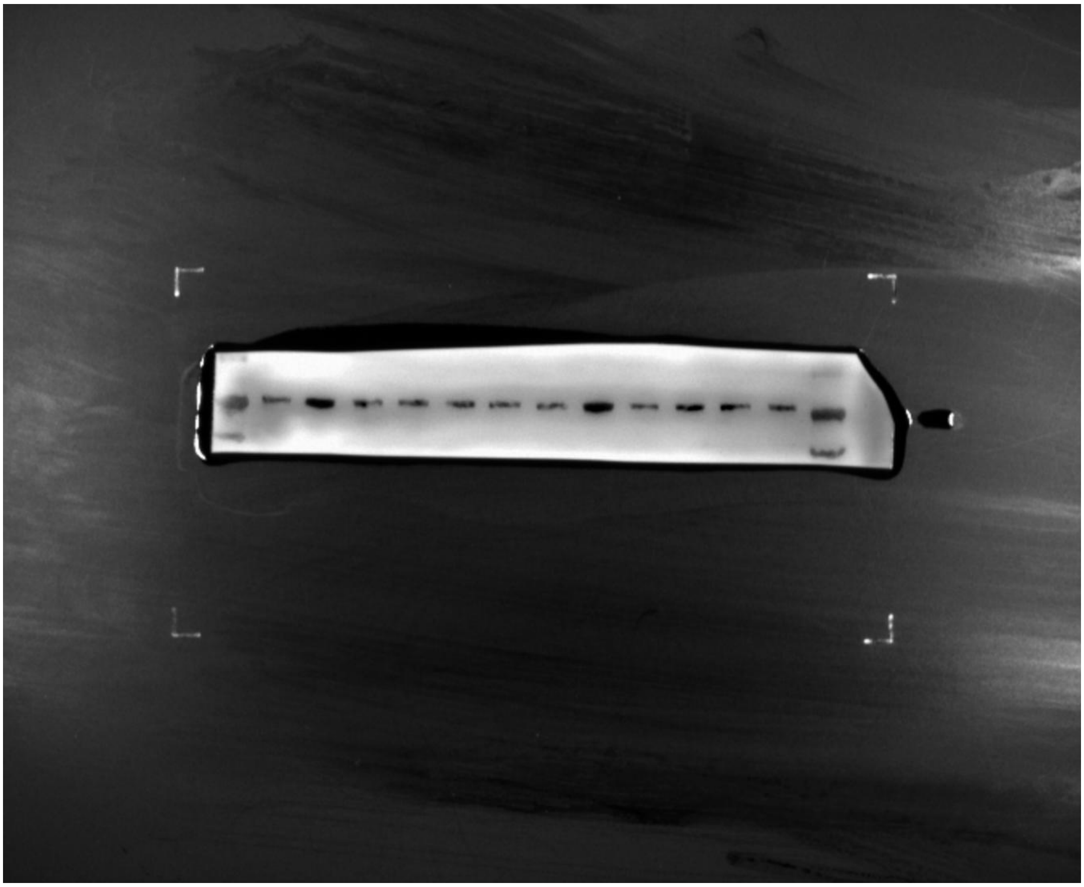

Figure 8C-YAP1

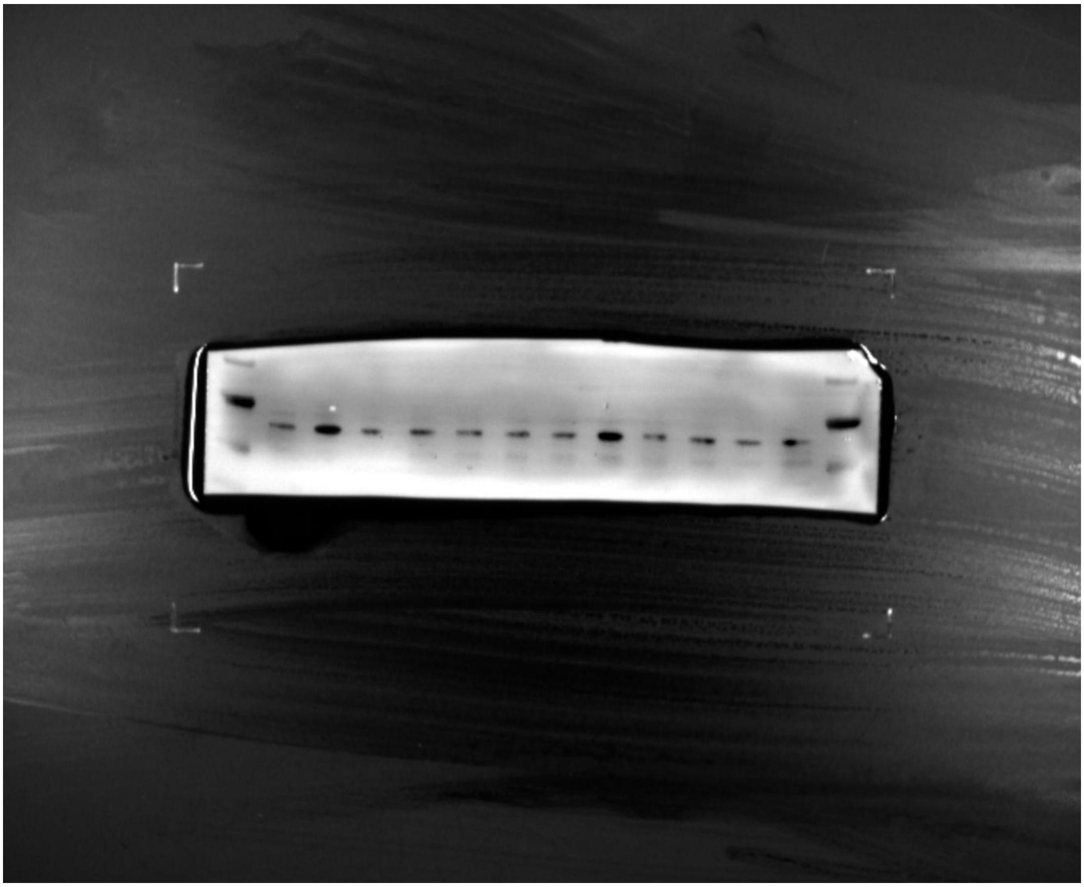

Figure 8C-CTGF

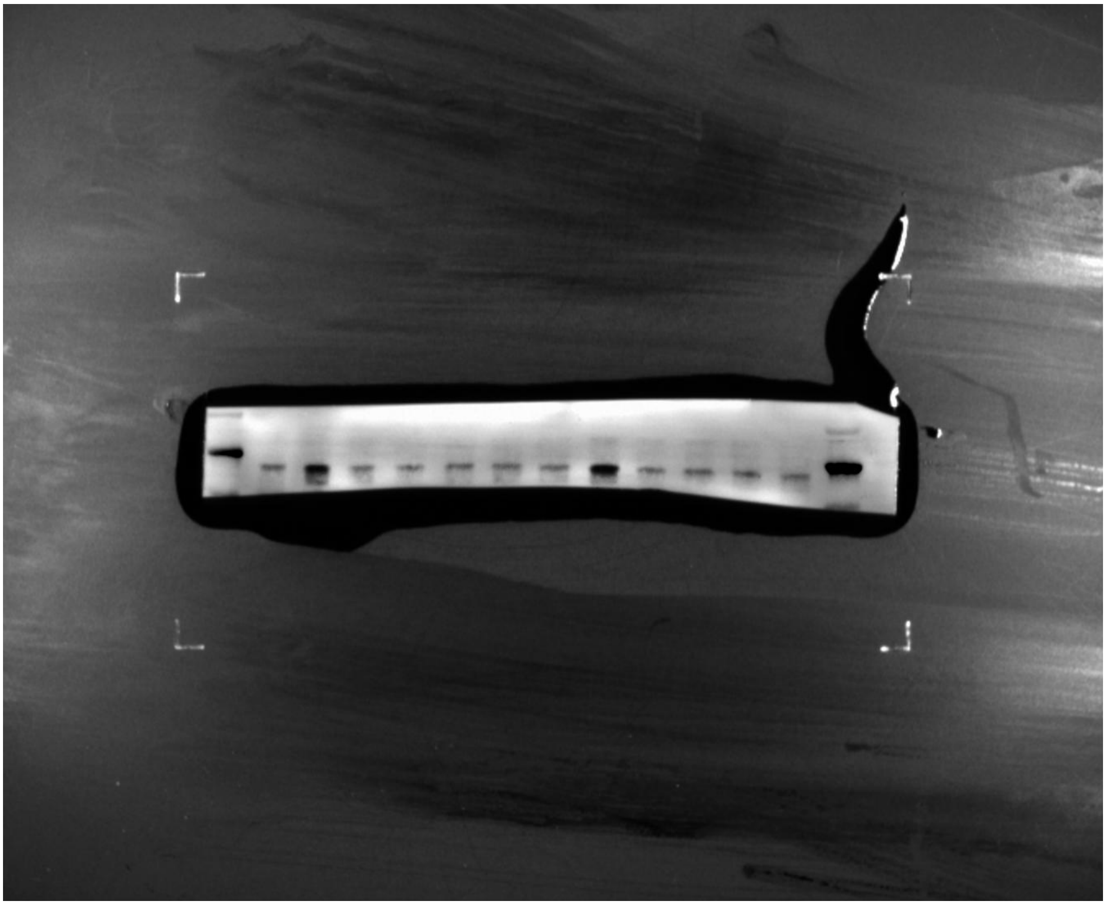

Figure 8C-CYR61

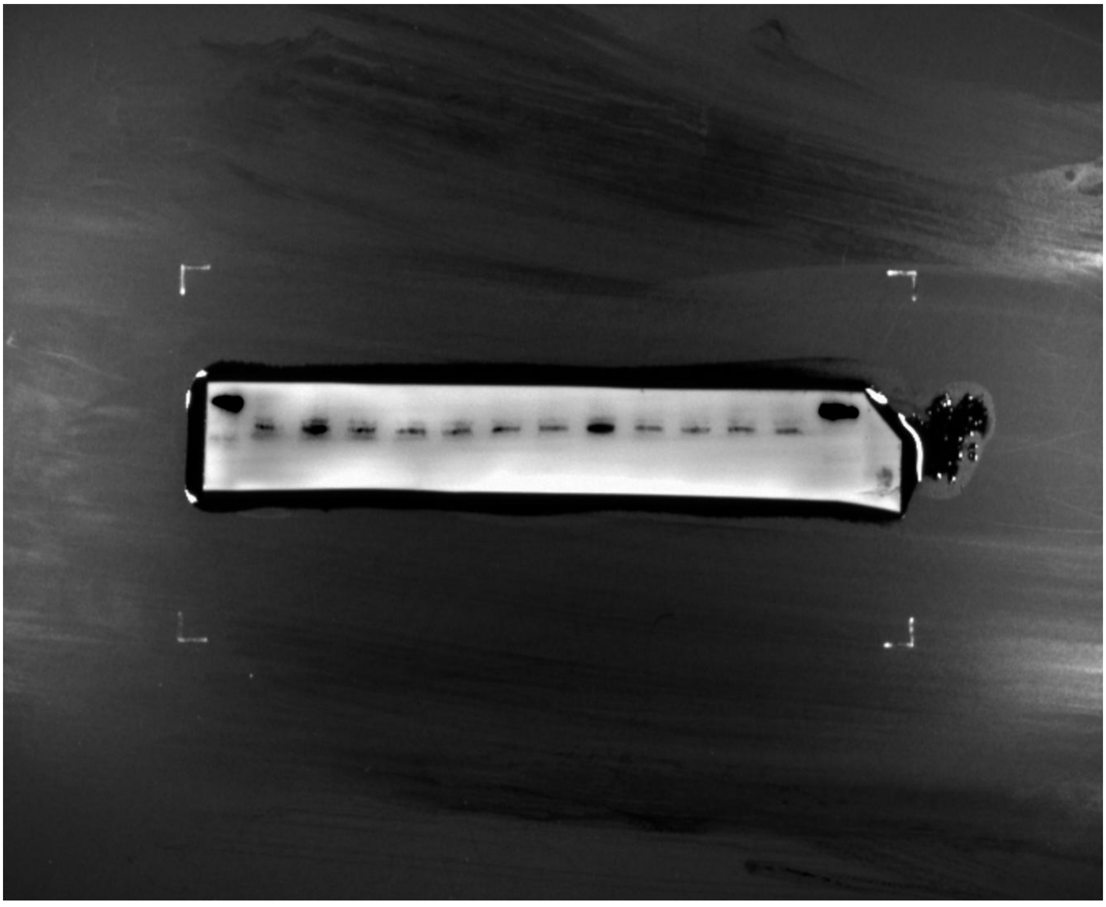

Figure 8C-AREG

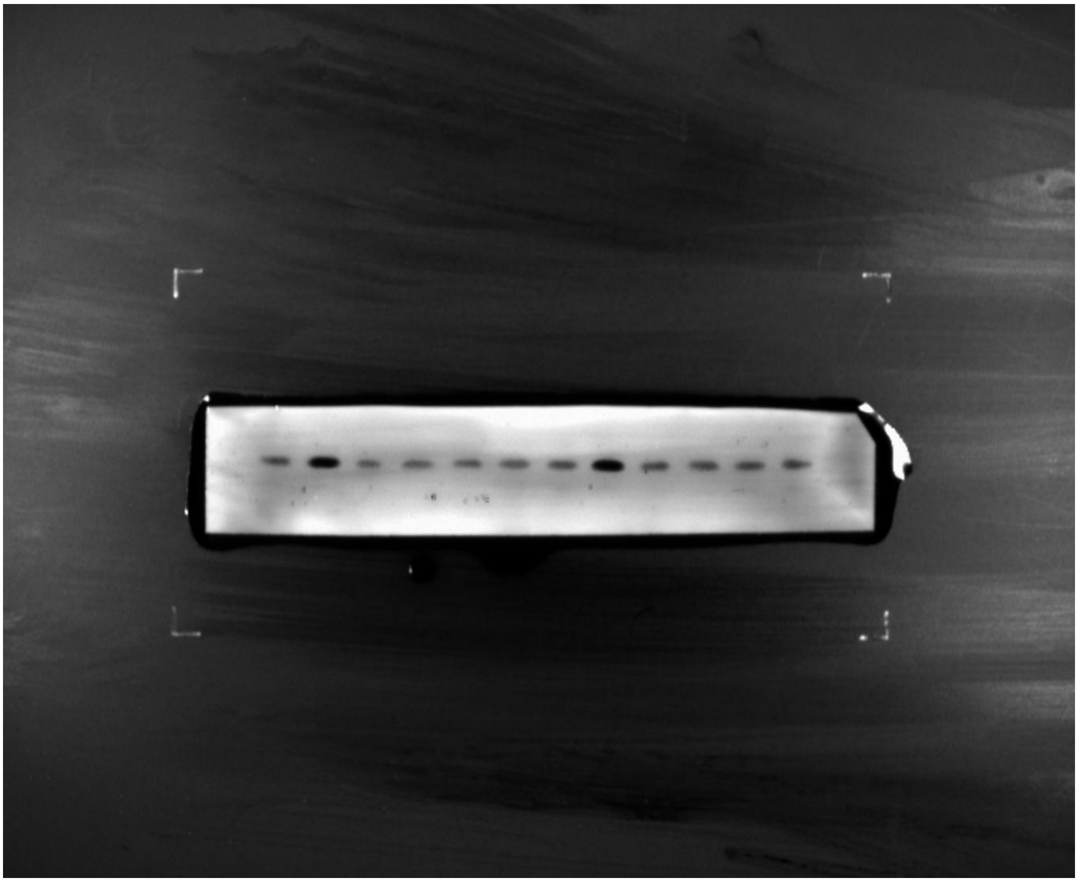

Figure 8C-BIRC5

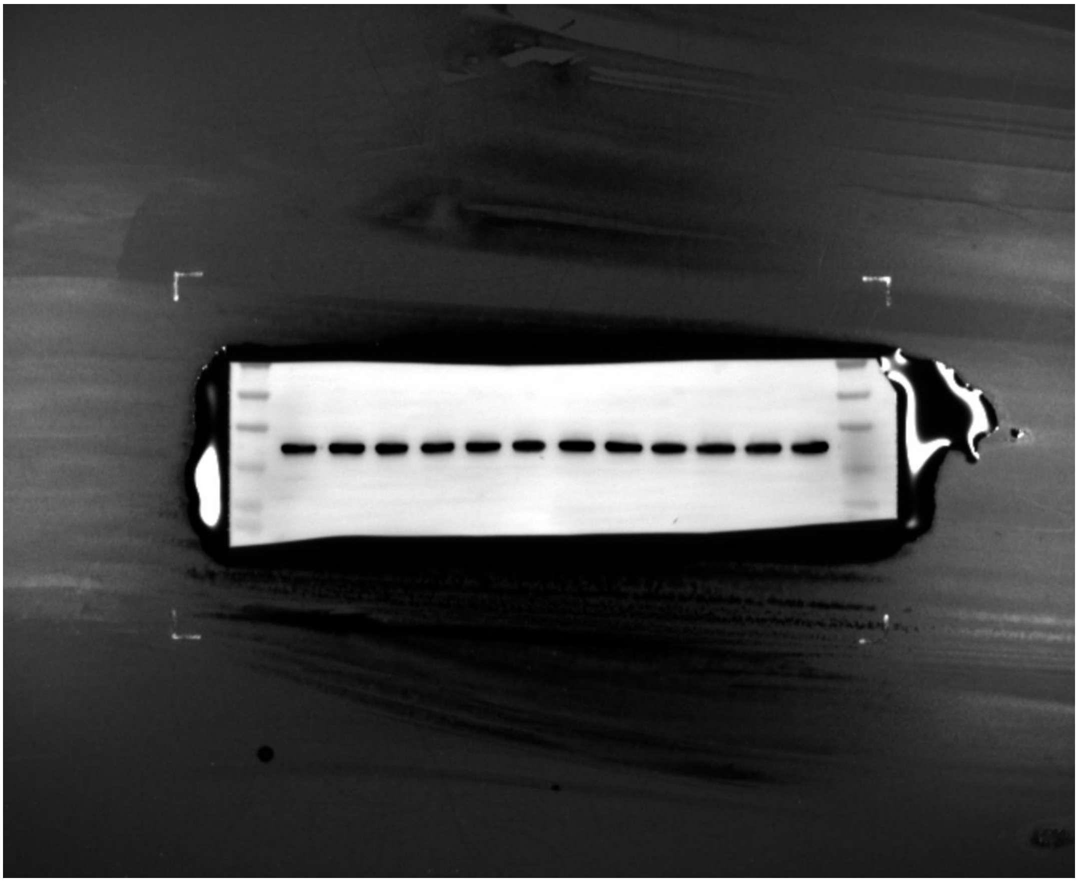

Figure 8C-GAPDH

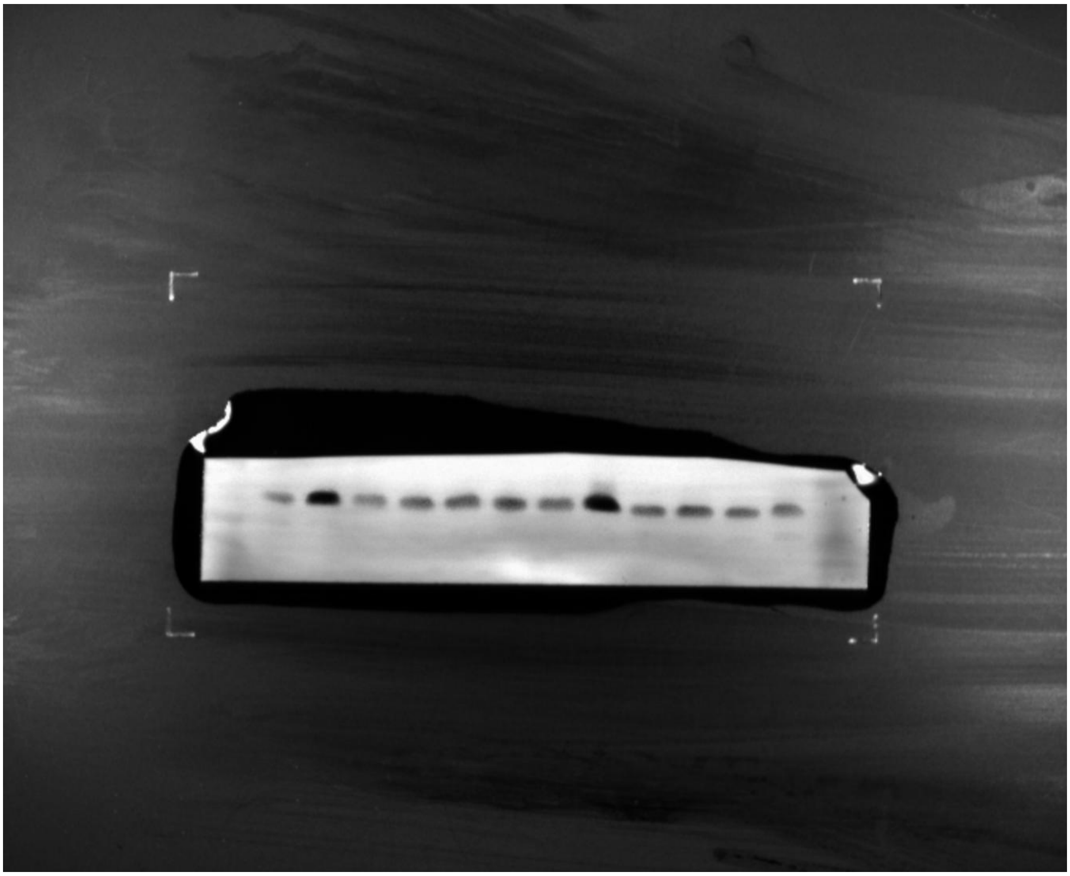

Figure 8E-FUND1

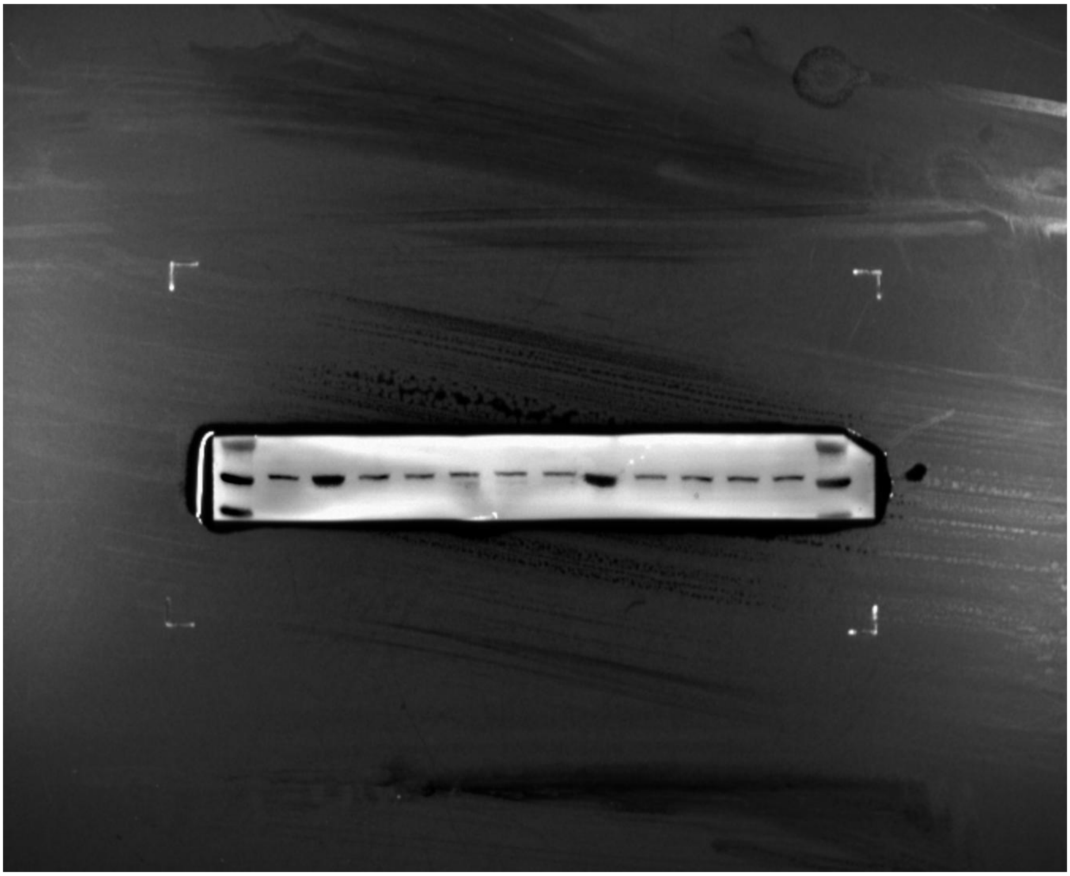

Figure 8E-PINK1

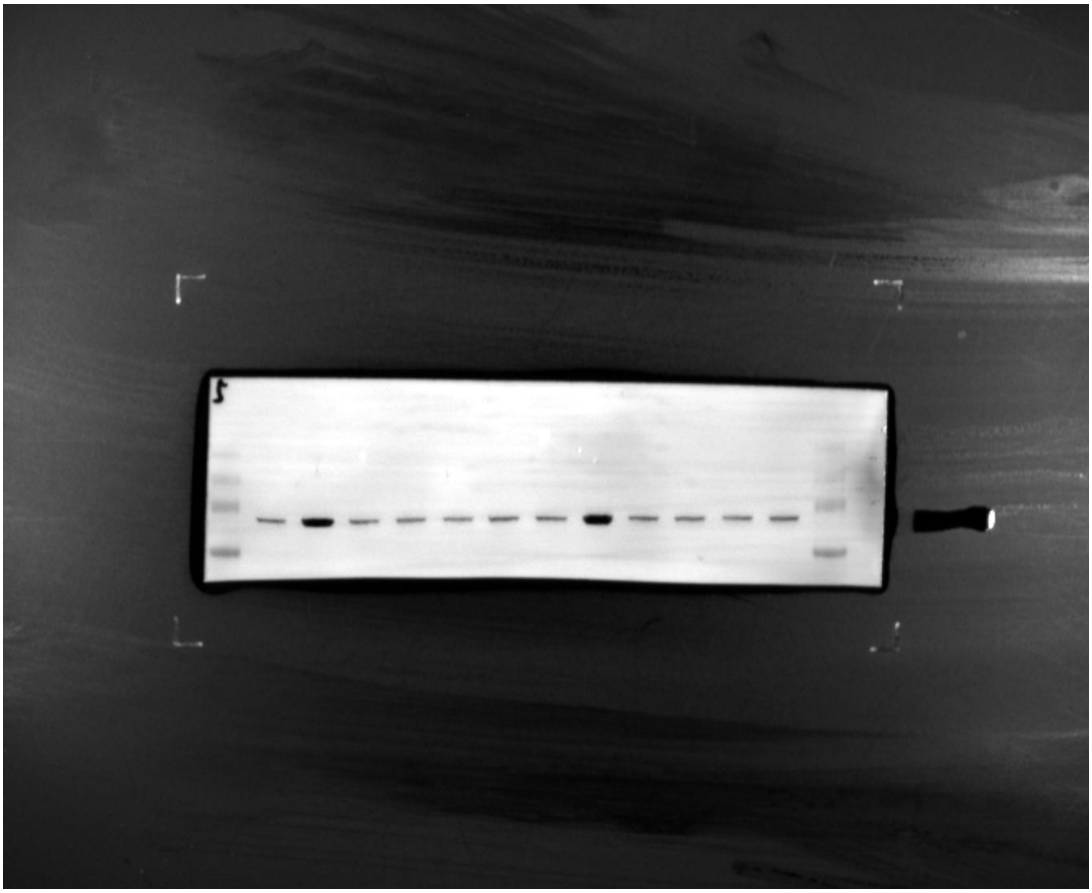

Figure 8E-TBK1

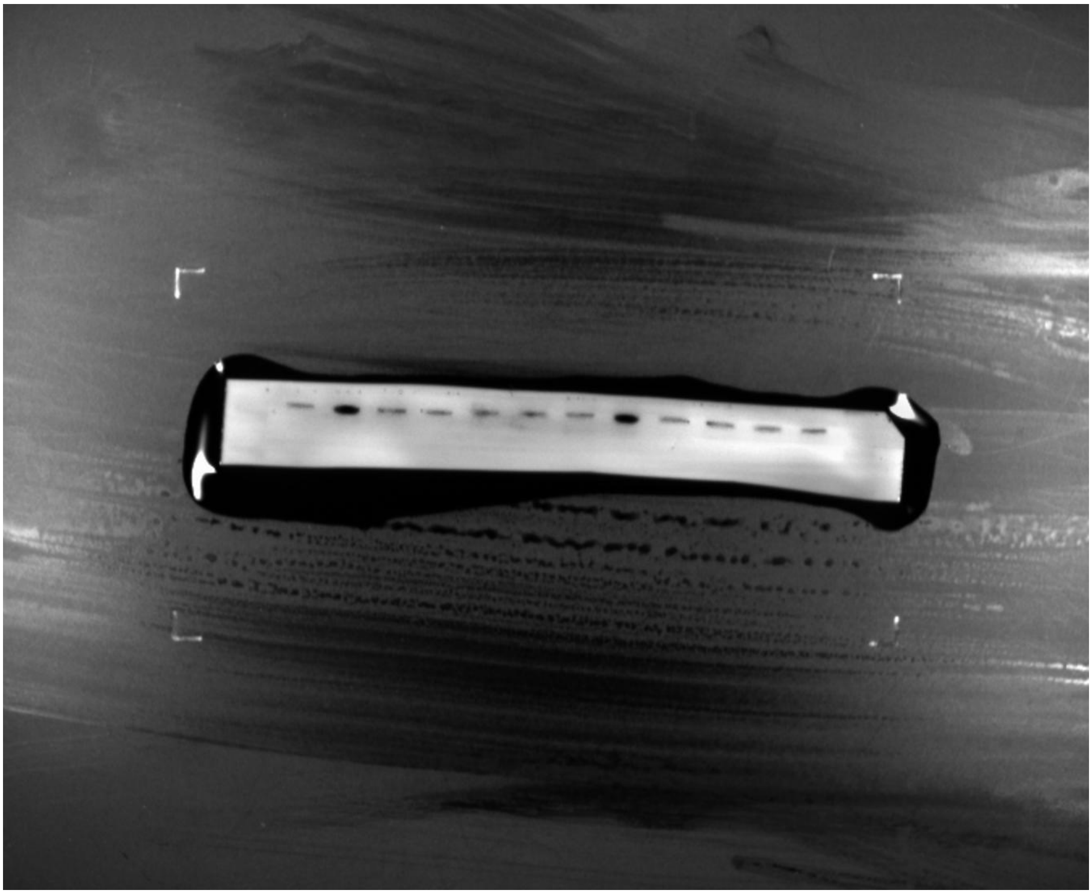

Figure 8E-HMGB1

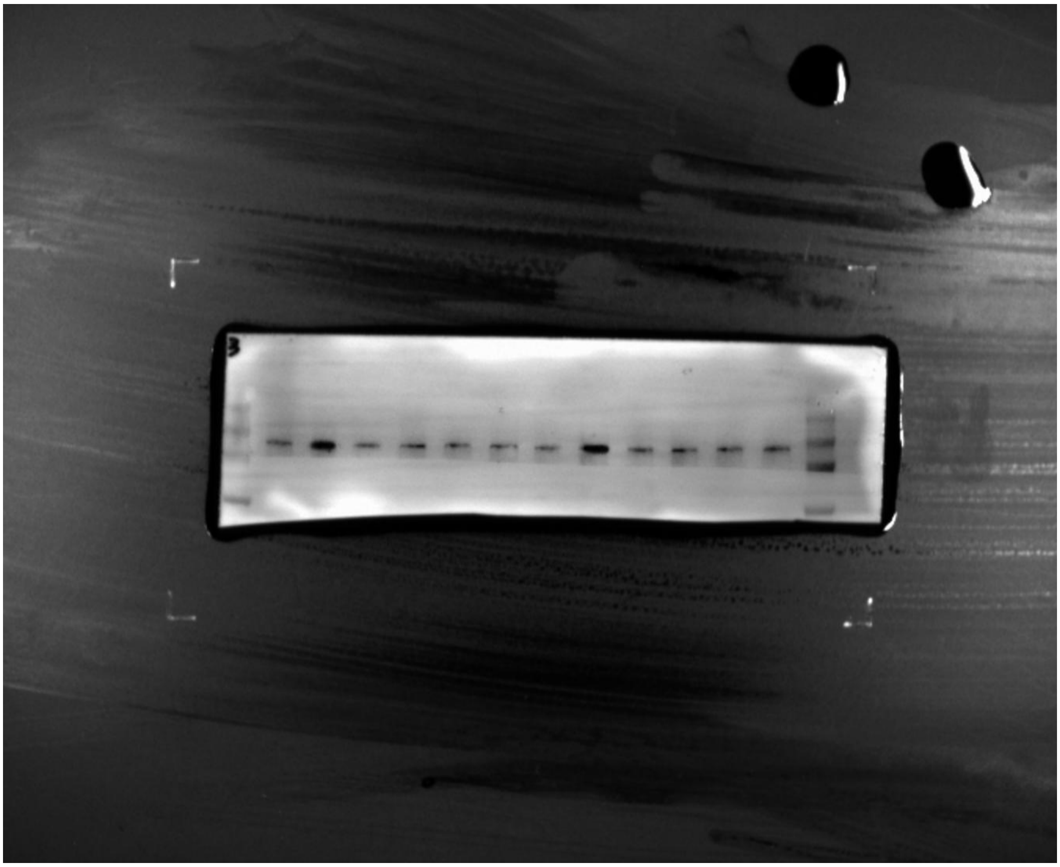

Figure 8E-DAPK

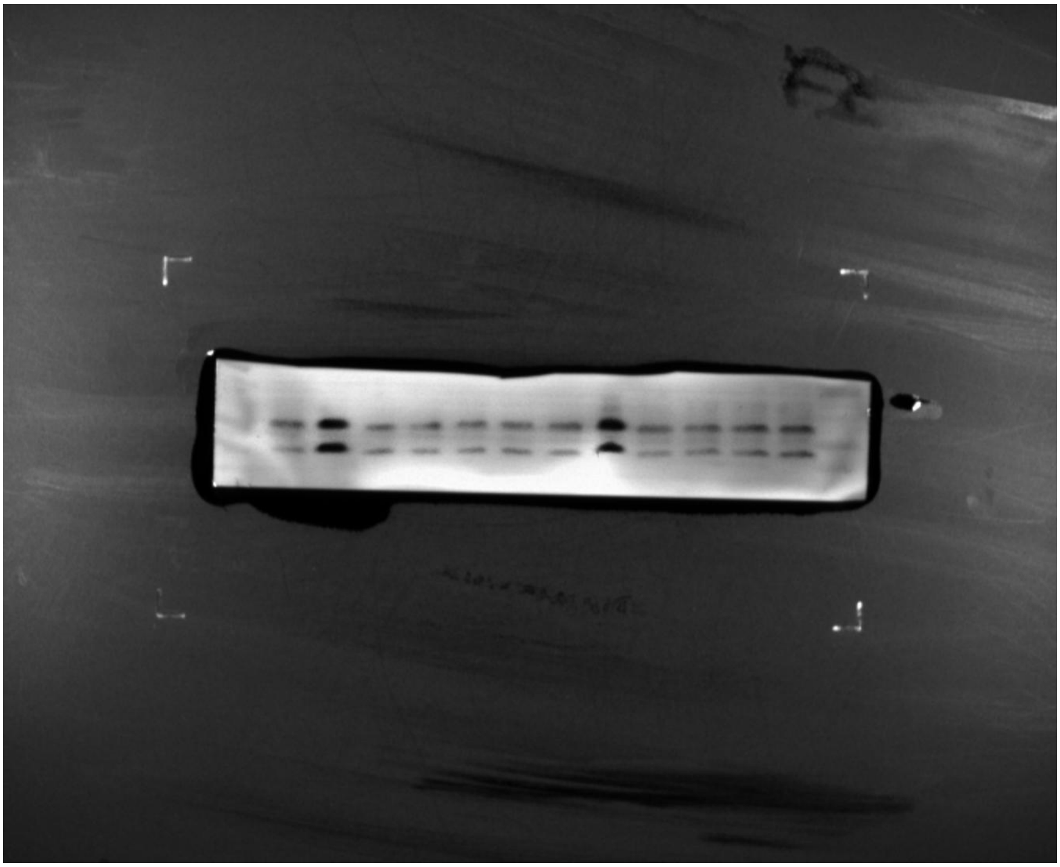

Figure 8E-LC3-I/II

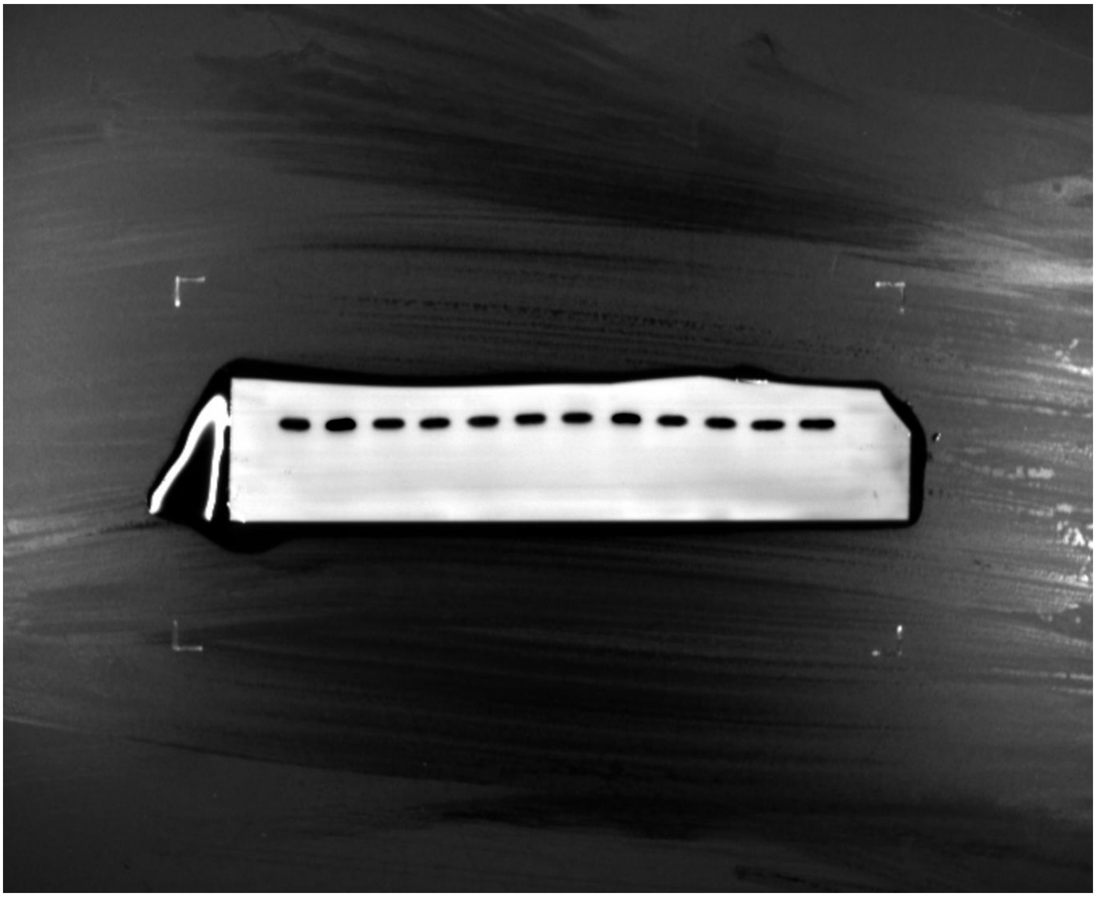

Figure 8E-GAPDH
